# Supplementary material for: Exercise for people living with frailty and receiving haemodialysis: a mixed-methods randomised controlled feasibility study
Source: BMJ Open. 2020 Nov 3;10(11):e041227. doi: 10.1136/bmjopen-2020-041227 (PMC7640592; doi:10.1136/bmjopen-2020-041227)
Supplement: Supplementary data [file bmjopen-2020-041227supp001.pdf]

Supplementary material 1. Inclusion and exclusion criteria for the *CYCLE-HD* trial.

Inclusion criteria	Exclusion criteria
Prevalent HD patient (> three months)	Unable to participate in current exercise programme due to perceived physical or psychological barriers
Aged 18 years or older	Unable to undergo MRI scanning (metal implants, severe claustrophobia)
Able and willing to give informed consent	Unfit to undertake exercise according to the American College of Sports Medicine (ACSM) guidelines
