# Supplementary material for: Exercise for people living with frailty and receiving haemodialysis: a mixed-methods randomised controlled feasibility study
Source: BMJ Open. 2020 Nov 3;10(11):e041227. doi: 10.1136/bmjopen-2020-041227 (PMC7640592; doi:10.1136/bmjopen-2020-041227)
Supplement: Supplementary data [file bmjopen-2020-041227supp002.pdf]

Supplementary material 2. Summary of intervention characteristics, in line with TiDier guidance.

**Description of intervention.**		A structured, supervised cycling exercise intervention delivered during in-centre HD.
**Rationale.**		• Intradialytic cycling provides aerobic and low-level resistance training, is associated with increased adherence and is most widely used within practice.
**What.**	Materials provided to participants or used to support intervention delivery.	• Cycling was delivered using the Moto Med Letto 2 (Medimotion Ltd).<br>• Materials: individualised exercise prescription and records of individual training bouts (duration (mins), intensity (RPE), resistance (gear), power output (watts) and energy expenditure (Kcal).<br>• General information on the benefits of exercise (posters and leaflets) available across all 3 HD centres.
	Materials used to train intervention providers.	Standardised progression and training protocol used by all providers.
**Who** (intervention providers).		• Qualified exercise professionals with experience of delivering exercise to renal patients.<br>• All providers were directly involved in the study, and not delivering the sessions as part of a clinical role.<br>• Roles included exercise provision, supervision, monitoring and progression.
**How** (mode of delivery).		One to one, face to face.
**Where** (location).		Three HD units across the East Midlands, UK.
**When and how much**	The frequency of delivery.	Thrice weekly during each dialysis session.
	Target intensity of each bout of exercise.	RPE 12-14 (moderate intensity), cadence 60-70 RPM.
	Target duration of each bout of exercise.	At least 30 minutes of continuous exercise.
	The total duration of delivery.	Six months, with a one-month run-in period to achieve the target exercise prescription.
**Tailoring.**		• The starting resistance (gear) based on the individual's tolerance.<br>• RPE used throughout to monitor and progress the exercise.<br>• Interval training was permitted.

Abbreviations: HD, haemodialysis; Kcals, kilocalories; RPE, rating of perceived exertion, RPM, revolutions per minute.
