# Supplementary material for: Exercise for people living with frailty and receiving haemodialysis: a mixed-methods randomised controlled feasibility study
Source: BMJ Open. 2020 Nov 3;10(11):e041227. doi: 10.1136/bmjopen-2020-041227 (PMC7640592; doi:10.1136/bmjopen-2020-041227)
Supplement: Supplementary data [file bmjopen-2020-041227supp003.pdf]

Supplementary material 3. A priori progression criteria based on the primary feasibility objectives.

**Eligibility**	**Stop**	Less than **20%** of all patients eligible
	**Go**	More than **50%** of all patients eligible
**Recruitment**	**Stop**	Less than **25%** of eligible patients recruited
	**Go**	More than **50%** of eligible patients recruited
**Exercise acceptability**	**Stop**	Less than **30%** adherence to the exercise sessions
	**Go**	More than **70%** adherence to the exercise sessions
**Outcome acceptability**	**Stop**	Less than **70%** outcome measure completion
	**Go**	More than **80%** outcome measure completion
**Loss to follow-up**	**Stop**	More than **40%** loss to follow-up
	**Go**	Less than **20%** loss to follow-up
