# Supplementary material for: Exercise for people living with frailty and receiving haemodialysis: a mixed-methods randomised controlled feasibility study
Source: BMJ Open. 2020 Nov 3;10(11):e041227. doi: 10.1136/bmjopen-2020-041227 (PMC7640592; doi:10.1136/bmjopen-2020-041227)
Supplement: Supplementary data [file bmjopen-2020-041227supp004.pdf]

Supplementary material 4. Patient-reported secondary outcome measures.

**Patient-reported secondary outcome**	**Construct measured**
12-item Short-Form Health Survey Version 2 (SF-12)	Generic health-related quality of life. Higher scores reflect better HRQoL.Scores are presented as a mental and physical component summary score.
Palliative care Outcomes Scale – Renal version (POS-R)	Renal specific measure of symptomology and symptom burden. A global symptom score was calculated by totalling all the scored items within the questionnaire. The mean number of symptoms, symptom severity was also calculated. Higher scores reflect greater symptom burden.
Hospital Anxiety and Depression Scale (HADS)	Emotional distress. A score of ≥14 indicates the presence of emotional distress in HD patients
The Exercise Self-Efficacy Scale (ESES)	Exercise confidence. Higher scores reflecting greater self-efficacy.
Dialysis Patient-Perceived Exercise Benefits and Barriers Scale (DPPEBBS)	HD patients' perceptions of benefits and barriers to exercise. Higher scores indicate a greater perception of the benefits of exercise over barriers.
The Dukes Activity Status Index (DASI)	Self-reported physical function. Higher scores indicate higher levels of physical function. The questionnaire was also used to estimate VO_2 peak.

HD, haemodialysis; HRQoL, Health-related quality of life.
