# Supplementary material for: Exercise for people living with frailty and receiving haemodialysis: a mixed-methods randomised controlled feasibility study
Source: BMJ Open. 2020 Nov 3;10(11):e041227. doi: 10.1136/bmjopen-2020-041227 (PMC7640592; doi:10.1136/bmjopen-2020-041227)
Supplement: Supplementary data [file bmjopen-2020-041227supp005.pdf]

Supplementary material 5. Interview topic guide question.

<u>Diary</u>

1. Can you tell me about how you have been using the diary?

2. If we asked patients to keep diaries like yours as part of a future study, what might help them?

3. [If applicable] I've had an opportunity to have a look through your diary. Could you tell me more about…?

<u>Exercise intervention for frailty and falls</u>

4. For some people exercising helps to prevent falls, make people more able and feel better. How do you feel about exercising?

5. Cycling during dialysis is thought to be a good way to exercise if you are on dialysis. Have you seen these bikes?

6. Programmes that are available for other people who fall include things like group exercise and education. What do you think about this?

7. These programmes usually take place at the hospital. What do you think about this?

8. Some people prefer to do their exercise at home. What do you think about this?

9. Where do you think a programme should be run?

10. How often do you think you would be able to exercise?

11. Would you want any support to help you exercise?

12. What might put you off exercising?

13. What questions might you have before you decide to take part or not?

14. If you did take part in some kind of exercise programme, what improvements would you most like to see?

<u>Research</u>

15. Have you ever been involved in research before? [Could tailor to involvement in CYCLE study (declined/ took part. If took part completed/dropped out) if patient unsure]

16. What do you think about the information you receive when deciding to take part in a research study?

17. Often researchers ask you to complete some assessments or tests to see if the thing they are studying is effective or not. What do you think would help patients to complete these assessments/ tests?

18. Sometimes people don’t complete the research study, which may happen for several reasons [give examples as needed]. What do you think would help keep dialysis from dropping out of research studies?

19. What would you like to happen once you reach the end of the study?
