# Supplementary material for: Exercise for people living with frailty and receiving haemodialysis: a mixed-methods randomised controlled feasibility study
Source: BMJ Open. 2020 Nov 3;10(11):e041227. doi: 10.1136/bmjopen-2020-041227 (PMC7640592; doi:10.1136/bmjopen-2020-041227)
Supplement: Supplementary data [file bmjopen-2020-041227supp006.pdf]

Supplementary material 6. Falls summary data and incidence of falls per person years.

	**Usual care** (n=27)	**Exercise** (n=24)
**Number of Falls**	11	5
**Number (% of group) of non-fallers**	19 (70)	20 (83)
**Number (% of group) fallers (≥ 1 fall)**	8 (30)	4 (17)
**Number (% of group) frequent fallers (≥2 falls)**	3 (11)	1 (4)
**Person years**	40.5	36
**Incidence rate**	0.27	0.14
