# Supplementary material for: Exercise for people living with frailty and receiving haemodialysis: a mixed-methods randomised controlled feasibility study
Source: BMJ Open. 2020 Nov 3;10(11):e041227. doi: 10.1136/bmjopen-2020-041227 (PMC7640592; doi:10.1136/bmjopen-2020-041227)
Supplement: Supplementary data [file bmjopen-2020-041227supp007.pdf]

Supplementary material 7. Changes in exercise capacity and physical function after six months.

	**Outcome**		**Usual Care**	**Exercise**	**Difference (95% CI)**
**ISWT** (m)		n	16	15	36 (-12 to 84)
		Baseline	184 ± 130	237 ± 173	
		Final	158 ± 154	248 ± 192	
		Change	-26 ± 68	11 ± 63	
**ESWT** (secs)		n	14	15	181 (-92 to 453)
		Baseline	347 ± 384	401 ± 375	
		Final	193 ± 304	428 ± 423	
		Change	-153 ± 286	27 ± 413	
**STS60** (n)		n	17	15	0 (-5 to 4)
		Baseline	10 ± 12	13 ± 11	
		Final	10 ±13	13 ± 12	
		Change	0 ± 7	0 ± 6	
**SPPB**	Total score	n	17	15	0.5 (-0.7 to 2)
		Baseline	7 ± 3	9 ± 3	
		Final	6 ± 2	8 ± 3	
		Change	-1 ± 2	-0.5 ± 1	
	4m walk time (secs)	n	17	15	1 (-1 to 4)
		Baseline	7 ± 6	4± 1	
		Final	6 ± 4	5 ± 2	
		Change	1 ± 5	0 ± 1	
	Gait speed (m/s)	n	17	15	0.05 (-0.12 to 0.22)
		Baseline	0.74 ± 0.29	0.96 ± 0.28	
		Final	0.74 ± 0.28	0.91 ± 0.31	
		Change	0.00 ± 0.22	-0.05 ± 0.24	
	STS5 (secs)	n	9	10	5 (-4 to 15)
		Baseline	17 ± 7	16 ± 14	
		Final	23 ± 13	16 ± 10	
		Change	6 ± 11	0 ± 8.	

Abbreviations: CI, confidence interval; ESWT, Endurance Shuttle Walk Test; ISWT, Incremental Shuttle Walk Test; m/s, metres per second; Secs, seconds; SPPB, Short Physical Performance Battery; STS5, Sit to Stand Five Repetitions; STS60, Sit to Stand in Sixty Seconds.
