# Supplementary material for: Exercise for people living with frailty and receiving haemodialysis: a mixed-methods randomised controlled feasibility study
Source: BMJ Open. 2020 Nov 3;10(11):e041227. doi: 10.1136/bmjopen-2020-041227 (PMC7640592; doi:10.1136/bmjopen-2020-041227)
Supplement: Supplementary data [file bmjopen-2020-041227supp008.pdf]

Supplementary material 8. Changes in physical activity (accelerometry data) after six months.

	Type of day		Usual Care	Exercise	Difference (95% CI)
**Waking wear time** (mins)	HD	n	5	10	244 (16 to 473)
		Baseline	891 ± 202	818 ± 183	
		Final	749 ± 105	921 ± 171	
		Change	-142 ±166	103 ± 204	
	Non-HD	n	5	10	170 (-13 to 353)
		Baseline	893 ± 90	927 ± 216	
		Final	817 ± 134	1022 ± 165	
		Change	-75 ± 201	95 ± 129	
**Steps** (steps/day)	HD	n	5	10	859 (-825 to 2543)
		Baseline	2252 ± 4210	1373 ± 1080	
		Final	2464 ± 4783	2444 ± 1904	
		Change	211 ± 593	1070 ± 1665	
	Non-HD	n	5	10	888 (-84 to 1861)
		Baseline	3076 ± 5790	2387 ± 1696	
		Final	2645 ± 5284	2845 ± 2117	
		Change	-430 ± 603	458 ± 903	
**Sedentary** (mins/ day)	HD	n	5	10	28 (-284 to 340)
		Baseline	954 ±338	954 ± 203	
		Final	965 ± 208	992 ± 182	
		Change	10 ± 200	38 ± 287	
	Non-HD	n	5	10	124 (-205 to 454)
		Baseline	1022 ± 357	1103 ± 253	
		Final	912 ± 224	1117 ± 174	
		Change	-110 ± 298	14 ± 269	
**Light PA** (mins/day)	HD	n	5	10	91 (23 to -158)
		Baseline	125 ± 51	83 ± 42	
		Final	79 ± 39	127 ± 73	
		Change	-46 ± 45	44 ± 62	
	Non-HD	n	5	10	9 (-71 to 91)
		Baseline	145 ± 59	133 ± 50	
		Final	154 ± 99	151 ± 59	
		Change	9 ± 108	18 ± 44	
**Moderate PA** (mins/day)	HD	n	5	10	13 (-32 to 57)
		Baseline	83 ± 105	29 ± 33	
		Final	85 ± 123	43 ± 55	
		Change	1 ± 52	14 ± 29	
	Non-HD	n	5	10	20 (40 to -79)
		Baseline	79 ± 96	46 ± 61	
		Final	75 ± 112	62 ± 105	
		Change	-4 ± 40	16 ± 55	
**Vigorous PA** (mins/day)	HD	n	5	10	3 (-1 to 8)
		Baseline	4 ± 9	1 ± 1	
		Final	1 ± 2	1 ± 3	
		Change	-3 ± 7	0 ± 2	
	Non-HD	n	5	10	1 (0 to 2)
		Baseline	3 ± 0	1 ± 4	
		Final	2 ± 5	1 ± 4	
		Change	-1 ± 2	0 ± 0	

Abbreviations: CI, confidence interval; HD, haemodialysis; mins, minutes; PA, physical activity.

Young HML, *et al. BMJ Open* 2020; 10:e041227. doi: 10.1136/bmjopen-2020-041227
