# Supplementary material for: Exercise for people living with frailty and receiving haemodialysis: a mixed-methods randomised controlled feasibility study
Source: BMJ Open. 2020 Nov 3;10(11):e041227. doi: 10.1136/bmjopen-2020-041227 (PMC7640592; doi:10.1136/bmjopen-2020-041227)
Supplement: Supplementary data [file bmjopen-2020-041227supp009.pdf]

Supplementary material 9. Patient-reported outcomes measures after six months.

	Outcome		Usual Care	Exercise	Difference (95% CI)
**SF-12**	PCS	n	19	19	0 (-4 to 5)
		Baseline	35 ± 9	35 ± 10	
		Final	36 ± 10	36 ± 10	
		Change	1 ± 7	1 ± 7	
	MCS	n	19	19	4 (-3 to 10)
		Baseline	43 ± 15	45 ± 13	
		Final	46 ± 13	45 ± 13	
		Change	4 ± 7	0 ± 12	
**HADS**		n	20	17	0 (-3 to 4)
		Baseline	16 ± 10	15 ± 9	
		Final	14 ± 10	13 ± 9	
		Change	-2 ± 5	-2 ± 6	
**POS-R**	Global severity score	n	20	18	2 (-3 to 7)
		Baseline	19 ± 14	19 ± 14	
		Final	18 ± 14	20 ± 14	
		Change	1 ± 6	-1 ± 9	
	mean severity	n	20	18	0 (0 to 0)
		Baseline	2 ± 1	2 ± 1	
		Final	2 ± 1	2 ± 1	
		Change	0 ± 0	0 ± 0	
	mean number	n	22	16	0 (-1 to 2)
		Baseline	9 ± 4	10 ± 4	
		Final	9 ± 4	10 ± 5	
		Change	0 ± 4	0 ± 2	
**ESES**		n	19	16	0 (-1 to 1)
		Baseline	2 ± 2	2 ± 1	
		Final	2 ± 1	2 ± 1	
		Change	0 ± 1	0 ± 1	
**DPPEBBS**		n	19	15	3 (-4 to 11)
		Baseline	59 ± 10	59 ± 15	
		Final	61± 10	65 ± 7	
		Change	2 ± 7	6 ± 14	
**DASI**		n	20	18	4.93 (-0.94 to 10.80)
		Baseline	13.06 ± 12.85	20.29 ± 14.33	
		Final	17.29 ± 14.41	19.60 ± 14.59	
		Change	4.22 ± 9.72	-0.71 ± 7.92	

Abbreviations: CI, confidence interval; DASI, Duke Activity Status Index; DPPEBBS, Dialysis Patients Benefits and Barriers Scale; ESES, Exercise Self efficacy Scale; HADS, Hospital Anxiety and Depression Scale; MCS, mental component summary score; POS-R, Palliative Outcomes Scale Renal, PCS, physical component summary score; VAS, visual analogue scale.
