# Supplementary material for: Exercise for people living with frailty and receiving haemodialysis: a mixed-methods randomised controlled feasibility study
Source: BMJ Open. 2020 Nov 3;10(11):e041227. doi: 10.1136/bmjopen-2020-041227 (PMC7640592; doi:10.1136/bmjopen-2020-041227)
Supplement: Supplementary data [file bmjopen-2020-041227supp010.pdf]

Supplementary material 10. Baseline demographic and clinical characteristics for the qualitative participants.

		**N=25**
**Age** (years)		69±10
**Gender** n (%)	Female	13 (52%)
	Male	12 (48%)
**Ethnicity** n (%)	White background	13 (52%)
	Asian or Asian British	10 (40%)
	Caribbean	1 (4%)
	Not stated	1 (4%)
**Diagnosis**	Diabetic nephropathy	11 (44%)
	Aetiology uncertain	6 (24%)
	Chronic pyelonephritis	3 (12%)
	Atypical hemolytic uremic syndrome	1 (4%)
	FSGS	1 (4%)
	Henoch-Schönlein Purpura	1 (4%)
	Minimal change nephropathy	1 (4%)
	Polycystic kidney disease	1 (4%)
**CCI**		6±2
**Time on HD** (months)		43 (IQR 16-85)
**CFS** n (%)	Vulnerable	9 (36%)
	Mildly frail	5 (20%)
	Moderately frail	8 (32%)
	Severely frail	3 (12%)
**Number of falls in the last six months**		3 (IQR 2-4)
**Previous transplant** n (%)	No	21 (84%)
	Yes	4 (16%)
**Active on transplant list** n (%)	No	22 (88%)
	Yes	3 (12%)

Abbreviations: CCI, Charlson comorbidity index; CFS, clinical frailty scale; FSGS, Focal segmental glomerulosclerosis; HD, haemodialysis.

Young HML, *et al. BMJ Open* 2020; 10:e041227. doi: 10.1136/bmjopen-2020-041227
