# Supplementary material for: Exercise for people living with frailty and receiving haemodialysis: a mixed-methods randomised controlled feasibility study
Source: BMJ Open. 2020 Nov 3;10(11):e041227. doi: 10.1136/bmjopen-2020-041227 (PMC7640592; doi:10.1136/bmjopen-2020-041227)
Supplement: Supplementary data [file bmjopen-2020-041227supp011.pdf]

Supplementary material 11. Joint display of quantitative and qualitative results, with an overall assessment of mixed-methods inferences.

	**Progression criteria**	**Feasibility trial**	**Qualitative results**	**Mixed-methods inferences**
**Eligibility**	**STOP** <20%<br>**GO** >50%<br>eligible.	31% patients eligible	No discussion. Patients not involved in screening process	Silence
**Recruitment**	**STOP** <25%<br>**GO** >50%<br>recruited.	52% eligible patients recruited.	- Frailer and female participants less likely to be approached despite eligibility and have more concerns about their suitability<br>- Perception that risks outweigh the potential benefits<br>- Recruitment processes could be improved	Complementary
**Retention**	**STOP** >40%<br>**GO** <20%<br>lost to follow-up.	12% loss to follow-up. Reasons predominantly unavoidable (death, ill-health).	Loss to follow-up attributed to:<br>- Illness;<br>- Length of trial;<br>- Study not meeting expectations.	Complementary
**Intervention**	**STOP** <30%<br>**GO** >70%<br>adherence over six-months.	74% adherence rate across the six-month exercise duration.	- IDC good use of time but limited in scope.<br>- Participants felt safe and felt well supported.<br>- Participants described a range of other important exercise components (see Figure 2)	Complementary
**Outcome**	**STOP** <70%<br>**GO** >80%<br>outcome measure completion.	Up to 89% of secondary outcome measure data missing<br>Collection of falls data challenging.	- Number of outcomes measured viewed as excessive.<br>- Outcome testing during HD or at home preferred.<br>- 52% agreed to complete a falls diary, falls not prioritised by participants.<br>- STS60, ESWT and ISWT unsuitable<br>- Researcher support and family involvement may increase outcome measure completion<br>- Outcomes measuring ADLs and participation in social roles prioritised	Complementary<br><br>Silence for PA monitoring.

Results from the feasibility trial are colour coded to depict whether they met the 'stop' (red), 'go' (green) or 'change (orange) progression criteria. Abbreviations: ADLs, activities of daily living; ESWT, Endurance Shuttle Walk Test; IDC, intradialytic exercise; ISWT, Incremental Shuttle Walk Test; PA, physical activity; STS60, sit to stand in sixty seconds.

Young HML, *et al. BMJ Open* 2020; 10:e041227. doi: 10.1136/bmjopen-2020-041227
