# Supplementary material for: Exercise for people living with frailty and receiving haemodialysis: a mixed-methods randomised controlled feasibility study
Source: BMJ Open. 2020 Nov 3;10(11):e041227. doi: 10.1136/bmjopen-2020-041227 (PMC7640592; doi:10.1136/bmjopen-2020-041227)
Supplement: Author's manuscript [file bmjopen-2020-041227.draft_revisions.pdf]

BMJ Open is committed to open peer review. As part of this commitment we make the peer review history of every article we publish publicly available.

When an article is published we post the peer reviewers' comments and the authors' responses online. We also post the versions of the paper that were used during peer review. These are the versions that the peer review comments apply to.

The versions of the paper that follow are the versions that were submitted during the peer review process. They are not the versions of record or the final published versions. They should not be cited or distributed as the published version of this manuscript.

BMJ Open is an open access journal and the full, final, typeset and author-corrected version of record of the manuscript is available on our site with no access controls, subscription charges or pay-per-view fees (http://bmjopen.bmj.com).

If you have any questions on BMJ Open's open peer review process please email info.bmjopen@bmj.com

BMJ Open

Exercise for people living with frailty and receiving haemodialysis: a mixed-methods randomised controlled feasibility study.

Journal:	*BMJ Open*
Manuscript ID	bmjopen-2020-041227
Article Type:	Original research
Date Submitted by the Author:	03-Jun-2020

Complete List of Authors:	Young, Hannah; University of Leicester, Department of Respiratory Sciences; University Hospitals of Leicester NHS Trust, Department of Research and Innovation<br>March, Daniel; University of Leicester, Department of Cardiovascular Sciences<br>Highton, Patrick; University of Leicester, Department of Cardiovascular Sciences; Loughborough University, National Centre for Sport and Exercise Medicine<br>Graham-Brown, Matthew ; University of Leicester, Cardiovascular Sciences; Loughborough University, National Centre for Sport and Exercise Medicine<br>Churchward, Darren; University of Leicester, Department of Cardiovascular Sciences<br>Grantham, Charlotte; University of Leicester, Department of Cardiovascular Sciences<br>Goodliffe, Samantha; University of Leicester, Department of Health Sciences<br>Jones, William; University Hospitals of Leicester, Emergency Department, Leicester Royal Infirmary,<br>Cheung, Mei-Mei; University Hospitals of Leicester, Renal, Respiratory and Cardiovascular Clinical Management Group<br>Greenwood, Sharlene ; King's College Hospital, Department of Physiotherapy and Renal Medicine; King's College London, Department of Renal Medicine<br>Eborall, Helen; University of Leicester, Department of Health Sciences<br>Conroy, Simon; University of Leicester, Department of Health Sciences<br>Singh, Sally; Leicester Biomedical Research Unit, Centre for Exercise & Rehabilitation Science; University Hospitals of Leicester NHS Trust, Department of Respiratory Medicine, Glenfield Hospital<br>Smith, Alice; University of Leicester, Department of Health Sciences<br>Burton, James; University of Leicester, Department of Cardiovascular Sciences; Loughborough University, National Centre for Sport and Exercise Medicine

Keywords:	Dialysis < NEPHROLOGY, End stage renal failure < NEPHROLOGY, REHABILITATION MEDICINE

I, the Submitting Author has the right to grant and does grant on behalf of all authors of the Work (as defined in the below author licence), an exclusive licence and/or a non-exclusive licence for contributions from authors who are: i) UK Crown employees; ii) where BMJ has agreed a CC-BY licence shall apply, and/or iii) in accordance with the terms applicable for US Federal Government officers or employees acting as part of their official duties; on a worldwide, perpetual, irrevocable, royalty-free basis to BMJ Publishing Group Ltd ("BMJ") its licensees and where the relevant Journal is co-owned by BMJ to the co-owners of the Journal, to publish the Work in this journal and any other BMJ products and to exploit all rights, as set out in our licence.

The Submitting Author accepts and understands that any supply made under these terms is made by BMJ to the Submitting Author unless you are acting as an employee on behalf of your employer or a postgraduate student of an affiliated institution which is paying any applicable article publishing charge ("APC") for Open Access articles. Where the Submitting Author wishes to make the Work available on an Open Access basis (and intends to pay the relevant APC), the terms of reuse of such Open Access shall be governed by a Creative Commons licence – details of these licences and which Creative Commons licence will apply to this Work are set out in our licence referred to above.

Other than as permitted in any relevant BMJ Author's Self Archiving Policies, I confirm this Work has not been accepted for publication elsewhere, is not being considered for publication elsewhere and does not duplicate material already published. I confirm all authors consent to publication of this Work and authorise the granting of this licence.

For peer review only

Exercise for people living with frailty and receiving haemodialysis: a mixed-methods randomised controlled feasibility study.

Hannah ML Young (MSc)[1,2], Daniel S March (PhD)[3], Patrick J Highton (PhD)[3,4], Matthew PM Graham-Brown (PhD)[3,4], Darren C Churchward (MSc)[3], Charlotte Grantham (BSc)[3], S Goodliffe (MSc)[5], William Jones (BAHons)[6], Mei-Mei Cheung (BAHons)[7], Sharlene A Greenwood (PhD)[8], Helen Eborall (PhD)[9], Simon Conroy (PhD)[5], Sally J Singh (PhD)[10,11], Alice C Smith (PhD)[5], James O Burton (MD)[3,4].

[1]Department of Respiratory Sciences, University of Leicester, Leicester, UK.

[2]Department of Research and Innovation, University Hospitals of Leicester, UK

[3]Cardiovascular Sciences, University of Leicester, UK.

[4]National Centre for Sport and Exercise Medicine, Loughborough University, UK.

[5]Department of Health Sciences, University of Leicester, UK.

[6] Emergency Department, Leicester Royal Infirmary, University Hospitals of Leicester, UK.

[7] Renal, Respiratory and Cardiovascular Clinical Management Group, University Hospitals of Leicester, UK.

[8]Department of Physiotherapy and Renal Medicine, King's College Hospital and Department of Renal Medicine, King's College London, UK.

[9]Social Science Applied to Healthcare Improvement Research (SAPPHIRE) Group, Department of Health Sciences, University of Leicester, UK.

For peer review only - http://bmjopen.bmj.com/site/about/guidelines.xhtml

[10]Centre for Exercise & Rehabilitation Science, Leicester Biomedical Research Unit, Glenfield Hospital, UK.

[11]Department of Respiratory Medicine, Glenfield Hospital, University Hospitals of Leicester,UK.

Corresponding author:

Hannah Young

Leicester Kidney Lifestyle Team

Academic Unit

Leicester General Hospital

Gwendolen Road

Leicester

UK

LE4 5PW

0116 258 4346

hmly1@le.ac.uk

Running title: Exercise for frail haemodialysis patients

Abstract word count: 268

Word count: 4300

For peer review only - http://bmjopen.bmj.com/site/about/guidelines.xhtml

ABSTRACT

Objectives

Frailty is highly prevalent in haemodialysis (HD) patients, leading to poor outcomes. This study aimed to determine whether a Randomised Controlled Trial (RCT) of intradialytic exercise is feasible for frail HD patients, and explore how the intervention may be tailored to their needs.

Design

Mixed-methods feasibility.

Setting & participants

Prevalent adult HD patients of the *CYCLE-HD* trial with a Clinical Frailty Scale Score of 4-7 (vulnerable to severely frail) were eligible for the feasibility study.

Interventions

Participants in the exercise group undertook six-months of thrice-weekly, progressive, moderate intensity intradialytic cycling (IDC).

Outcomes

Primary outcomes were related to feasibility. Secondary outcomes were falls incidence, exercise capacity, physical function, physical activity and patient-reported outcomes (PROMS) at baseline and six months. Acceptability of trial procedures and the intervention were explored via diaries and interviews with n=25 frail HD patients who both participated in (n=13, 52%), and declined (n=12, 48%), the trial.

Results

For peer review only

For peer review only - http://bmjopen.bmj.com/site/about/guidelines.xhtml

124 (31%) patients were eligible, 64 (52%) consented and 51 (80%) completed a baseline assessment. N=24 (71% male; 59 ± 13 years) dialysed during shifts randomly assigned to exercise and n=27 (81% male; 65 ± 11) assigned to usual care. N=6 (12%) were lost to follow-up. The exercise group completed 74% of sessions. 27 to 89% of secondary outcome data were missing. Frail HD patients outlined several ways to enhance trial procedures. Maintaining ability to undertake activities of daily living and social participation were outcomes of primary importance. Participants desired a varied exercise programme.

Conclusions

A definitive RCT is feasible, however a comprehensive exercise programme may be more efficacious than IDC in this population.

Trial Registration

ISRCTN11299707; ISRCTN12840463

Keywords: feasibility; frailty; exercise; haemodialysis; mixed-methods.

For peer review only

STRENGTHS AND LIMITATIONS OF THIS STUDY

- To our knowledge, this is the first study to evaluate the feasibility of an exercise intervention for people living with frailty and receiving haemodialysis (HD).
- The Clinical Frailty Scale, a frailty risk-stratification measure which has been validated in an HD population, was used to identify eligible participants.
- This study is also the first to explore how trial procedures and exercise programmes should be specifically tailored to the needs of people living with frailty and receiving HD, from their own perspectives.
- Multiple qualitative methods (interviews and diaries) were used to explore participants perceptions, providing a form of triangulation which strengthens the conclusions made.
- Due to the nature of the intervention and resource limitations, we could not blind intervention providers, outcome assessors or study participants to group allocation.

For peer review only - http://bmjopen.bmj.com/site/about/guidelines.xhtml

INTRODUCTION

Frailty, "a multidimensional syndrome of decreased physiological reserve leading to increased vulnerability to minor health stressors", is highly prevalent within the haemodialysis (HD) population[1,2]. Increasing frailty is associated with worsening outcomes, including mortality, hospitalisation, falls, reduced Health-Related Quality of Life (HRQoL), psychological well-being, physical function, ability to undertake activities of daily living (ADLs) and increased symptom burden[3-5].

Despite this, frailty is not static and evidence suggests that some factors associated with frailty are amenable to change[6]. Whilst the possible mediating role of exercise has been discussed, to our knowledge no original studies have examined the feasibility or effectiveness of an exercise programme for people living with frailty and receiving HD[7]. To date, exercise interventions for HD patients have focused upon intradialytic exercise, most commonly delivered by means of a cycle ergometer (intradialytic cycling, IDC), yet little is known about whether this is the most appropriate training stimulus for frail HD patients[8]. In addition, HD treatment can be poorly tolerated by frail patients and therefore IDC may represent an additional stressor to which these patients are particularly vulnerable[9]. European renal best practice guidance highlights a need for studies which identify how exercise programmes should be more specifically tailored to the needs of frail CKD patients[10], yet to date, there has also been no exploration of the needs, barriers and facilitators to exercise from the perspectives of people living with frailty and receiving HD themselves.

The aim of this study was to determine the feasibility of conducting an RCT investigating the effects of IDC for HD patients living with frailty by: (i) estimating rates of eligibility,

For peer review only - http://bmjopen.bmj.com/site/about/guidelines.xhtml

recruitment, retention, exercise adherence and outcome acceptability; and exploring (ii) the potential benefits of IDC across a range of secondary outcomes; and (iii) the perceptions of frail HD patients in relation to participating in clinical research, IDC and a tailored exercise intervention.

METHODS

Design

A mixed-methods, prospective, randomised feasibility study was conducted alongside concurrent qualitative diaries and interviews (Trial Registration numbers ISRCTN11299707; ISRCTN12840463). The feasibility study was a secondary analysis of the *CYCLE-HD* trial, whose aims and methods are reported elsewhere[11]. The qualitative component was underpinned by a constructivist Grounded Theory approach[12]. All participants provided written informed consent.

Participants

Prevalent adult (over 18 years) HD patients were recruited from three centres within the UK East Midlands Renal Network. In addition to the inclusion and exclusion criteria for the *CYCLE-HD* trial (supplementary material 1), the Clinical Frailty Scale (CFS), a risk stratification tool validated in a HD population, was used to identify vulnerable to severely frail participants (CFS score 4-7)[13]. The inclusion and exclusion criteria for the qualitative component mirrored the feasibility study and both those involved in the trial, and those who were eligible but declined to participate were eligible.

For peer review only - http://bmjopen.bmj.com/site/about/guidelines.xhtml

Randomisation

HD cohorts were randomised prior to screening, based on a computer-generated randomisation algorithm held by the Robertson Centre for Biostatistics at the University of Glasgow.

Recruitment

Patients were screened for eligibility by their supervising nephrologist. Suitable patients were approached during HD, and the study explained. For the qualitative component, participants who had been involved in the feasibility study were recruited following completion of, or withdrawal from, the trial to prevent contamination.

Exercise intervention

Supplementary material 2 outlines the exercise intervention in line with TIDieR guidance[14]. Briefly, following a one-month run-in, participants in the exercise group undertook thrice-weekly supervised, moderate-intensity (Rating of Perceived Exertion, RPE 12-14) IDC (MOTOmed Letto2, Reck, Germany), for six months[15]. Cycling resistance was progressively increased to maintain RPE in response to exercise adaptation. Both arms continued with usual care HD as described elsewhere[11].

Sample size

Determinations of sample size from a power calculation around a primary outcome are not relevant to a feasibility study and sample sizes of 24-50 are considered sufficient[16]. For the

For peer review only - http://bmjopen.bmj.com/site/about/guidelines.xhtml

qualitative component maximum variation sampling was initially used to ensure diversity in frailty status and level of trial participation[12]. As understanding was gained from preliminary analyses, theoretical sampling was used to further recruit participants[12]. A maximum of 30 interviews were planned, but data collection ceased at the point where theoretical categories were saturated and no longer generated new insight (n=25).

Primary outcome measures

The primary feasibility outcomes are presented in supplementary material 3. Judgement regarding feasibility was based upon a set of *a priori* progression criteria. For each criterion, the development of 'stop'(indicating when there are issues with the trial that cannot be resolved) and 'go' thresholds (when there are no issues that may impede the success of a trial) were co-produced by patients, clinicians and researchers[17,18]. Results falling between these thresholds indicated that adaptation to trial procedures may render a definitive RCT viable[18].

Baseline demographic and clinical variables

Demographic and clinical characteristics were gathered from participants' medical notes. The Charlson Comorbidity Index (CCI) was used to estimate the burden of comorbid disease[19].

Secondary outcome measures

Multiple secondary outcomes were used to determine the potential effects of IDC and most appropriate primary endpoint for a future RCT. Outcome assessors were not blinded to group allocation.

For peer review only - http://bmjopen.bmj.com/site/about/guidelines.xhtml

Information on the number of falls, defined as 'an unexpected event in which the participants come to rest on the ground, floor, or lower-level' which resulted in Emergency Department visits and hospital admissions were collected from baseline to one year following intervention completion from medical records and hospital episode statistics[20].

Field tests of exercise capacity and physical function included the Incremental Shuttle Walk Test (ISWT), the Endurance Shuttle Walk Test (ESWT), the Short Physical Performance Battery (SPPB) and the Sit-to Stand in Sixty Seconds (STS60)[11]. Physical activity (PA) was objectively measured using the SenseWear Armband (SWA) Pro 3 (BodyMedia, Inc., Pittsburgh PA, USA) for seven consecutive days, including HD. Established criteria were used to ensure representative data for average daily wear-time, steps per day, and time (minutes per day) spent in sedentary (defined as 0-1.5 METS), light (1.6-2.9 METS) moderate (3-6 METS) and vigorous (>6 METS) PA[21]. PROMs collected are outlined in supplementary material 4[11]. All outcomes were collected at baseline and six months.

Serious adverse events (SAEs) were recorded and assessed from baseline to six-months as outlined previously[11].

Diaries and interviews

Participants first completed a prospective falls diary, recognised as the current 'gold standard' for falls data collection, for up to three months to examine the feasibility of this outcome measure within a future definitive RCT[20]. Semi-structured interviews then explored

For peer review only - http://bmjopen.bmj.com/site/about/guidelines.xhtml

participants' experiences of: (i) keeping a falls diary; (ii) participating in a trial; and (iii) their perceptions of IDC and a tailored exercise intervention.

Information to support diary collection and a topic guide for the interviews (supplementary material 5) was developed by HMLY, HE and a patient and public involvement group. Topics were tailored according to the level of involvement in the trial, and the content of diaries. Interviews were conducted during HD, in the participant's home, or in the hospital by HMLY and lasted 20 to 120 minutes (mean 63 minutes). All were digitally audio-recorded and transcribed verbatim.

Data analysis

Descriptive statistics and confidence intervals were used to estimate feasibility outcomes[23]. The percentage of exercise sessions completed was used to establish the acceptability of IDC. Outcome acceptability was determined by quantifying the amount of missing data across secondary outcomes. No imputation was performed to account for missing data. No statistical testing relating to the efficacy of the exercise intervention was undertaken, although the potential benefits of exercise were estimated[23]. For falls, incident rate ratio and 95% confidence intervals were presented. Statistical analyses were performed using SPSS 24 (IBM UK Ltd, UK).

Qualitative analysis was undertaken by HMLY and SG and informed by a constant comparative approach[12]. Transcripts were reviewed, then coded line by line, followed by focused, and then theoretical, coding[12]. NVivo11 software (QSR International Ltd, version

For peer review only - http://bmjopen.bmj.com/site/about/guidelines.xhtml

11, 2016) was used to facilitate data management. Finally, qualitative and quantitative results were merged in a 'joint display' to facilitate an overall assessment of feasibility[24].

Patient and public involvement

The patient and public involvement (PPI) group for this study comprised patients of all ages, genders and ethnicities who were living with frailty and receiving HD, and their relatives. They agreed this study was an important priority for further investigation and particularly stressed the need to add the qualitative component. The PPI group were involved early in ethical approval stages and were actively engaged in writing lay summaries and providing patient perspectives on data collection procedures, ethical issues, and the study dissemination plans. They assisted in the preparation of study documentation, interview topic guides and diary keeping materials. During the study, members of the PPI group attended regular steering meetings and were involved in co-producing the progression criteria.

RESULTS

Feasibility study

Eligibility and recruitment

Screening and recruitment took place from March 2015 to 2018, with data collection completed by November 2018. Figure 1 outlines the trial CONSORT. Of the 406 patients screened in the *CYCLE-HD* trial, n=124 (30%, 95% CI 26.1% to 35.3%) were identified as vulnerable to severely frail and therefore eligible for the feasibility study. Sixty-four participants (52%, 95% CI 42.5% to 60.7%) consented. Reasons for declining were lack of time or family support and reluctance to undergo outcome testing, or to be randomised.

For peer review only - http://bmjopen.bmj.com/site/about/guidelines.xhtml

250 Thirteen (20%, 95% CI 11.3% to 32.2%) participants withdrew prior to baseline assessment.
251 N=51 (80%, 95% CI 67.8% to 88.7%) completed this assessment. Twenty-four (47%)
252 participants received dialysis during shifts randomised to exercise and twenty-seven (53%)
253 during shifts randomised to usual care.

254

255 *Participant characteristics*

256 Table 1 displays the characteristics of the trial participants at baseline. Groups were well
257 matched across most variables. A lower proportion of participants were female (23.5%) and
258 severely frail (6%) overall.

For peer review only

For peer review only - http://bmjopen.bmj.com/site/about/guidelines.xhtml

Table 1. Baseline demographic and clinical characteristics of the trial participants.

		Usual care (n=27)	Exercise (n=24)	All (n=51)
**Age** (years)		65 ± 11	59 ± 13	63 ± 12
**Sex** (n, %)	Female	5 (19%)	7 (29%)	12 (23.5%)
Ethnicity (n, %)	White	12 (44%)	11 (41%)	23 (45%)
	Asian or Asian British	11 (41%)	11 (46%)	22 (43%)
	Caribbean	1 (4%)	0 (0%)	1 (2%)
	Other ethnic	1 (4%)	1 (4%)	2 (4%)
	Not stated	2 (7%)	1 (4%)	3 (6%)
Diagnosis (n, %)	Aetiology Uncertain	8 (29%)	7 (29%)	15 (29%)
	Diabetic Nephropathy	5 (19%)	7 (29%)	12 (23%)
	Glomerulonephritis	5 (19%)	3 (14%)	8 (16%)
	Renal Vascular Disease	3 (11%)	2 (8%)	5 (10%)
	Other diagnoses	4 (15%)	1 (4%)	5 (10%)
	Chronic Pyelonephritis	2 (7%)	1 (4%)	3 (6%)
	Polycystic Kidney Disease	0 (0%)	2 (8%)	2 (4%)
	Not recorded	0 (0%)	1 (4%)	1 (2%)
CCI		5 ± 2	5 ± 2	5 ± 2
Previous transplant (n, %)	No	21 (75%)	18 (75%)	39 (76.5%)
	Yes	6 (21%)	6 (25%)	12 (23.5%)
Time on HD (months)		17 (7-53)	13 (10-61)	16 (8-53)
**BMI** (kg/m^2)		27.38 ± 6.72	25.87 ± 5.28	26.67 ± 6.07
Total no. medications		12 ± 4	12 ± 4	12 ± 4
Clinical Information	Albumin (g/L)	35.4 ± 4.4	37.4 ± 4.3	36.4 ± 4.4
	Haemoglobin (g/L)	107 ± 12	112 ± 17	107 ± 15
Haemodialysis	URR (%)*	74 (70-80)	75 (58-79)	74 (71-79)
	SBP (mmHg)	143 ± 21	144 ± 21	144 ± 21
	DBP (mmHg)*	65 (62-78)	78 (69-86)	76 (62-81)
**CFS** (n, %)	Vulnerable	13 (48%)	10 (42%)	23 (45%)
	Mildly frail	5 (18.5%)	7 (29%)	12 (23.5%)
	Moderately frail	8 (30%)	5 (21%)	13 (25.5%)
	Severely frail	1 (3.5%)	2 (8%)	3 (6%)

*Values reported are mean and SD (±), except for *median and IQR. Abbreviations: BMI, body mass index; CCI, Charlson Comorbidity Index; CFS, Clinical Frailty Scale DBP, diastolic blood pressure; SBP, systolic blood pressure; URR, urea reduction ratio*

For peer review only - http://bmjopen.bmj.com/site/about/guidelines.xhtml

Retention

Six (12%, 95% CI 4.4% to 23.9%) participants were lost to follow-up: three participants withdrew due to ill-health, one moved away, one changed HD regime and one withdrew consent.

Exercise adherence

A mean of 61±17 exercise sessions were completed over the six-month intervention, representing an adherence rate of 74±20%. The most frequent reasons for missing an exercise session were declining (n= 175 out of 535 sessions omitted in total, 33%), feeling unwell (n= 116, 22%) and pain (n= 105, 20%). Table 2 summarises the mean amount of exercise achieved. On average, participants reached the prescribed level of exercise by six months, although n=18 (75%) were unable to achieve this by the end of the one-month run-in period.

Table 2 Mean (SD) exercise achieved per session over the six-month duration of the intervention.

Duration (mins)	35 ± 8
Speed (RPM)	63 ± 10
Intensity (RPE)	13 ± 1
Gear	9 ± 4
Distance (Miles)	7 ± 3
**Power** (Watts)	13 ± 6
Energy expenditure (Kcals)	64 ± 31

All data presented as mean and SD (±). Abbreviations: kcals, kilocalories, mins, minutes; RPE, rating of perceived exertion; RPM, revolutions per minute.

Outcome acceptability

For tests of exercise capacity (ISWT and ESWT); n=14 (27%) did not complete at least one test at baseline, n=30 (64%) at interim and n=26 (58%) at final. For tests of physical function; n=20 (39%) did not complete at least one test at baseline, n=33 (70%) at interim and n=30 (67%) at final. For PROMs; n=27 (53%) did not complete at least one questionnaire at

For peer review only

For peer review only - http://bmjopen.bmj.com/site/about/guidelines.xhtml

baseline, n=27 (57%) at interim and n=40 (89%) at final. For PA data; n=21 (41%) were missing at baseline, and n=26 (58%) were missing at the final assessment. Declining was the primary reason for non-completion for all outcomes across all time points.

Secondary outcomes

Summary falls data are presented in supplementary material 6. The crude falls incident rate ratio (IRR) was 1.95 (95% CI 0.63 to 7.18), suggestive of an almost two-fold increased incidence of falls within the usual care group.

Exercise capacity was maintained in the exercise group, but deteriorated in the usual care group, resulting in an overall difference of 36m (95% CI -12 to 84) in ISWT results and 181 seconds (95% CI -92 to 453) in EWST time. The time taken to complete the STS5 also increased in the usual care group (suggesting a deterioration in function), but was maintained in the exercise group, resulting in an overall difference of 5 seconds (95% CI -4 to 15) (supplementary material 7).

Step count increased in the exercise group by 859 steps/day (95%CI -825 to 2543) on HD days and 888 steps/day (95%CI -84 to 1861) on non-HD days. Whilst sedentary time was increased in the exercise group on all days compared with the usual care group, this appeared to be offset by increases in light PA and moderate PA, and maintenance (albeit of low levels) of vigorous PA versus maintenance or deterioration across the same metrics in the usual care group (supplementary material 8). For PROMs, outcomes were largely unchanged, except for the DASI score, which appeared to deteriorate in the exercise group and increase in the usual care group, resulting in an overall difference in score of 4.93 (95% CI -0.94 to 10.80) and the

For peer review only - http://bmjopen.bmj.com/site/about/guidelines.xhtml

mental component summary score of the SF12 which improved in the usual care group, resulting in an overall difference in score of 4 (95% CI -3 to 10). Exercisers appeared to have a greater perception of the benefits of exercise compared with those in the control group (3, 95% CI -4 to 11) (supplementary material 9).

Serious adverse events

In total, n=13 (25%) experienced an SAE during the feasibility study, n=8 (33%) in the exercise group and n=5 (19%) in the usual care group. All events resolved, and none were directly related to the intervention or trial.

Qualitative findings

Thirty-seven patients were approached for the qualitative study. Twenty-six were recruited and one died prior to data collection. Thirteen had participated in the feasibility trial. Nine received dialysis during shifts randomised to exercise, and four randomised to usual care. Twelve participants had declined to take part in the feasibility trial. Full characteristics for the qualitative sample are provided in supplementary material 10.

In addition to categories relating to the feasibility outcomes, categories relating to both the delivery and the characteristics of a tailored exercise intervention were identified. These are presented alongside illustrative quotes within Tables 3, 4, 5 and 6 and Figure 2.

For peer review only - http://bmjopen.bmj.com/site/about/guidelines.xhtml

Feasibility and acceptability of a definitive trial

Eligibility and recruitment

Declining to participate was underpinned by a perception that the trial could worsen overall health, particularly amongst those who had not previously participated in research or had recently commenced HD. Female participants believed that exercise was predominantly for men and that they were already doing enough daily activity, whilst participants living with moderate to severe frailty viewed ageing as an inevitable decline unlikely to be influenced by exercise. Motivators included a sense of altruism, and the perception that participation could provide opportunities to improve individual outcomes; learn about their own health; and access better healthcare. Participants felt that recruitment could be enhanced by the effective use of non-verbal communication, rapport building, and actively involving family members in the recruitment process, as family support was often a prerequisite to participation (Table 3).

Trial retention

The primary reasons for withdrawal were becoming unwell, the duration of the trial and the research not meeting participants expectations. Participants suggested that having a rapport and maintaining regular dialogue with the research team might help retain participants within a future trial (Table 3).

For peer review only - http://bmjopen.bmj.com/site/about/guidelines.xhtml

349 *Table 3. Categories relating to trial eligibility, recruitment and retention with illustrative*
350 *quotes.*

For peer review only

The acceptability of IDC

IDC was generally perceived to be a safe and positive use of HD treatment time. However, IDC was described as limited in scope, and participants were uncertain of its impact, particularly upon mobility, symptoms and falls (Table 4).

Table 4. Categories relating to the acceptability of IDC outcome acceptability and illustrative quotes.

Outcome acceptability

As indicated by participant quotations in Table 5, the number of outcomes and follow-ups needed to be reduced and participants had a strong preference for outcomes that could be collected during HD treatment. Many found the ISWT and STS60 assessments too challenging. Participants were occasionally uncertain of the purpose of the questionnaires and many reported difficulty quantifying symptom severity or a desire to provide 'anticipated' responses.

Maintaining mobility, and the ability to undertake a range of ADLS and social roles were viewed as key outcomes for a future trial. Only thirteen (52%) participants in the qualitative study agreed to complete a falls diary and many reported they preferred falls information to be collected during HD treatment. The majority who had fallen rarely reported them to healthcare professionals, believing that they were an expected consequence of HD or having had experience of their concerns about falls being overlooked. Consequently, falls prevention was not viewed as a key outcome.

For peer review only - http://bmjopen.bmj.com/site/about/guidelines.xhtml

374 *Table 5. Categories relating to outcome acceptability and illustrative quotes.*

For peer review only

For peer review only - http://bmjopen.bmj.com/site/about/guidelines.xhtml

Perceptions of a tailored exercise programme

Delivery

There was no universally acceptable setting for exercise delivery (Table 6). Vulnerable and mildly frail participants (CFS 4-5) were particularly open to group-based exercise in the community or gym, which they felt would provide motivation through camaraderie with others. However, access barriers due to HD treatment, complex health needs, and lack of transport were common. Participants also described feeling self-conscious exercising amongst 'normal' people. Home-based exercise was preferred by those with moderate to severe frailty (CFS 6-7) due to easier access, greater flexibility and relevance to their daily activities. Despite this, concerns about lack of space and safety were highlighted by those who lived alone, whilst those with family were concerned about overburdening or injuring them by asking for support.

For peer review only

Table 6. Participants perceptions of the facilitators and barriers to group and home-based exercise.

For peer review only

For peer review only - http://bmjopen.bmj.com/site/about/guidelines.xhtml

Characteristics of a tailored exercise programme

Irrespective of the setting for delivery, participants identified several key features of a tailored exercise intervention which are summarised in Figure 2.

Preparation

Participants lived with a range of debilitating symptoms, most frequently fatigue, pain and dyspnoea. Often daily activity alone was felt to be enough of a challenge. Common impacts of exercise (for example breathlessness whilst exercising) were interpreted as worsening symptoms or damage, and many participants were uncertain if exercise would be suitable or beneficial. They indicated that the reason for exercising needed to be sufficiently compelling. They wanted to know what to expect prior to exercising, and individualised goal setting was advocated to build motivation and appreciate improvements.

Content

Key components described were whole body resistance, aerobic and balance training. Many participants described being unable to get up once they had fallen and felt that practising this was also important. Routine physical activity was viewed as more purposeful than structured exercise ‘for the sake of it’ and participants spoke of their enjoyment of being outside and engaging in meaningful and physically active hobbies.

Structure

Supervision was viewed as essential to select, teach and progress exercises. Individual tailoring which considered the impact of disability, comorbidities and fluctuating symptoms was important, and a choice of exercises, for example swimming, dancing and yoga, was

For peer review only - http://bmjopen.bmj.com/site/about/guidelines.xhtml

associated with increased enjoyment and engagement. Moderate to severely frail participants wanted the programme to be progressed in a supportive and collaborative manner. Those who were vulnerable or mildly frail wanted to be 'pushed' and progressed in a more assertive manner.

Having a companion (typically peers, family or friends) was viewed as helping to overcome access barriers and provide socialisation and mutual motivation. The sharing of experience was also seen as a powerful means of challenging preconceptions about exercise ability, although participants with moderate to severe frailty raised concerns about feeling embarrassed or 'judged' if they were less able.

Integrated mixed-methods analyses

The integrated qualitative and quantitative findings suggest that an RCT of IDC is feasible for frail HD patients following adaptation. However, IDC should not be the only intervention offered and the development of a multicomponent programme is warranted (Supplementary material 11).

DISCUSSION

These results suggest that an RCT of IDC is feasible for frail HD patients with adaptation to increase outcome acceptability and eligibility rates. Adherence to IDC was high and it was viewed as a safe and efficient use of HD treatment time. Secondary outcomes also suggest that, for HD patients with a CFS of 4-7, IDC may mitigate deterioration in exercise capacity, endurance and functional muscle strength and increase PA behaviour (steps/day). Despite

For peer review only - http://bmjopen.bmj.com/site/about/guidelines.xhtml

this, participants described a preference for a multi-component programme that prepared them for exercise, offered variety, companionship and individualised supervision. No single preferred environment for the delivery of this intervention was identified but appeared to be influenced by frailty grade and individual factors.

27% to 89% of secondary outcome measure data were missing, and, overall, this progression criterion was not achieved. Given that secondary measures are often insufficiently powered, reducing the number collected within a future trial may improve completion[27]. Falls were not of primary importance to participants, and aligns with the SONG-HD data which did not identify falls as a key outcome[29]. Our findings suggest that accurately capturing prospective falls data may be challenging due to under-reporting, and yet, retrospective falls data collection does not fully reflect the incidence and impact of falls. Given the high incidence of falls in this population, capturing falls data may be important in a future trial, and researcher-led prospective data collection at the dialysis unit is recommended, in line with participant feedback[5]. Further exploration and validation of meaningful measures for HD patients living with frailty is also warranted. Some of the functional measures (the STS60 and ISWT) included were too challenging and measures of independence, rarely used in exercise studies to date, were highlighted as important within this study, and have also been included in guidelines and core outcomes sets for HD and older people[28-30].

The results of this study indicate that changes to eligibility criteria and screening are required. As only patient participants were interviewed, it was not possible to gain any insight on this aspect of feasibility from the qualitative component. Importantly, the challenges of identifying eligible participants do not appear to be unique to this study. Studies of older

For peer review only - http://bmjopen.bmj.com/site/about/guidelines.xhtml

people living with frailty highlight that large numbers need to be screened to achieve a 50% recruitment rate, and a multicentre trial may be required[27].

This study suggests that IDC may reduce falls incidence in frail HD patients potentially by attenuating a decline in exercise capacity, physical activity behaviour and function at levels shown to be clinically meaningful in other long-term conditions[31,32]. This indicates that preventing deterioration may be as valuable, and more attainable, as improving outcomes in a frail population. Despite this, frail participants experienced difficulties achieving the proposed level of exercise and maintaining motivation in the face of varying symptomology. Exercise programmes have a dose-response, and these factors may have reduced participants physical capability to exercise and achieve optimal benefit, despite the overall good level of adherence. Clinical decision support tools have been used in other populations to rationalise exercise prescription, progression and amendment in the presence of varying symptomology, and a similar approach may be beneficial for frail HD patients[33].

This study indicates that participants desire a multicomponent exercise programme, and require an intervention that addresses their particularly low levels of PA. Whilst step count and time spent in light and moderate PA increased following IDC, these were below PA recommendations for older people[34]. To date, PA interventions for HD patients have predominantly centred around walking, which may not be appropriate for those living with frailty[35-38]. This study suggests that functional training (task-orientated exercises which engages multiple muscle groups) and physical activity that focuses on ‘doing more’ of these usual tasks may be more acceptable and efficacious. To date, two studies have employed similar approaches with non-frail HD patients. One study demonstrated significant

For peer review only - http://bmjopen.bmj.com/site/about/guidelines.xhtml

improvements in lower extremity performance and the other a non-significant improvement in physical function and maintenance of other SF-36 domains compared with the control group[39,40]. In older people without CKD who are living with frailty, functional training included as part of a multicomponent exercise programme is beneficial across a range of outcomes, including greater ability to rise from the floor following a fall [38,41-44]. A similar approach to exercise prescription may be warranted in a frail HD population.

Numerous barriers and facilitators to exercise were identified within this study, which have implications for the design of a programme. The use of theory is crucial in the development of effective interventions and the behaviour change wheel (BCW) is most frequently cited in the development of interventions in CKD[45]. Mapping the identified barriers and facilitators to the BCW indicates that ameliorating symptom burden prior to exercise, individualised exercise counselling, and a collaborative, problem-solving approach to exercise education are most likely to encourage and sustain participation[45,46]. Devising ways in which peer and family involvement can be incorporated into the programme may also increase motivation and opportunity to exercise but should be carefully managed given the potential for negative comparison amongst the frailest patients.

A lack of preferred environment for intervention delivery may have implications for a definitive RCT. Exercise interventions require motivation, and limited engagement may negatively influence a trials external and internal validity. Ignoring patient preference is also out of step with clinical practice, where rehabilitation involves shared decision-making. Taken together, these factors have implications for determining treatment effects and future intervention implementation[47]. There is increasing recognition that novel trial designs may be

For peer review only - http://bmjopen.bmj.com/site/about/guidelines.xhtml

indicated when evaluating complex interventions and a Partially Randomised Patient Preference Trial, where participants without preference are randomised whilst those with a preference receive their choice, would provide information on both the efficacy of the intervention and the influence of preference[47,48].

Strengths and limitations

To our knowledge, this study is the first to examine the feasibility of an RCT of IDC for frail HD patients and to explore how trial procedures and exercise programmes should be specifically tailored to the needs of this group, from their own perspectives.

Key strengths were the use of a validated frailty risk-stratification measure and multiple qualitative methods which provided a form of triangulation[49]. There were, however, challenges to recruiting severely frail participants to both the trial and the qualitative arms. Additionally, the views of clinicians and researchers were not explored. A future RCT should also blind outcome assessors to group allocation to reduce the potential for detection bias.

Conclusion

In summary, this study suggests that a future definitive trial of IDC is feasible within a HD population with a CFS of 4-7 and paying particular attention in the design to those factors mentioned above may facilitate improved rates of eligibility and outcome completion. Outcomes focusing on independence and participation should be the primary outcomes of interest in a future trial. Whilst an exploratory analysis suggests some potential benefits to IDC, a tailored intervention comprising a comprehensive multi-component programme,

For peer review only - http://bmjopen.bmj.com/site/about/guidelines.xhtml

symptom management, education and behaviour change is better suited to frail HD patients' needs.

ACKNOWLEDGEMENTS

The authors would like to thank the staff and patients at all units, Fresenius Medical Care and The Leicester Kidney Care Appeal. The authors also wish to thank Freya Tyrer for sharing her statistical expertise.

COMPETING INTERESTS

The authors have no conflicts of interest to declare.

FUNDING

The work was funded by the National Institute for Health Research (NIHR) Leicester Biomedical Research Centre, Collaboration for Leadership in Applied Health Research and Care East Midlands (CLAHRC EM) and partly funded by the Stoneygate Trust. H.M.L.Y. and J.O.B. are supported by grants from the NIHR (DRF-2016-09-015 and CS-2013-13-014). S.J.S. is supported by the Collaboration for Leadership in Applied Health Research and Care East Midlands. The views expressed in this publication are those of the authors and not necessarily those of the NHS, the NIHR or the Department of Health and Social Care. The funders had no role in the study design; collection, analysis, and interpretation of the data;

AUTHORS CONTRIBUTIONS

For peer review only

Literature search: HMLY; Research idea and study design: HMLY, JOB; participant recruitment: HMLY, DS, PH, DC, MPMGB, JOB, CG; data acquisition: HMLY, DS,PH,DC, MPMGB,CG WJ, MC; clinical governance: JOB; data analysis: HMLY, SG; statistical analysis: HMLY; supervision and mentorship: SC, HE, SG, SJS, ACS, JOB; manuscript preparation: HMLY; reviewed final manuscript: all. Each author contributed important intellectual content during manuscript drafting or revision and accepts accountability for the overall work by ensuring that questions pertaining to the accuracy or integrity of any portion of the work are appropriately investigated and resolved. All authors have read and approved the final version

ETHICAL APPROVAL

This study was approved by the East Midlands (Northampton; REC ref: 14/EM/1190) and South West (Bristol; REC ref: 17/SW/0048) NHS Research Ethics Committees for the trial and the qualitative component respectively

DATA SHARING STATEMENT

The datasets used and analysed during the current study are available from the corresponding author on reasonable request.

For peer review only

For peer review only - http://bmjopen.bmj.com/site/about/guidelines.xhtml

569 **REFERENCES**

570 1. Clegg A, Young J, Iliffe S, et al Frailty in elderly people. *The Lancet*.
571 2013;381(9868):752-762.

572 2. Zhao Y, Liu Q, Ji J. The prevalence of frailty in patients on hemodialysis: A systematic
573 review and meta-analysis. *Int Urol Nephrol*. 2019:1-6.

574 3. Iyasere OU, Brown EA, Johansson L, et al. Quality of life and physical function in older
575 patients on dialysis: A comparison of assisted peritoneal dialysis with hemodialysis. *Clin J*
576 *Am Soc Nephrol*. 2016;11(3):423-430.

577 4. Johansen KL, Chertow GM, Jin C, et al. Significance of frailty among dialysis patients.
578 *Journal of the American Society of Nephrology*. 2007;18(11):2960-2967.

579 5. López-Soto PJ, De Giorgi A, Senno E, et al. Renal disease and accidental falls: A review
580 of published evidence. *BMC nephrology*. 2015;16(1):176.

581 6. Johansen KL, Dalrymple LS, Delgado C, et al. Factors associated with frailty and its
582 trajectory among patients on hemodialysis. *Clinical Journal of the American Society of*
583 *Nephrology*. 2017;12(7):1100-1108.

584 7. Wang CJ, Johansen KL. Are dialysis patients too frail to exercise? Seminars in
585 dialysis.2019;32(4) 1-6.

586 8. Young HML, March DS, Graham-Brown MP, et al. Effects of intradialytic cycling
587 exercise on exercise capacity, quality of life, physical function and cardiovascular measures
588 in adult haemodialysis patients: A systematic review and meta-analysis. *Nephrology Dialysis*
589 *Transplantation*. 2018; 33(8),1436-1445.

For peer review only - http://bmjopen.bmj.com/site/about/guidelines.xhtml

9. Brown EA. Can quality of life be improved for the increasing numbers of older patients with end-stage kidney disease? *Expert review of pharmacoeconomics & outcomes research*. 2010;10(6):661-666.

10. Farrington K, Covic A, Nistor I, et al. Clinical practice guideline on management of older patients with chronic kidney disease stage 3b or higher (eGFR< 45 mL/min/1.73 m2): A summary document from the European renal best practice group. *Nephrology Dialysis Transplantation*. 2017;32(1):9-16.

11. Graham-Brown M, March DS, Churchward DR, et al. Design and methods of CYCLE-HD: Improving cardiovascular health in patients with end stage renal disease using a structured programme of exercise: A randomised control trial. *BMC nephrology*. 2016;17(1):69.

12. Charmaz K. Constructing grounded theory (introducing qualitative methods series). 2nd edn. London: Sage Publications 2014.

13. Nixon AC, Bampouras TM, Pendleton N, et al. Diagnostic accuracy of frailty screening methods in advanced chronic kidney disease. *Nephron*. 2019;141(3):147-155.

14. Hoffmann TC, Glasziou PP, Boutron I, et al. Better reporting of interventions: Template for intervention description and replication (TIDieR) checklist and guide. *BMJ*. 2014;348:g1687.

15. Borg GA. Psychophysical bases of perceived exertion. *Med Sci Sports Exerc*. 1982;14(5):377-381.

16. Sim J, Lewis M. The size of a pilot study for a clinical trial should be calculated in relation to considerations of precision and efficiency. *J Clin Epidemiol*. 2012;65(3):301-308.

For peer review only

For peer review only - http://bmjopen.bmj.com/site/about/guidelines.xhtml

17. Young HM, Goodliffe S, Madhani M, et al. Co-producing progression criteria for feasibility studies: A partnership between patient contributors, clinicians and researchers. *International journal of environmental research and public health*. 2019;16(19):3756.

18. Avery KN, Williamson PR, Gamble C, et al. Informing efficient randomised controlled trials: Exploration of challenges in developing progression criteria for internal pilot studies. *BMJ open*. 2017;7(2):e013537.

19. Beddhu S, Bruns FJ, Saul M, et al. A simple comorbidity scale predicts clinical outcomes and costs in dialysis patients. *Am J Med*. 2000;108(8):609-613.

20. Lamb SE, Jørstad-Stein EC, Hauer K, et al. Development of a common outcome data set for fall injury prevention trials: The prevention of falls network europe consensus. *J Am Geriatr Soc*. 2005;53(9):1618-1622.

21. Young HML, Orme MW, Song Y, et al. Standardising the measurement of physical activity in people receiving haemodialysis: Considerations for research and practice. *BMC nephrology*. 2019;20(1):450.

22. Eldridge SM, Chan CL, Campbell MJ, et al. CONSORT 2010 statement: Extension to randomised pilot and feasibility trials. *Pilot and feasibility studies*. 2016;2(1):64.

23. Richards DA, Bazeley P, Borglin G, et al. Integrating quantitative and qualitative data and findings when undertaking randomised controlled trials. *BMJ open*. 2019;9(11).

24. Witham MD, Stott DJ. Conducting and reporting trials for older people. *Age Ageing*. 2017;46(6):889-894.

For peer review only

25. SONG initiative. Standardised outcomes in nephrology - haemodialysis. http://songinitiative.org/projects/song-hd/. Updated 2017. Accessed July, 2019.

26. Akpan A, Roberts C, Bandeen-Roche K, et al. Standard set of health outcome measures for older persons. *BMC geriatrics*. 2018;18(1):36.

27. Matsufuji S, Shoji T, Yano Y, et al. Effect of chair stand exercise on activity of daily living: A randomized controlled trial in hemodialysis patients. *Journal of Renal Nutrition*. 2015;25(1):17-24.

28. Yates T, Gray LJ, Henson J, et al. Impact of depression and anxiety on change to physical activity following a pragmatic diabetes prevention program within primary care: Pooled analysis from two randomized controlled trials. *Diabetes Care*. 2019;42(10):1847-1853.

29. Zatloukal J, Houchen-Wolloff L, Ward S, et al. Clinical significance for ESWT in COPD after a course of pulmonary rehabilitation. *Chro Respiratory Disease.*2015;46.1479973119853828

30. Carvalho LP, Kergoat M, Bolduc A, et al. A systematic approach for prescribing posthospitalization home-based physical activity for mobility in older adults: The PATH study. *Journal of the American Medical Directors Association*. 2019;20(10):1287-1293.

31. Department of Health. Physical activity guidelines for older adults. https://www.nhs.uk/live-well/exercise/physical-activity-guidelines-older-adults/. Updated 2019. Accessed April, 2020.

32. Baggetta R, D'Arrigo G, Torino C, et al. Effect of a home based, low intensity, physical exercise program in older adults dialysis patients: A secondary analysis of the EXCITE trial. *BMC geriatrics*. 2018;18(1):248.

For peer review only - http://bmjopen.bmj.com/site/about/guidelines.xhtml

33. Bohm C, Stewart K, Onyskie-Marcus J, Esliger D,et al. Effects of intradialytic cycling compared with pedometry on physical function in chronic outpatient hemodialysis: A prospective randomized trial. *Nephrology Dialysis Transplantation*. 2014;29(10):1947-1955.

34. Sheshadri A, Kittiskulnam P, Lazar AA, et al. A walking intervention to increase weekly steps in dialysis patients: A pilot randomized controlled trial. *American Journal of Kidney Diseases*. 2020; 75(4):488-496

35. de Labra C, Guimaraes-Pinheiro C, Maseda A, et al. Effects of physical exercise interventions in frail older adults: A systematic review of randomized controlled trials. *BMC geriatrics*. 2015;15(1):1.

36. Tawney KW, Tawney PJ, Hladik G, et al. The life readiness program: A physical rehabilitation program for patients on hemodialysis. *American journal of kidney diseases*. 2000;36(3):581-591.

37. Bogataj Š, Pajek J, Ponikvar JB, et al. Kinesiologist-guided functional exercise in addition to intradialytic cycling program in end-stage kidney disease patients: A randomised controlled trial. *Scientific reports*. 2020;10(1):1-10.

38. Hofmeyer MR, Alexander NB, Nyquist LV, et al. Floor-Rise strategy training in older adults. *J Am Geriatr Soc*. 2002;50(10):1702-1706.

39. Skelton DA, McLaughlin AW. Training functional ability in old age. *Physiotherapy*. 1996;82(3):159-167.

40. Giné-Garriga M, Roqué-Fíguls M, Coll-Planas L, et al. Physical exercise interventions for improving performance-based measures of physical function in community-dwelling, frail

For peer review only

older adults: A systematic review and meta-analysis. *Arch Phys Med Rehabil*. 2014;95(4):753-769. e3.

41. Liu C, Shiroy DM, Jones LY, et al. Systematic review of functional training on muscle strength, physical functioning, and activities of daily living in older adults. *European review of aging and physical activity*. 2014;11(2):95.

42. Clarke AL Jhamb M, Bennett P. Barriers and facilitators for engagement and implementation of exercise in end-stage kidney disease: Future theory-based interventions using the behavior change wheel. *Seminars in Dialysis*. 2019:1-12.

43. Michie S, Atkins L, West R. The behaviour change wheel. *A guide to designing interventions.1st ed.Great Britain: Silverback Publishing*. 2014:1003-1010.

44. Wasmann KA, Wijsman P, van Dieren S, et al. Partially randomised patient preference trials as an alternative design to randomised controlled trials: Systematic review and meta-analyses. *BMJ open*. 2019;9(10):e031151.

45. Craig P, Dieppe P, Macintyre S, et al. Developing and evaluating complex interventions: The new medical research council guidance. *BMJ*. 2008;337:a1655.

46. Tracy SJ. Qualitative quality: Eight “big-tent” criteria for excellent qualitative research. *Qualitative inquiry*. 2010;16(10):837-851.

LEGENDS TO FIGURES

Figure 1.CONSORT

For peer review only - http://bmjopen.bmj.com/site/about/guidelines.xhtml

Figure 2. The core components of an acceptable exercise programme for people living with frailty and receiving haemodialysis.

SUPPLEMENTARY MATERIAL

Supplementary material 1. Inclusion and exclusion criteria for the CYCLE-HD trial

Supplementary material 2. Summary of intervention characteristics, in line with TiDier guidance.

Supplementary material 3. *A priori* progression criteria based on the primary feasibility objectives.

Supplementary material 4. Patient-reported secondary outcome measures.

Supplementary material 5. Interview topic guide questions.

Supplementary material 6. Incidence of falls per person-year.

Supplementary material 7. Changes in exercise capacity and physical function after six months.

Supplementary material 8. Changes in physical activity (accelerometry data) after six months.

Supplementary material 9. Patient-reported outcomes measures after six months.

Supplementary material 10. Baseline demographic and clinical characteristics for the qualitative participants.

Supplementary material 11. Joint display of quantitative and qualitative results, with an overall assessment of mixed-methods inferences.

For peer review only - http://bmjopen.bmj.com/site/about/guidelines.xhtml

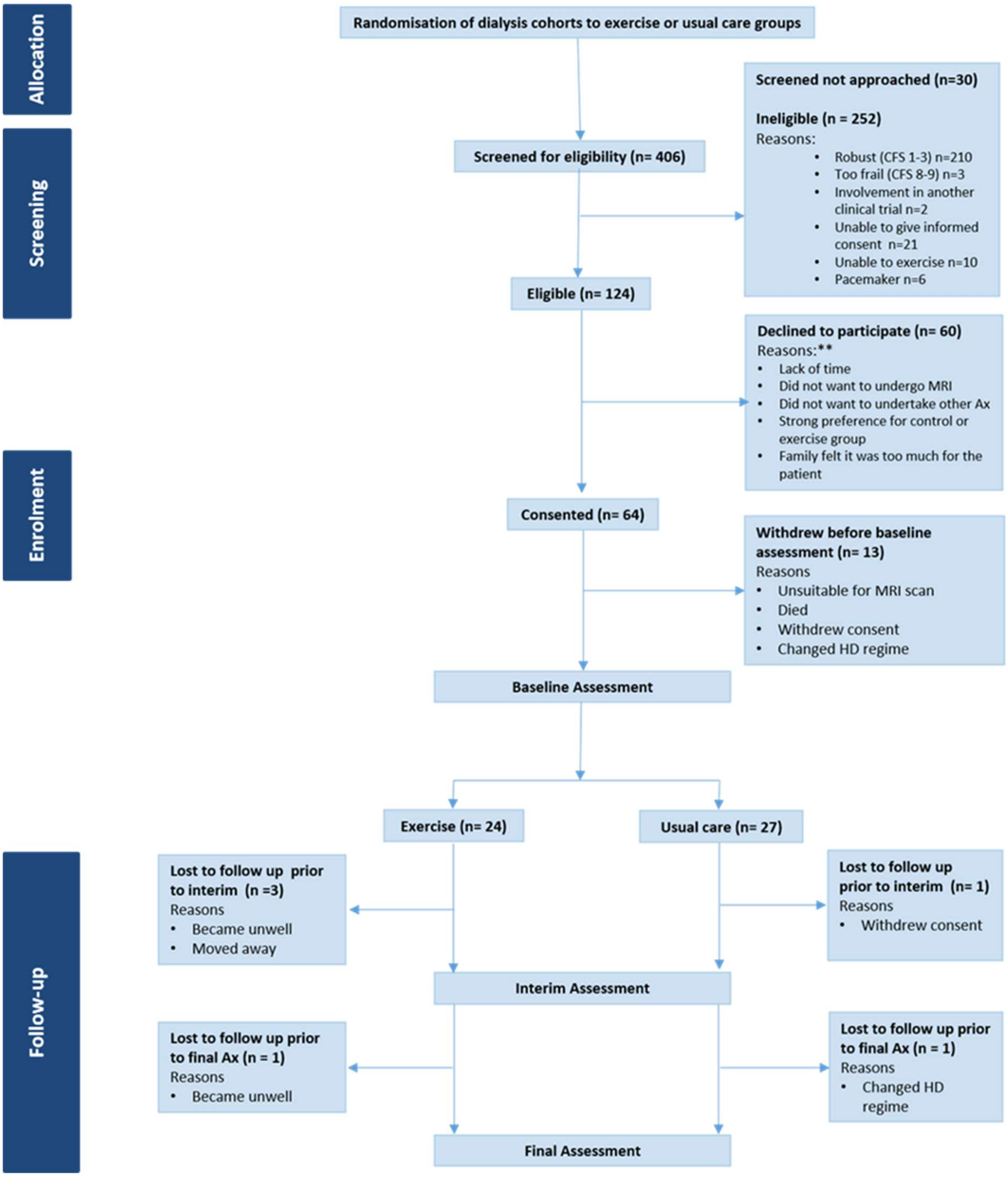

Abbreviations: Ax, assessment; CFS, Clinical Frailty Scale; HD, haemodialysis.

Supplementary material 1. Inclusion and exclusion criteria for the *CYCLE-HD* trial.

Inclusion criteria	Exclusion criteria
Prevalent HD patient (> three months)	Unable to participate in current exercise programme due to perceived physical or psychological barriers
Aged 18 years or older	Unable to undergo MRI scanning (metal implants, severe claustrophobia)
Able and willing to give informed consent	Unfit to undertake exercise according to the American College of Sports Medicine (ACSM) guidelines

For peer review only

Supplementary material 2. Summary of intervention characteristics, in line with TiDier guidance.

**Description of intervention.**		A structured, supervised cycling exercise intervention delivered during in-centre HD.
**Rationale.**		• IDC aerobic and low-level resistance training, IDC is associated with increased adherence and is most widely used within practice.
**What.**	Materials provided to participants or used to support intervention delivery.	• Cycling was delivered using the Moto Med Letto 2 (Medimotion Ltd).<br>• Materials: individualised exercise prescription and records of individual training bouts (duration (mins), intensity (RPE), resistance (gear), power output (watts) and energy expenditure (Kcal).<br>• General information on the benefits of exercise (posters and leaflets) available across all 3 HD centres.
	Materials used to train intervention providers.	Standardised progression and training protocol used by all providers.
**Who** (intervention providers).		• Qualified exercise professionals with experience of delivering exercise to renal patients.<br>• All providers were directly involved in the study, and not delivering the sessions as part of a clinical role.<br>• Roles included exercise provision, supervision, monitoring and progression.
**How** (mode of delivery).		One to one, face to face.
**Where** (location).		Three HD units across the East Midlands, UK.
**When and how much**	The frequency of delivery.	Thrice weekly during each dialysis session.
	Target intensity of each bout of exercise.	RPE 12-14 (moderate intensity), cadence 60-70 RPM.
	Target duration of each bout of exercise.	At least 30 minutes of continuous exercise.
	The total duration of delivery.	Six months, with a one-month run-in period to achieve the target exercise prescription.
**Tailoring.**		• The starting resistance (gear) based on the individual's tolerance.<br>• RPE used throughout to monitor and progress the exercise.<br>• Interval training was permitted.

Abbreviations: HD, haemodialysis; Kcals, kilocalories; RPE, rating of perceived exertion, RPM, revolutions per minute.

For peer review only

Supplementary material 3. A priori progression criteria based on the primary feasibility objectives.

**Eligibility**	**Stop**	Less than **20%** of all patients eligible
	**Go**	More than **50%** of all patients eligible
**Recruitment**	**Stop**	Less than **25%** of eligible patients recruited
	**Go**	More than **50%** of eligible patients recruited
**Exercise acceptability**	**Stop**	Less than **30%** adherence to the exercise sessions
	**Go**	More than **70%** adherence to the exercise sessions
**Outcome acceptability**	**Stop**	Less than **70%** outcome measure completion
	**Go**	More than **80%** outcome measure completion
**Loss to follow-up**	**Stop**	More than **40%** loss to follow-up
	**Go**	Less than **20%** loss to follow-up

For peer review only

Supplementary material 4. Patient-reported secondary outcome measures.

**Patient-reported secondary outcome**	**Construct measured**
12-item Short-Form Health Survey Version 2 (SF-12)	Generic health-related quality of life. Higher scores reflect better HRQoL.Scores are presented as a mental and physical component summary score.
Palliative care Outcomes Scale – Renal version (POS-R)	Renal specific measure of symptomology and symptom burden. A global symptom score was calculated by totalling all the scored items within the questionnaire. The mean number of symptoms, symptom severity was also calculated. Higher scores reflect greater symptom burden.
Hospital Anxiety and Depression Scale (HADS)	Emotional distress. A score of ≥14 indicates the presence of emotional distress in HD patients
The Exercise Self-Efficacy Scale (ESES)	Exercise confidence. Higher scores reflecting greater self-efficacy.
Dialysis Patient-Perceived Exercise Benefits and Barriers Scale (DPPEBBS)	HD patients' perceptions of benefits and barriers to exercise. Higher scores indicate a greater perception of the benefits of exercise over barriers.
The Dukes Activity Status Index (DASI)	Self-reported physical function. Higher scores indicate higher levels of physical function. The questionnaire was also used to estimate VO_2 peak.

For peer review only

Supplementary material 5. Interview topic guide question.

Diary

1. Can you tell me about how you have been using the diary?

2. If we asked patients to keep diaries like yours as part of a future study, what might help them?

3. [If applicable] I've had an opportunity to have a look through your diary. Could you tell me more about…?

Exercise intervention for frailty and falls

4. For some people exercising helps to prevent falls, make people more able and feel better. How do you feel about exercising?

5. Cycling during dialysis is thought to be a good way to exercise if you are on dialysis. Have you seen these bikes?

6. Programmes that are available for other people who fall include things like group exercise and education. What do you think about this?

7. These programmes usually take place at the hospital. What do you think about this?

8. Some people prefer to do their exercise at home. What do you think about this?

9. Where do you think a programme should be run?

10. How often do you think you would be able to exercise?

11. Would you want any support to help you exercise?

12. What might put you off exercising?

13. What questions might you have before you decide to take part or not?

14. If you did take part in some kind of exercise programme, what improvements would you most like to see?

Research

15. Have you ever been involved in research before? [Could tailor to involvement in CYCLE study (declined/ took part. If took part completed/dropped out) if patient unsure]

16. What do you think about the information you receive when deciding to take part in a research study?

For peer review only

17. Often researchers ask you to complete some assessments or tests to see if the thing they are studying is effective or not. What do you think would help patients to complete these assessments/ tests?

18. Sometimes people don't complete the research study, which may happen for several reasons [give examples as needed]. What do you think would help keep dialysis from dropping out of research studies?

19. What would you like to happen once you reach the end of the study?

For peer review only

Supplementary material 6. Incidence of falls per patient-year.

	Exercise	Usual care
**Falls**	5	11
**Patient-year**	36	40.5
**Incidence rate**	0.14	0.27

For peer review only

Supplementary material 7. Changes in exercise capacity and physical function after six months.

	Outcome		Usual Care	Exercise	Difference (95% CI)
**ISWT** (m)		n	16	15	36 (-12 to 84)
		Baseline	184 ± 130	237 ± 173	
		Final	158 ± 154	248 ± 192	
		Change	-26 ± 68	11 ± 63	
**ESWT** (secs)		n	14	15	181 (-92 to 453)
		Baseline	347 ± 384	401 ± 375	
		Final	193 ± 304	428 ± 423	
		Change	-153 ± 286	27 ± 413	
**STS60** (n)		n	17	15	0 (-5 to 4)
		Baseline	10 ± 12	13 ± 11	
		Final	10 ±13	13 ± 12	
		Change	0 ± 7	0 ± 6	
**SPPB**	Total score	n	17	15	0.5 (-0.7 to 2)
		Baseline	7 ± 3	9 ± 3	
		Final	6 ± 2	8 ± 3	
		Change	-1 ± 2	-0.5 ± 1	
	4m walk time (secs)	n	17	15	1 (-1 to 4)
		Baseline	7 ± 6	4± 1	
		Final	6 ± 4	5 ± 2	
		Change	1 ± 5	0 ± 1	
	Gait speed (m/s)	n	17	15	0.05 (-0.12 to 0.22)
		Baseline	0.74 ± 0.29	0.96 ± 0.28	
		Final	0.74 ± 0.28	0.91 ± 0.31	
		Change	0.00 ± 0.22	-0.05 ± 0.24	
	STS5 (secs)	n	9	10	5 (-4 to 15)
		Baseline	17 ± 7	16 ± 14	
		Final	23 ± 13	16 ± 10	
		Change	6 ± 11	0 ± 8.	

Abbreviations: CI, confidence interval; ESWT, Endurance Shuttle Walk Test; ISWT, Incremental Shuttle Walk Test; m/s, metres per second; Secs, seconds; SPPB, Short Physical Performance Battery; STS5, Sit to Stand Five Repetitions; STS60, Sit to Stand in Sixty Seconds.

For peer review only

Supplementary material 8. Changes in physical activity (accelerometry data) after six months.

	Type of day		Usual Care	Exercise	Difference (95% CI)
**Waking wear time** (mins)	HD	n	5	10	244 (16 to 473)
		Baseline	891 ± 202	818 ± 183	
		Final	749 ± 105	921 ± 171	
		Change	-142 ±166	103 ± 204	
	Non-HD	n	5	10	170 (-13 to 353)
		Baseline	893 ± 90	927 ± 216	
		Final	817 ± 134	1022 ± 165	
		Change	-75 ± 201	95 ± 129	
**Steps** (steps/day)	HD	n	5	10	859 (-825 to 2543)
		Baseline	2252 ± 4210	1373 ± 1080	
		Final	2464 ± 4783	2444 ± 1904	
		Change	211 ± 593	1070 ± 1665	
	Non-HD	n	5	10	888 (-84 to 1861)
		Baseline	3076 ± 5790	2387 ± 1696	
		Final	2645 ± 5284	2845 ± 2117	
		Change	-430 ± 603	458 ± 903	
**Sedentary** (mins/ day)	HD	n	5	10	28 (-284 to 340)
		Baseline	954 ±338	954 ± 203	
		Final	965 ± 208	992 ± 182	
		Change	10 ± 200	38 ± 287	
	Non-HD	n	5	10	124 (-205 to 454)
		Baseline	1022 ± 357	1103 ± 253	
		Final	912 ± 224	1117 ± 174	
		Change	-110 ± 298	14 ± 269	
**Light PA** (mins/day)	HD	n	5	10	91 (23 to -158)
		Baseline	125 ± 51	83 ± 42	
		Final	79 ± 39	127 ± 73	
		Change	-46 ± 45	44 ± 62	
	Non-HD	n	5	10	9 (-71 to 91)
		Baseline	145 ± 59	133 ± 50	
		Final	154 ± 99	151 ± 59	
		Change	9 ± 108	18 ± 44	
**Moderate PA** (mins/day)	HD	n	5	10	13 (-32 to 57)
		Baseline	83 ± 105	29 ± 33	
		Final	85 ± 123	43 ± 55	
		Change	1 ± 52	14 ± 29	
	Non-HD	n	5	10	20 (40 to -79)
		Baseline	79 ± 96	46 ± 61	
		Final	75 ± 112	62 ± 105	
		Change	-4 ± 40	16 ± 55	
**Vigorous PA** (mins/day)	HD	n	5	10	3 (-1 to 8)
		Baseline	4 ± 9	1 ± 1	
		Final	1 ± 2	1 ± 3	
		Change	-3 ± 7	0 ± 2	
	Non-HD	n	5	10	1 (0 to 2)
		Baseline	3 ± 0	1 ± 4	
		Final	2 ± 5	1 ± 4	
		Change	-1 ± 2	0 ± 0	

Abbreviations: CI, confidence interval; HD, haemodialysis; mins, minutes; PA, physical activity.

For peer review only

For peer review only - http://bmjopen.bmj.com/site/about/guidelines.xhtml

Supplementary material 9. Patient-reported outcomes measures after six months.

	Outcome		Usual Care	Exercise	Difference (95% CI)
**SF-12**	PCS	n	19	19	0 (-4 to 5)
		Baseline	35 ± 9	35 ± 10	
		Final	36 ± 10	36 ± 10	
		Change	1 ± 7	1 ± 7	
	MCS	n	19	19	4 (-3 to 10)
		Baseline	43 ± 15	45 ± 13	
		Final	46 ± 13	45 ± 13	
		Change	4 ± 7	0 ± 12	
**HADS**		n	20	17	0 (-3 to 4)
		Baseline	16 ± 10	15 ± 9	
		Final	14 ± 10	13 ± 9	
		Change	-2 ± 5	-2 ± 6	
**POS-R**	Global severity score	n	20	18	2 (-3 to 7)
		Baseline	19 ± 14	19 ± 14	
		Final	18 ± 14	20 ± 14	
		Change	1 ± 6	-1 ± 9	
	mean severity	n	20	18	0 (0 to 0)
		Baseline	2 ± 1	2 ± 1	
		Final	2 ± 1	2 ± 1	
		Change	0 ± 0	0 ± 0	
	mean number	n	22	16	0 (-1 to 2)
		Baseline	9 ± 4	10 ± 4	
		Final	9 ± 4	10 ± 5	
		Change	0 ± 4	0 ± 2	
**ESES**		n	19	16	0 (-1 to 1)
		Baseline	2 ± 2	2 ± 1	
		Final	2 ± 1	2 ± 1	
		Change	0 ± 1	0 ± 1	
**DPPEBBS**		n	19	15	3 (-4 to 11)
		Baseline	59 ± 10	59 ± 15	
		Final	61± 10	65 ± 7	
		Change	2 ± 7	6 ± 14	
**DASI**		n	20	18	4.93 (-0.94 to 10.80)
		Baseline	13.06 ± 12.85	20.29 ± 14.33	
		Final	17.29 ± 14.41	19.60 ± 14.59	
		Change	4.22 ± 9.72	-0.71 ± 7.92	

Abbreviations: CI, confidence interval; DASI, Duke Activity Status Index; DPPEBBS, Dialysis Patients Benefits and Barriers Scale; ESES, Exercise Self efficacy Scale; HADS, Hospital Anxiety and Depression Scale; MCS, mental component summary score; POS-R, Palliative Outcomes Scale Renal, PCS, physical component summary score; VAS, visual analogue scale.

For peer review only

Supplementary material 10. Baseline demographic and clinical characteristics for the qualitative participants.

		**N=25**
**Age** (years)		69±10
**Gender** n (%)	Female	13 (52%)
	Male	12 (48%)
**Ethnicity** n (%)	White background	13 (52%)
	Asian or Asian British	10 (40%)
	Caribbean	1 (4%)
	Not stated	1 (4%)
**Diagnosis**	Diabetic nephropathy	11 (44%)
	Aetiology uncertain	6 (24%)
	Chronic pyelonephritis	3 (12%)
	Atypical hemolytic uremic syndrome	1 (4%)
	FSGS	1 (4%)
	Henoch-Schönlein Purpura	1 (4%)
	Minimal change nephropathy	1 (4%)
	Polycystic kidney disease	1 (4%)
**CCI**		6±2
**Time on HD** (months)		43 (IQR 16-85)
**CFS** n (%)	Vulnerable	9 (36%)
	Mildly frail	5 (20%)
	Moderately frail	8 (32%)
	Severely frail	3 (12%)
**Number of falls in the last six months**		3 (IQR 2-4)
**Previous transplant** n (%)	No	21 (84%)
	Yes	4 (16%)
**Active on transplant list** n (%)	No	22 (88%)
	Yes	3 (12%

Abbreviations: CCI, Charlson comorbidity index; CFS, clinical frailty scale; FSGS, Focal segmental glomerulosclerosis; HD, haemodialysis.

For peer review only

Supplementary material 11. Joint display of quantitative and qualitative results, with an overall assessment of mixed-methods inferences.

	Progression criteria	Feasibility trial	Qualitative results	Mixed-methods inferences
**Eligibility**	**STOP** <20%<br>**GO** >50%<br>eligible.	31% patients eligible	No discussion. Patients not involved in screening process	Silence
**Recruitment**	**STOP** <25%<br>**GO** >50%<br>recruited.	52% eligible patients recruited.	- Frailer and female participants less likely to be approached despite eligibility and have more concerns about the suitability<br>- Perception that risks outweigh the potential benefit<br>- Recruitment processes could be improved	Complementary
**Retention**	**STOP** >40%<br>**GO** <20%<br>lost to follow-up.	12 % loss to follow-up. Reasons predominantly unavoidable (death, ill-health).	Loss to follow-up attributed to:<br>- Illness;<br>- length of trial;<br>- the reality of being in the study not meeting expectations.	Complementary
**Intervention**	**STOP** <30%<br>**GO** >70%<br>adherence over six-months.	74% adherence rate across the six-month exercise duration.	- IDC good use of time.<br>- Participants felt safe and felt well supported.<br>- IDC limited in scope.<br>- Participants described a range of other important components	Complementary
**Outcome**	**STOP** <70%<br>**GO** >80%<br>outcome measure completion.	Up to 89% of secondary outcome measure data missing<br>Collection of falls data challenging.	- Number of outcomes measured to be reduced.<br>- Outcome testing during HD or at home preferred.<br>- 52% agreed to complete a falls diary, 12% lost.<br>- STS60, ESWT and ISWT unsuitable<br>- Researcher support and family involvement may increase completion<br>- Outcomes measuring ADLs, participation and symptom prioritised	Complementary<br><br>Silence for PA monitoring.

Results from the feasibility trial are colour coded to depict whether they met the 'stop' (red), 'go' (green) or 'change (orange) progression criteria. Abbreviations: ADLs, activities of daily living; ESWT, Endurance Shuttle Walk Test; IDC, intradialytic exercise; ISWT, Incremental Shuttle Walk Test; PA, physical activity; STS60, sit to stand in sixty seconds.

For peer review only - http://bmjopen.bmj.com/site/about/guidelines.xhtml

CONSORT 2010 checklist of information to include when reporting a pilot or feasibility trial*

Section/Topic	Item No	Checklist item	Reported on page No
**Title and abstract**			
	1a	Identification as a pilot or feasibility randomised trial in the title	1
	1b	Structured summary of pilot trial design, methods, results, and conclusions (for specific guidance see CONSORT abstract extension for pilot trials)	3-4
**Introduction**			
Background and objectives	2a	Scientific background and explanation of rationale for future definitive trial, and reasons for randomised pilot trial	6
	2b	Specific objectives or research questions for pilot trial	6-7
**Methods**			
Trial design	3a	Description of pilot trial design (such as parallel, factorial) including allocation ratio	7-8
	3b	Important changes to methods after pilot trial commencement (such as eligibility criteria), with reasons	n/a
Participants	4a	Eligibility criteria for participants	7 and supplementary material 1
	4b	Settings and locations where the data were collected	7-8,10-11
	4c	How participants were identified and consented	8
Interventions	5	The interventions for each group with sufficient details to allow replication, including how and when they were actually administered	8 and supplementary material 2
Outcomes	6a	Completely defined prespecified assessments or measurements to address each pilot trial objective specified in 2b, including how and when they were assessed	9-11, supplementary materials 3,4 and 5
	6b	Any changes to pilot trial assessments or measurements after the pilot trial commenced, with reasons	n/a
	6c	If applicable, prespecified criteria used to judge whether, or how, to proceed with future definitive trial	9 and supplementary material 3
Sample size	7a	Rationale for numbers in the pilot trial	8-9

For peer review only

	7b	When applicable, explanation of any interim analyses and stopping guidelines	9
Randomisation:			
Sequence generation	8a	Method used to generate the random allocation sequence	8
	8b	Type of randomisation(s); details of any restriction (such as blocking and block size)	8
Allocation concealment mechanism	9	Mechanism used to implement the random allocation sequence (such as sequentially numbered containers), describing any steps taken to conceal the sequence until interventions were assigned	8
Implementation	10	Who generated the random allocation sequence, who enrolled participants, and who assigned participants to interventions	8
Blinding	11a	If done, who was blinded after assignment to interventions (for example, participants, care providers, those assessing outcomes) and how	9
	11b	If relevant, description of the similarity of interventions	n/a
Statistical methods	12	Methods used to address each pilot trial objective whether qualitative or quantitative	11
**Results**			
Participant flow (a diagram is strongly recommended)	13a	For each group, the numbers of participants who were approached and/or assessed for eligibility, randomly assigned, received intended treatment, and were assessed for each objective	12-13 figure 1
	13b	For each group, losses and exclusions after randomisation, together with reasons	12-13 figure 1
Recruitment	14a	Dates defining the periods of recruitment and follow-up	12
	14b	Why the pilot trial ended or was stopped	n/a
Baseline data	15	A table showing baseline demographic and clinical characteristics for each group	Table 1 (trial) supplementary material 10 (qualitative)
Numbers analysed	16	For each objective, number of participants (denominator) included in each analysis. If relevant, these numbers<br>should be by randomised group	Supplementary materials6-9 and page 15-17
Outcomes and estimation	17	For each objective, results including expressions of uncertainty (such as 95% confidence interval) for any estimates. If relevant, these results should be by randomised group	Supplementary materials 6-9 and page 15-17
Ancillary analyses	18	Results of any other analyses performed that could be used to inform the future definitive trial	17- 26
Harms	19	All important harms or unintended effects in each group (for specific guidance see CONSORT for harms)	P 17
	19a	If relevant, other important unintended consequences	n/a
**Discussion**			

For peer review only

Limitations	20	Pilot trial limitations, addressing sources of potential bias and remaining uncertainty about feasibility	30
Generalisability	21	Generalisability (applicability) of pilot trial methods and findings to future definitive trial and other studies	26-31
Interpretation	22	Interpretation consistent with pilot trial objectives and findings, balancing potential benefits and harms, and considering other relevant evidence	26-31
	22a	Implications for progression from pilot to future definitive trial, including any proposed amendments	26-31
**Other information**			
Registration	23	Registration number for pilot trial and name of trial registry	4 and 7
Protocol	24	Where the pilot trial protocol can be accessed, if available	7
Funding	25	Sources of funding and other support (such as supply of drugs), role of funders	31
	26	Ethical approval or approval by research review committee, confirmed with reference number	32

Citation: Eldridge SM, Chan CL, Campbell MJ, Bond CM, Hopewell S, Thabane L, et al. CONSORT 2010 statement: extension to randomised pilot and feasibility trials. BMJ. 2016;355.
*We strongly recommend reading this statement in conjunction with the CONSORT 2010, extension to randomised pilot and feasibility trials, Explanation and Elaboration for important clarifications on all the items. If relevant, we also recommend reading CONSORT extensions for cluster randomised trials, non-inferiority and equivalence trials, non-pharmacological treatments, herbal interventions, and pragmatic trials. Additional extensions are forthcoming: for those and for up to date references relevant to this checklist, see www.consort-statement.org.

For peer review only

BMJ Open

Exercise for people living with frailty and receiving haemodialysis: a mixed-methods randomised controlled feasibility study.

Journal:	*BMJ Open*
Manuscript ID	bmjopen-2020-041227.R1
Article Type:	Original research
Date Submitted by the Author:	24-Jul-2020
Complete List of Authors:	Young, Hannah; University of Leicester, Department of Respiratory Sciences; University Hospitals of Leicester NHS Trust, Department of Research and Innovation<br>March, Daniel; University of Leicester, Department of Cardiovascular Sciences<br>Highton, Patrick; University of Leicester, Department of Cardiovascular Sciences; Loughborough University, National Centre for Sport and Exercise Medicine<br>Graham-Brown, Matthew ; University of Leicester, Cardiovascular Sciences; Loughborough University, National Centre for Sport and Exercise Medicine<br>Churchward, Darren; University of Leicester, Department of Cardiovascular Sciences<br>Grantham, Charlotte; University of Leicester, Department of Cardiovascular Sciences<br>Goodliffe, Samantha; University of Leicester, Department of Health Sciences<br>Jones, William; University Hospitals of Leicester, Emergency Department, Leicester Royal Infirmary,<br>Cheung, Mei-Mei; University Hospitals of Leicester, Renal, Respiratory and Cardiovascular Clinical Management Group<br>Greenwood, Sharlene ; King's College Hospital, Department of Physiotherapy and Renal Medicine; King's College London, Department of Renal Medicine<br>Eborall, Helen; University of Leicester, Department of Health Sciences<br>Conroy, Simon; University of Leicester, Department of Health Sciences<br>Singh, Sally; Leicester Biomedical Research Unit, Centre for Exercise & Rehabilitation Science; University Hospitals of Leicester NHS Trust, Department of Respiratory Medicine, Glenfield Hospital<br>Smith, Alice; University of Leicester, Department of Health Sciences<br>Burton, James; University of Leicester, Department of Cardiovascular Sciences; Loughborough University, National Centre for Sport and Exercise Medicine
**Primary Subject Heading**:	Renal medicine
Secondary Subject Heading:	Rehabilitation medicine
Keywords:	Dialysis < NEPHROLOGY, End stage renal failure < NEPHROLOGY,

	REHABILITATION MEDICINE, QUALITATIVE RESEARCH, Clinical trials < THERAPEUTICS

SCHOLARONE™
Manuscripts

I, the Submitting Author has the right to grant and does grant on behalf of all authors of the Work (as defined in the below author licence), an exclusive licence and/or a non-exclusive licence for contributions from authors who are: i) UK Crown employees; ii) where BMJ has agreed a CC-BY licence shall apply, and/or iii) in accordance with the terms applicable for US Federal Government officers or employees acting as part of their official duties; on a worldwide, perpetual, irrevocable, royalty-free basis to BMJ Publishing Group Ltd ("BMJ") its licensees and where the relevant Journal is co-owned by BMJ to the co-owners of the Journal, to publish the Work in this journal and any other BMJ products and to exploit all rights, as set out in our licence.

The Submitting Author accepts and understands that any supply made under these terms is made by BMJ to the Submitting Author unless you are acting as an employee on behalf of your employer or a postgraduate student of an affiliated institution which is paying any applicable article publishing charge ("APC") for Open Access articles. Where the Submitting Author wishes to make the Work available on an Open Access basis (and intends to pay the relevant APC), the terms of reuse of such Open Access shall be governed by a Creative Commons licence – details of these licences and which Creative Commons licence will apply to this Work are set out in our licence referred to above.

Other than as permitted in any relevant BMJ Author's Self Archiving Policies, I confirm this Work has not been accepted for publication elsewhere, is not being considered for publication elsewhere and does not duplicate material already published. I confirm all authors consent to publication of this Work and authorise the granting of this licence.

For peer review only

Exercise for people living with frailty and receiving haemodialysis: a mixed-methods randomised controlled feasibility study.

Hannah ML Young (MSc)[1,2], Daniel S March (PhD)[3], Patrick J Highton (PhD)[3,4], Matthew PM Graham-Brown (PhD)[3,4], Darren C Churchward (MSc)[3], Charlotte Grantham (BSc)[3], S Goodliffe (MSc)[5], William Jones (BAHons)[6], Mei-Mei Cheung (BAHons)[7], Sharlene A Greenwood (PhD)[8], Helen Eborall (PhD)[9], Simon Conroy (PhD)[5], Sally J Singh (PhD)[10,11], Alice C Smith (PhD)[5], James O Burton (MD)[3,4].

[1]Department of Respiratory Sciences, University of Leicester, Leicester, UK.

[2]Department of Research and Innovation, University Hospitals of Leicester, UK

[3]Cardiovascular Sciences, University of Leicester, UK.

[4]National Centre for Sport and Exercise Medicine, Loughborough University, UK.

[5]Department of Health Sciences, University of Leicester, UK.

[6] Emergency Department, Leicester Royal Infirmary, University Hospitals of Leicester, UK.

[7] Renal, Respiratory and Cardiovascular Clinical Management Group, University Hospitals of Leicester, UK.

[8]Department of Physiotherapy and Renal Medicine, King's College Hospital and Department of Renal Medicine, King's College London, UK.

[9]Social Science Applied to Healthcare Improvement Research (SAPPHIRE) Group, Department of Health Sciences, University of Leicester, UK.

For peer review only - http://bmjopen.bmj.com/site/about/guidelines.xhtml

[10]Centre for Exercise & Rehabilitation Science, Leicester Biomedical Research Unit, Glenfield Hospital, UK.

[11]Department of Respiratory Medicine, Glenfield Hospital, University Hospitals of Leicester,UK.

Corresponding author:

Hannah Young

Leicester Kidney Lifestyle Team

Academic Unit

Leicester General Hospital

Gwendolen Road

Leicester

UK

LE4 5PW

0116 258 4346

hmly1@le.ac.uk

Running title: Exercise for frail haemodialysis patients

Abstract word count: 268

Word count: 4300

For peer review only - http://bmjopen.bmj.com/site/about/guidelines.xhtml

ABSTRACT

Objectives

Frailty is highly prevalent in haemodialysis (HD) patients, leading to poor outcomes. This study aimed to determine whether a Randomised Controlled Trial (RCT) of intradialytic exercise is feasible for frail HD patients, and explore how the intervention may be tailored to their needs.

Design

Mixed-methods feasibility.

Setting & participants

Prevalent adult HD patients of the *CYCLE-HD* trial with a Clinical Frailty Scale Score of 4-7 (vulnerable to severely frail) were eligible for the feasibility study.

Interventions

Participants in the exercise group undertook six-months of thrice-weekly, progressive, moderate intensity intradialytic cycling (IDC).

Outcomes

Primary outcomes were related to feasibility. Secondary outcomes were falls incidence, exercise capacity, physical function, physical activity and patient-reported outcomes (PROMS) at baseline and six months. Acceptability of trial procedures and the intervention were explored via diaries and interviews with n=25 frail HD patients who both participated in (n=13, 52%), and declined (n=12, 48%), the trial.

Results

For peer review only - http://bmjopen.bmj.com/site/about/guidelines.xhtml

124 (30%) patients were eligible, 64 (52%) consented and 51 (80%) completed a baseline assessment. N=24 (71% male; 59 ± 13 years) dialysed during shifts randomly assigned to exercise and n=27 (81% male; 65 ± 11 years) assigned to usual care. N=6 (12%) were lost to follow-up. The exercise group completed 74% of sessions. 27 to 89% of secondary outcome data were missing. Frail HD patients outlined several ways to enhance trial procedures. Maintaining ability to undertake activities of daily living and social participation were outcomes of primary importance. Participants desired a varied exercise programme.

Conclusions

A definitive RCT is feasible, however a comprehensive exercise programme may be more efficacious than IDC in this population.

Trial Registration

ISRCTN11299707; ISRCTN12840463

Keywords: feasibility; frailty; exercise; haemodialysis; mixed-methods.

For peer review only - http://bmjopen.bmj.com/site/about/guidelines.xhtml

STRENGTHS AND LIMITATIONS OF THIS STUDY

- To our knowledge, this is the first study to evaluate the feasibility of an exercise intervention for people living with frailty and receiving haemodialysis (HD).
- The Clinical Frailty Scale, a frailty risk-stratification measure which has been validated in an HD population, was used to identify eligible participants.
- This study is also the first to explore how trial procedures and exercise programmes should be specifically tailored to the needs of people living with frailty and receiving HD, from their own perspectives.
- Multiple qualitative methods (interviews and diaries) were used to explore participants perceptions, providing a form of triangulation which strengthens the conclusions made.
- Due to the nature of the intervention and resource limitations, we could not blind intervention providers, outcome assessors or study participants to group allocation.

For peer review only

INTRODUCTION

Frailty, "a multidimensional syndrome of decreased physiological reserve leading to increased vulnerability to minor health stressors", is highly prevalent within the haemodialysis (HD) population.[1,2] Increasing frailty is associated with worsening outcomes, including mortality, hospitalisation, falls, reduced Health-Related Quality of Life (HRQoL), psychological well-being, physical function, ability to undertake activities of daily living (ADLs) and increased symptom burden.[3-5]

Despite this, frailty is not static and evidence suggests that some factors associated with frailty are amenable to change.[6] Whilst the possible mediating role of exercise has been discussed, to our knowledge no original studies have examined the feasibility or effectiveness of an exercise programme for people living with frailty and receiving HD.[7] To date, exercise interventions for HD patients have focused upon intradialytic exercise, most commonly delivered by means of a cycle ergometer (intradialytic cycling, IDC), yet little is known about whether this is the most appropriate training stimulus for frail HD patients.[8] In addition, HD treatment can be poorly tolerated by frail patients and therefore IDC may represent an additional stressor to which these patients are particularly vulnerable.[9] European renal best practice guidance highlights a need for studies which identify how exercise programmes should be more specifically tailored to the needs of frail CKD patients[10], yet to date, there has also been no exploration of the needs, barriers and facilitators to exercise from the perspectives of people living with frailty and receiving HD themselves.

The aim of this study was to determine the feasibility of conducting an RCT investigating the effects of IDC for HD patients living with frailty by: (i) estimating rates of eligibility,

For peer review only - http://bmjopen.bmj.com/site/about/guidelines.xhtml

recruitment, retention, exercise adherence and outcome acceptability; and exploring (ii) the potential benefits of IDC across a range of secondary outcomes; and (iii) the perceptions of frail HD patients in relation to participating in clinical research, IDC and a tailored exercise intervention.

METHODS

Design

A mixed-methods, prospective, randomised feasibility study was conducted alongside concurrent qualitative diaries and interviews (Trial Registration numbers ISRCTN11299707; ISRCTN12840463). The feasibility study was a secondary analysis of the *CYCLE-HD* trial, whose aims and methods are reported elsewhere.[11] The qualitative component was underpinned by a constructivist Grounded Theory approach.[12] All participants provided written informed consent.

Participants

Prevalent adult (over 18 years) HD patients were recruited from three centres within the UK East Midlands Renal Network. In addition to the inclusion and exclusion criteria for the *CYCLE-HD* trial (supplementary material 1), the Clinical Frailty Scale (CFS), a risk stratification tool, was used to identify vulnerable to severely frail participants (CFS score 4-7).[13] The CFS has good predictive abilities in an HD population, good construct validity when compared with the Frailty Index, is less burdensome that the Frailty Phenotype, and has been validated in an HD population.[13-15]

For peer review only - http://bmjopen.bmj.com/site/about/guidelines.xhtml

The inclusion and exclusion criteria for the qualitative component mirrored the feasibility study and both those involved in the trial, and those who were eligible but declined to participate, were eligible.

Randomisation

HD cohorts were randomised prior to screening, based on a computer-generated randomisation algorithm held by the Robertson Centre for Biostatistics at the University of Glasgow.

Recruitment

Patients were screened for eligibility by their supervising nephrologist. Suitable patients were approached during HD, and the study explained. For the qualitative component, participants who had been involved in the feasibility study were recruited following completion of, or withdrawal from, the trial to prevent contamination.

Exercise intervention

Supplementary material 2 outlines the exercise intervention in line with TIDieR guidance.[16] Briefly, following a one-month run-in, participants in the exercise group undertook thrice-weekly supervised, moderate-intensity (Rating of Perceived Exertion, RPE 12-14) IDC (MOTOmed Letto2, Reck, Germany), for six months.[17] Cycling resistance was progressively increased to maintain RPE in response to exercise adaptation. Both arms continued with usual care HD as described elsewhere.[11]

For peer review only - http://bmjopen.bmj.com/site/about/guidelines.xhtml

Sample size

Determinations of sample size from a power calculation around a primary outcome are not relevant to a feasibility study and sample sizes of 24-50 are considered sufficient.[18] For the qualitative component maximum variation sampling was initially used to ensure diversity in frailty status and level of trial participation.[12] As understanding was gained from preliminary analyses, theoretical sampling was used to further recruit participants.[12] A maximum of 30 interviews were planned, but data collection ceased at the point where theoretical categories were saturated and no longer generated new insight (n=25).

Primary outcome measures

The primary feasibility outcomes are presented in supplementary material 3. Judgement regarding feasibility was based upon a set of *a priori* progression criteria. For each criterion, the development of 'stop'(indicating when there are issues with the trial that cannot be resolved) and 'go' thresholds (when there are no issues that may impede the success of a trial) were co-produced by patients, clinicians and researchers.[19,20] Results falling between these thresholds indicated that adaptation to trial procedures may render a definitive RCT viable.[20]

Baseline demographic and clinical variables

Demographic and clinical characteristics were gathered from participants' medical notes. The Charlson Comorbidity Index (CCI) was used to estimate the burden of comorbid disease.[21]

For peer review only - http://bmjopen.bmj.com/site/about/guidelines.xhtml

Secondary outcome measures

Multiple secondary outcomes were used to determine the potential effects of IDC and most appropriate primary endpoint for a future RCT. Outcome assessors were not blinded to group allocation.

Information on the number of falls, defined as 'an unexpected event in which the participants come to rest on the ground, floor, or lower-level' which resulted in Emergency Department visits and hospital admissions were collected from baseline to one year following intervention completion from medical records and hospital episode statistics.[22]

Field tests of exercise capacity and physical function included the Incremental Shuttle Walk Test (ISWT), the Endurance Shuttle Walk Test (ESWT), the Short Physical Performance Battery (SPPB) and the Sit-to Stand in Sixty Seconds (STS60).[11] Physical activity (PA) was objectively measured using the SenseWear Armband (SWA) Pro 3 (BodyMedia, Inc., Pittsburgh PA, USA) for seven consecutive days, including HD. Established criteria were used to ensure representative data for average daily wear-time, steps per day, and time (minutes per day) spent in sedentary (defined as 0-1.5 METS), light (1.6-2.9 METS) moderate (3-6 METS) and vigorous (>6 METS) PA.[23] PROMs collected are outlined in supplementary material 4.[11] All outcomes were collected at baseline and six months.

Serious adverse events (SAEs) were recorded and assessed from baseline to six-months as outlined previously.[11]

For peer review only - http://bmjopen.bmj.com/site/about/guidelines.xhtml

Diaries and interviews

Participants first completed a prospective falls diary, recognised as the current ‘gold standard’ for falls data collection, for up to three months to examine the feasibility of this outcome measure within a future definitive RCT.[22] Semi-structured interviews then explored participants’ experiences of: (i) keeping a falls diary; (ii) participating in a trial; and (iii) their perceptions of IDC and a tailored exercise intervention.

Information to support diary collection and a topic guide for the interviews (supplementary material 5) was developed by HMLY, HE and a patient and public involvement group. Topics were tailored according to the level of involvement in the trial, and the content of diaries. Interviews were conducted during HD, in the participant’s home, or in the hospital by HMLY and lasted 20 to 120 minutes (mean 63 minutes). All were digitally audio-recorded and transcribed verbatim.

Data analysis

Sample characteristics are presented as mean ± standard deviation, median (IQR) or n (%), as appropriate. Descriptive statistics and confidence intervals were used to estimate feasibility outcomes.[24] The percentage of exercise sessions completed was used to establish the acceptability of IDC. Outcome acceptability was determined by quantifying the amount of missing data across secondary outcomes. No imputation was performed to account for missing data. No statistical testing relating to the efficacy of the exercise intervention was undertaken, although the potential benefits of exercise were estimated.[24] For falls, summary data, incident rate ratio (the ratio of the incidence rate in the exercise group divided by the incidence rate in the usual care group) and 95% confidence intervals were presented.

For peer review only - http://bmjopen.bmj.com/site/about/guidelines.xhtml

Statistical analyses were performed using SPSS 24 (IBM UK Ltd, UK) and Stata 16 (StataCorp LCC,USA).

Qualitative analysis was undertaken by HMLY and SG and informed by a constant comparative approach.[12] Transcripts were reviewed, then coded line by line, followed by focused, and then theoretical, coding.[12] NVivo11 software (QSR International Ltd, version 11, 2016) was used to facilitate data management. Finally, qualitative and quantitative results were merged in a 'joint display' to facilitate an overall assessment of feasibility.[25]

Patient and public involvement

The patient and public involvement (PPI) group for this study comprised patients of all ages, genders and ethnicities who were living with frailty and receiving HD, and their relatives. They agreed this study was an important priority for further investigation and particularly stressed the need to add the qualitative component. The PPI group were involved early in ethical approval stages and were actively engaged in writing lay summaries and providing patient perspectives on data collection procedures, ethical issues, and the study dissemination plans. They assisted in the preparation of study documentation, interview topic guides and diary keeping materials. During the study, members of the PPI group attended regular steering meetings and were involved in co-producing the progression criteria.

RESULTS

Feasibility study

For peer review only - http://bmjopen.bmj.com/site/about/guidelines.xhtml

Eligibility and recruitment

Screening and recruitment took place from March 2015 to 2018, with data collection completed by November 2018. Figure 1 outlines the trial CONSORT. Of the 406 patients screened in the *CYCLE-HD* trial, n=124 (30%, 95% CI 26.1% to 35.3%) were identified as vulnerable to severely frail and therefore eligible for the feasibility study. Sixty-four participants (52%, 95% CI 42.5% to 60.7%) consented. Reasons for declining were lack of time or family support and reluctance to undergo outcome testing, or to be randomised. Those who declined to participate had a median age of 73 (IQR 67-8) years. N=35 (58%) were female and n=27 (42%) male. Twenty-five (42%) were classified as vulnerable according to the CFS, n=17 (28%) were mildly frail, n=9 (15%) moderately frail and n=9 (15%) severely frail. Thirteen (20%, 95% CI 11.3% to 32.2%) participants withdrew prior to baseline assessment. N=51 (80%, 95% CI 67.8% to 88.7%) completed this assessment. Twenty-four (47%) participants received dialysis during shifts randomised to exercise and twenty-seven (53%) during shifts randomised to usual care.

[FIGURE ONE TRIAL CONSORT TO BE INSERTED HERE]

Participant characteristics

Table 1 displays the characteristics of the trial participants at baseline. Groups were well matched across most variables. A lower proportion of participants were female (23.5%) and severely frail (6%) overall.

For peer review only - http://bmjopen.bmj.com/site/about/guidelines.xhtml

Table 1. Baseline demographic and clinical characteristics of the trial participants.

		Usual care (n=27)	Exercise (n=24)	All (n=51)
Age (years)		65 ± 11	59 ± 13	63 ± 12
Sex (n, %)	Female	5 (19%)	7 (29%)	12 (23.5%)
Ethnicity (n, %)	White	12 (44%)	11 (41%)	23 (45%)
	Asian or Asian British	11 (41%)	11 (46%)	22 (43%)
	Caribbean	1 (4%)	0 (0%)	1 (2%)
	Other ethnic	1 (4%)	1 (4%)	2 (4%)
	Not stated	2 (7%)	1 (4%)	3 (6%)
Diagnosis (n, %)	Aetiology Uncertain	8 (29%)	7 (29%)	15 (29%)
	Diabetic Nephropathy	5 (19%)	7 (29%)	12 (23%)
	Glomerulonephritis	5 (19%)	3 (14%)	8 (16%)
	Renal Vascular Disease	3 (11%)	2 (8%)	5 (10%)
	Other diagnoses	4 (15%)	1 (4%)	5 (10%)
	Chronic Pyelonephritis	2 (7%)	1 (4%)	3 (6%)
	Polycystic Kidney Disease	0 (0%)	2 (8%)	2 (4%)
	Not recorded	0 (0%)	1 (4%)	1 (2%)
CCI		5 ± 2	5 ± 2	5 ± 2
Previous transplant (n, %)	No	21 (75%)	18 (75%)	39 (76.5%)
	Yes	6 (21%)	6 (25%)	12 (23.5%)
Time on HD (months)		17 (7-53)	13 (10-61)	16 (8-53)
BMI (kg/m^2)		27.38 ± 6.72	25.87 ± 5.28	26.67 ± 6.07
Total no. medications		12 ± 4	12 ± 4	12 ± 4
Clinical Information	Albumin (g/L)	35.4 ± 4.4	37.4 ± 4.3	36.4 ± 4.4
	Haemoglobin (g/L)	107 ± 12	112 ± 17	107 ± 15
Haemodialysis	URR (%)*	74 (70-80)	75 (58-79)	74 (71-79)
	SBP (mmHg)	143 ± 21	144 ± 21	144 ± 21
	DBP (mmHg)*	65 (62-78)	78 (69-86)	76 (62-81)
CFS (n, %)	4, Vulnerable	13 (48%)	10 (42%)	23 (45%)
	5, Mildly frail	5 (18.5%)	7 (29%)	12 (23.5%)
	6, Moderately frail	8 (30%)	5 (21%)	13 (25.5%)
	7, Severely frail	1 (3.5%)	2 (8%)	3 (6%)

*Values reported are mean and SD (±), except for *median and IQR. Abbreviations: BMI, body mass index; CCI, Charlson Comorbidity Index; CFS, Clinical Frailty Scale DBP, diastolic blood pressure; SBP, systolic blood pressure; URR, urea reduction ratio*

For peer review only - http://bmjopen.bmj.com/site/about/guidelines.xhtml

Retention

Six (12%, 95% CI 4.4% to 23.9%) participants were lost to follow-up: three participants withdrew due to ill-health, one moved away, one changed HD regime and one withdrew consent.

Exercise adherence

A mean of 61±17 exercise sessions were completed over the six-month intervention, representing an adherence rate of 74±20%. The most frequent reasons for missing an exercise session were declining (n= 175 out of 535 sessions omitted in total, 33%), feeling unwell (n= 116, 22%) and pain (n= 105, 20%). Table 2 summarises the mean amount of exercise achieved. On average, participants reached the prescribed level of exercise by six months, although n=18 (75%) were unable to achieve this by the end of the one-month run-in period.

Table 2 Mean (SD) exercise achieved per session over the six-month duration of the intervention.

Duration (mins)	35 ± 8
Speed (RPM)	63 ± 10
Intensity (RPE)	13 ± 1
Gear	9 ± 4
Distance (Miles)	7 ± 3
Power (Watts)	13 ± 6
Energy expenditure (Kcals)	64 ± 31

All data presented as mean and SD (±). Abbreviations: kcals, kilocalories, mins, minutes; RPE, rating of perceived exertion; RPM, revolutions per minute.

Outcome acceptability

For tests of exercise capacity (ISWT and ESWT); n=14 (27%) did not complete at least one test at baseline, n=30 (64%) at interim and n=26 (58%) at final. For tests of physical function; n=20 (39%) did not complete at least one test at baseline, n=33 (70%) at interim and n=30 (67%) at final. For PROMs; n=27 (53%) did not complete at least one questionnaire at

For peer review only - http://bmjopen.bmj.com/site/about/guidelines.xhtml

baseline, n=27 (57%) at interim and n=40 (89%) at final. For PA data; n=21 (41%) were missing at baseline, and n=26 (58%) were missing at the final assessment. Declining was the primary reason for non-completion for all outcomes across all time points.

Secondary outcomes

Summary falls data are presented in supplementary material 6. The crude falls incident rate ratio (IRR) was 1.95 (95% CI 0.63 to 7.18), suggestive of an almost two-fold increased incidence of falls within the usual care group.

Exercise capacity was maintained in the exercise group, but deteriorated in the usual care group, resulting in an overall difference of 36m (95% CI -12 to 84) in ISWT results and 181 seconds (95% CI -92 to 453) in EWST time. The time taken to complete the STS5 also increased in the usual care group (suggesting a deterioration in function), but was maintained in the exercise group, resulting in an overall difference of 5 seconds (95% CI -4 to 15) (supplementary material 7).

Step count increased in the exercise group resulting in an overall difference of 859 steps/day (95%CI -825 to 2543) on HD days and 888 steps/day (95%CI -84 to 1861) on non-HD days. Whilst sedentary time was increased in the exercise group on all days compared with the usual care group, this appeared to be offset by increases in light PA and moderate PA, and maintenance (albeit of low levels) of vigorous PA versus maintenance or deterioration across the same metrics in the usual care group (supplementary material 8). For PROMs, outcomes were largely unchanged, except for the DASI score, which appeared to deteriorate in the exercise group and increase in the usual care group, resulting in an overall difference in score

For peer review only - http://bmjopen.bmj.com/site/about/guidelines.xhtml

of 4.93 (95% CI -0.94 to 10.80) and the mental component summary score of the SF12 which improved in the usual care group, resulting in an overall difference in score of 4 (95% CI -3 to 10). Exercisers appeared to have a greater perception of the benefits of exercise compared with those in the control group (3, 95% CI -4 to 11) (supplementary material 9).

Serious adverse events

In total, n=13 (25%) experienced an SAE during the feasibility study, n=8 (33%) in the exercise group and n=5 (19%) in the usual care group. The most common reasons for SAEs were vascular access complications (n=3, 17%), stroke (n=3, 17%), acute coronary syndrome (n=2, 11%) and non-specific chest pain (n=2, 11%). All events were classed as serious as they resulted in hospitalisation. All events resolved, and none were directly related to the intervention or trial.

Qualitative findings

Thirty-seven patients were approached for the qualitative study. Twenty-six were recruited and one died prior to data collection. Thirteen had participated in the feasibility trial. Nine received dialysis during shifts randomised to exercise, and four randomised to usual care. Twelve participants had declined to take part in the feasibility trial. Full characteristics for the qualitative sample are provided in supplementary material 10.

In addition to categories relating to the feasibility outcomes, categories relating to both the delivery and the characteristics of a tailored exercise intervention were identified. These are presented alongside illustrative quotes within Tables 3, 4, 5 and 6 and Figure 2.

For peer review only - http://bmjopen.bmj.com/site/about/guidelines.xhtml

Feasibility and acceptability of a definitive trial

Eligibility and recruitment

Declining to participate was underpinned by a perception that the trial could worsen overall health, particularly amongst those who had not previously participated in research or had recently commenced HD. Female participants believed that exercise was predominantly for men and that they were already doing enough daily activity, whilst participants living with moderate to severe frailty viewed ageing as an inevitable decline unlikely to be influenced by exercise. Motivators included a sense of altruism, and the perception that participation could provide opportunities to improve individual outcomes; learn about their own health; and access better healthcare. Participants felt that recruitment could be enhanced by the effective use of non-verbal communication, rapport building, adaptation to study documentation and actively involving family members in the recruitment process, as family support was often a prerequisite to participation (Table 3).

Trial retention

The primary reasons for withdrawal were becoming unwell, the duration of the trial and the research not meeting participants expectations. Participants suggested that having a rapport and maintaining regular dialogue with the research team might help retain participants within a future trial (Table 3).

For peer review only - http://bmjopen.bmj.com/site/about/guidelines.xhtml

Table 3. Categories relating to trial eligibility, recruitment and retention with illustrative quotes.

For peer review only - http://bmjopen.bmj.com/site/about/guidelines.xhtml

For peer review only

The acceptability of IDC

IDC was generally perceived to be a safe and positive use of HD treatment time. However, IDC was described as limited in scope, and participants were uncertain of its impact, particularly upon mobility, symptoms and falls (Table 4).

Table 4. Categories relating to the acceptability of IDC outcome acceptability and illustrative quotes.

Outcome acceptability

As indicated by participant quotations in Table 5, the number of outcomes and follow-ups needed to be reduced and participants had a strong preference for outcomes that could be collected during HD treatment. Many found the ISWT and STS60 assessments too challenging. Participants were occasionally uncertain of the purpose of the questionnaires and many reported difficulty quantifying symptom severity or a desire to provide ‘anticipated’ responses.

Maintaining mobility, and the ability to undertake a range of ADLS and social roles were viewed as key outcomes for a future trial. Only thirteen (52%) participants in the qualitative study agreed to complete a falls diary and many reported they preferred falls information to be collected during HD treatment. The majority who had fallen rarely reported them to healthcare professionals, believing that they were an expected consequence of HD or having had experience of their concerns about falls being overlooked. Consequently, falls prevention was not viewed as a key outcome.

For peer review only

388 *Table 5. Categories relating to outcome acceptability and illustrative quotes.*

For peer review only

Perceptions of a tailored exercise programme

Delivery

There was no universally acceptable setting for exercise delivery (Table 6). Vulnerable and mildly frail participants (CFS 4-5) were particularly open to group-based exercise in the community or gym, which they felt would provide motivation through camaraderie with others. However, access barriers due to HD treatment, complex health needs, and lack of transport were common. Participants also described feeling self-conscious exercising amongst 'normal' people. Home-based exercise was preferred by those with moderate to severe frailty (CFS 6-7) due to easier access, greater flexibility and relevance to their daily activities. Despite this, concerns about lack of space and safety were highlighted by those who lived alone, whilst those with family were concerned about overburdening or injuring them by asking for support.

For peer review only - http://bmjopen.bmj.com/site/about/guidelines.xhtml

401 *Table 6. Participants perceptions of the facilitators and barriers to group and home-based exercise.*

For peer review only

For peer review only - http://bmjopen.bmj.com/site/about/guidelines.xhtml

Characteristics of a tailored exercise programme

Irrespective of the setting for delivery, participants identified several key features of a tailored exercise intervention which are summarised in Figure 2.

[FIGURE TWO. THE CORE COMPONENTS OF AN ACCEPTABLE EXERCISE PROGRAMME FOR PEOPLE LIVING WITH FRAILTY AND RECEIVING HAEMODIALYSIS TO BE INSERTED HERE]

Preparation

Participants lived with a range of debilitating symptoms, most frequently fatigue, pain and dyspnoea. Often daily activity alone was felt to be enough of a challenge. Common impacts of exercise (for example breathlessness whilst exercising) were interpreted as worsening symptoms or damage, and many participants were uncertain if exercise would be suitable or beneficial. They indicated that the reason for exercising needed to be sufficiently compelling. They wanted to know what to expect prior to exercising, and individualised goal setting was advocated to build motivation and appreciate improvements.

Content

Key components described were whole body resistance, aerobic and balance training. Many participants described being unable to get up once they had fallen and felt that practising this was also important. Routine physical activity was viewed as more purposeful than structured exercise ‘for the sake of it’ and participants spoke of their enjoyment of being outside and engaging in meaningful and physically active hobbies.

For peer review only - http://bmjopen.bmj.com/site/about/guidelines.xhtml

Structure

Supervision was viewed as essential to select, teach and progress exercises. Individual tailoring which considered the impact of disability, comorbidities and fluctuating symptoms was important, and a choice of exercises, for example swimming, dancing and yoga, was associated with increased enjoyment and engagement. Moderate to severely frail participants wanted the programme to be progressed in a supportive and collaborative manner. Those who were vulnerable or mildly frail wanted to be 'pushed' and progressed in a more assertive manner.

Having a companion (typically peers, family or friends) was viewed as helping to overcome access barriers and provide socialisation and mutual motivation. The sharing of experience was also seen as a powerful means of challenging preconceptions about exercise ability, although participants with moderate to severe frailty raised concerns about feeling embarrassed or 'judged' if they were less able.

Integrated mixed-methods analyses

The integrated qualitative and quantitative findings suggest that an RCT of IDC is feasible for frail HD patients following adaptation. However, IDC should not be the only intervention offered and the development of a multicomponent programme is warranted (Supplementary material 11).

DISCUSSION

For peer review only - http://bmjopen.bmj.com/site/about/guidelines.xhtml

These results suggest that an RCT of IDC is feasible for frail HD patients with adaptation to increase outcome acceptability and eligibility rates. Adherence to IDC was high and it was viewed as a safe and efficient use of HD treatment time. Secondary outcomes also suggest that, for HD patients with a CFS of 4-7, IDC may mitigate deterioration in exercise capacity, endurance and functional muscle strength and increase PA behaviour (steps/day). Despite this, participants described a preference for a multi-component programme that prepared them for exercise, offered variety, companionship and individualised supervision. No single preferred environment for the delivery of this intervention was identified, but appeared to be influenced by frailty grade and individual factors.

27% to 89% of secondary outcome measure data were missing, and, overall, this progression criterion was not achieved. Given that secondary measures are often insufficiently powered, reducing the number collected within a future trial may improve completion.[26] Falls were not of primary importance to participants, and aligns with the SONG-HD data which did not identify falls as a key outcome.[27] Our findings suggest that accurately capturing prospective falls data may be challenging due to under-reporting, and yet, retrospective falls data collection does not fully reflect the incidence and impact of falls, particularly those which do not require an ED visit or hospital admission. Given the high incidence of falls in this population, capturing falls data may be important in a future trial, and regular prospective recording of information relating to falls as a part of routine practice at the dialysis unit is recommended, in line with participant feedback.[5] This would provide both clinicians and researchers with higher quality data for use in both prospective and retrospective studies, and to inform clinical care.

For peer review only - http://bmjopen.bmj.com/site/about/guidelines.xhtml

Further exploration and validation of meaningful measures for HD patients living with frailty is also warranted. Some of the functional measures (the STS60 and ISWT) included were too challenging. In the absence of a core set of functional outcome measures for older people, or people receiving haemodialysis, we suggest that the SPPB may be most appropriate and feasible method of capturing information about mobility and function. Although challenges with ceiling effects have been identified, this measure had the lowest levels of non-completion within this study, and has demonstrated good test-retest reliability in HD patients and excellent validity and responsiveness to change following an intervention in older adults.[28,29]To date, more comprehensive measures of basic and instrumental ADL ability and participation have rarely been used in exercise studies. These outcomes were, however, highlighted as important within this study, and have also been included in guidelines and core outcomes sets for HD and older people, warranting their inclusion in future exercise studies relating to frail HD populations.[27,30,31]

The results of this study indicate that changes to eligibility criteria and screening are required. As only patient participants were interviewed, it was not possible to gain any insight on this aspect of feasibility from the qualitative component. Importantly, the challenges of identifying eligible participants do not appear to be unique to this study. Studies of older people living with frailty highlight that large numbers need to be screened to achieve a 50% recruitment rate, and a multicentre trial may be required.[26] Higher proportions older, female and more severly frail HD patients declined to participate whilst the qualitative data indicated this was due to negative perceptions relating to participation in both exercise and research. Such findings clearly have implications for the external validity of a future trial and the reach of the intervention at the point of implementation.[24]

For peer review only - http://bmjopen.bmj.com/site/about/guidelines.xhtml

To address this, this study suggests the recruitment strategies which utilise effective non-verbal communication skills to build rapport and explore participants' perceptions of the intervention and the research process, and subsequently provide balanced information about the study, may lead to more representative recruitment. A sense of equipoise may be preserved by emphasising altruism, access to potentially enhanced care, and an opportunity to learn about their health (which were all identified as motivators to participation), rather than the potential individual benefits of the intervention itself. Involving families and/or peer supporters who have experience of the study and intervention in the recruitment process, and introducing opportunities for participants to observe the exercise intervention, may also be beneficial. Ultimately the selection of these strategies will depend upon the resources available and the need to strike a balance between conducting a trial with high internal and external validity and going beyond what is pragmatically possible to engage patients in the intervention at the implementation phase.

This study suggests that IDC may reduce the incidence of falls resulting in ED visits and hospital admissions in frail HD patients potentially by attenuating a decline in exercise capacity, physical activity behaviour and function at levels shown to be clinically meaningful in other long-term conditions.[32,33] This indicates that preventing deterioration may be as valuable, and more attainable, as improving outcomes in a frail population. Despite this, frail participants experienced difficulties achieving the proposed level of exercise and maintaining motivation in the face of varying symptomology. Exercise programmes have a dose-response, and these factors may have reduced participants physical capability to exercise and achieve optimal benefit, despite the overall good level of adherence. Clinical decision support tools

For peer review only - http://bmjopen.bmj.com/site/about/guidelines.xhtml

have been used in other populations to rationalise exercise prescription, progression and amendment in the presence of varying symptomology, and a similar approach may be beneficial for frail HD patients.[34]

This study indicates that participants desire a multicomponent exercise programme, and require an intervention that addresses their particularly low levels of PA. Whilst step count and time spent in light and moderate PA increased following IDC, these were below PA recommendations for older people.[35] To date, PA interventions for HD patients have predominantly centred around walking, which may not be appropriate for those living with frailty.[36-39] This study suggests that functional training (task-orientated exercise which engages multiple muscle groups) and physical activity that focuses on 'doing more' of these usual tasks may be more acceptable and efficacious. To date, two studies have employed similar approaches with non-frail HD patients. One study demonstrated significant improvements in lower extremity performance and the other a non-significant improvement in physical function and maintenance of other SF-36 domains compared with the control group.[40,41] In older people without CKD who are living with frailty, functional training included as part of a multicomponent exercise programme is beneficial across a range of outcomes, including greater ability to rise from the floor following a fall.[39,42-45] A similar approach to exercise prescription may be warranted in a frail HD population.

Numerous barriers and facilitators to exercise were identified within this study, which have implications for the design of a programme. The use of theory is crucial in the development of effective interventions and the behaviour change wheel (BCW) is most frequently cited in the development of interventions in CKD[46]. Mapping the identified barriers and facilitators to

For peer review only - http://bmjopen.bmj.com/site/about/guidelines.xhtml

the BCW indicates that ameliorating symptom burden prior to exercise, individualised exercise counselling, and a collaborative, problem-solving approach to exercise education are most likely to encourage and sustain participation.[46,47] Devising ways in which peer and family involvement can be incorporated into the programme may also increase motivation and opportunity to exercise but should be carefully managed given the potential for negative comparison amongst the frailest patients.

A lack of preferred environment for intervention delivery may have implications for a definitive RCT. Exercise interventions require motivation, and limited engagement may negatively influence a trials external and internal validity. Ignoring patient preference is also out of step with clinical practice, where rehabilitation involves shared decision-making. Taken together, these factors have implications for determining treatment effects and future intervention implementation.[48] There is increasing recognition that novel trial designs may be indicated when evaluating complex interventions and a Partially Randomised Patient Preference Trial, where participants without preference are randomised whilst those with a preference receive their choice, would provide information on both the efficacy of the intervention and the influence of preference.[48,49]

Strengths and limitations

To our knowledge, this study is the first to examine the feasibility of an RCT of IDC for frail HD patients and to explore how trial procedures and exercise programmes should be specifically tailored to the needs of this group, from their own perspectives. Key strengths were the use of a validated frailty risk-stratification measure and multiple qualitative methods which provided a form of triangulation.[50] There were, however, challenges to recruiting

For peer review only - http://bmjopen.bmj.com/site/about/guidelines.xhtml

severely frail participants, and those from a more diverse range of black and minority ethnic groups, to both the trial and the qualitative study. Additionally, the views of clinicians and researchers were not explored. A future RCT should also blind outcome assessors to group allocation to reduce the potential for detection bias. Finally, this study is exploratory and therefore all secondary measures of exercise capacity, function and PROMS should be interpreted with caution, not least due to the high number of participants who did not complete the follow up tests.

Conclusion

In summary, this study suggests that a future definitive trial of IDC is feasible within a HD population with a CFS of 4-7 and paying particular attention in the design to those factors mentioned above may facilitate improved rates of eligibility and outcome completion. Outcomes focusing on independence and participation should be the primary outcomes of interest in a future trial. Whilst an exploratory analysis suggests some potential benefits to IDC, a tailored intervention comprising a comprehensive multi-component programme, symptom management, education and behaviour change is better suited to frail HD patients' needs.

ACKNOWLEDGEMENTS

The authors would like to thank the staff and patients at all units, Fresenius Medical Care and The Leicester Kidney Care Appeal. The authors also wish to thank Freya Tyrer for sharing her statistical expertise.

For peer review only - http://bmjopen.bmj.com/site/about/guidelines.xhtml

COMPETING INTERESTS

The authors have no conflicts of interest to declare.

FUNDING

The work was funded by the National Institute for Health Research (NIHR) Leicester Biomedical Research Centre, Collaboration for Leadership in Applied Health Research and Care East Midlands (CLAHRC EM) and partly funded by the Stoneygate Trust. H.M.L.Y. and J.O.B. are supported by grants from the NIHR (DRF-2016-09-015 and CS-2013-13-014). S.J.S. is supported by the Collaboration for Leadership in Applied Health Research and Care East Midlands. The views expressed in this publication are those of the authors and not necessarily those of the NHS, the NIHR or the Department of Health and Social Care. The funders had no role in the study design; collection, analysis, and interpretation of the data;

AUTHORS CONTRIBUTIONS

Literature search: HMLY; Research idea and study design: HMLY, JOB; participant recruitment: HMLY, DS, PH, DC, MPMGB, JOB, CG; data acquisition: HMLY, DS,PH,DC, MPMGB,CG WJ, MC; clinical governance: JOB; data analysis: HMLY, SG; statistical analysis: HMLY; supervision and mentorship: SC, HE, SG, SJS, ACS, JOB; manuscript preparation: HMLY; reviewed final manuscript: all. Each author contributed important intellectual content during manuscript drafting or revision and accepts accountability for the overall work by ensuring that questions pertaining to the accuracy or integrity of any portion of the work are appropriately investigated and resolved. All authors have read and approved the final version

For peer review only - http://bmjopen.bmj.com/site/about/guidelines.xhtml

ETHICAL APPROVAL

This study was approved by the East Midlands (Northampton; REC ref: 14/EM/1190) and South West (Bristol; REC ref: 17/SW/0048) NHS Research Ethics Committees for the trial and the qualitative component respectively

DATA SHARING STATEMENT

The datasets used and analysed during the current study are available from the corresponding author on reasonable request.

For peer review only

For peer review only - http://bmjopen.bmj.com/site/about/guidelines.xhtml

REFERENCES

1. Clegg A, Young J, Iliffe S, et al. Frailty in elderly people. *The Lancet*. 2013;381(9868):752-762.

2. Zhao Y, Liu Q, Ji J. The prevalence of frailty in patients on hemodialysis: A systematic review and meta-analysis. *Int Urol Nephrol*. 2019:1-6.

3. Iyasere OU, Brown EA, Johansson L, et al. Quality of life and physical function in older patients on dialysis: A comparison of assisted peritoneal dialysis with hemodialysis. *Clin J Am Soc Nephrol*. 2016;11(3):423-430.

4. Johansen KL, Chertow GM, Jin C, et al. Significance of frailty among dialysis patients. *Journal of the American Society of Nephrology*. 2007;18(11):2960-2967.

5. López-Soto PJ, De Giorgi A, Senno E, et al. Renal disease and accidental falls: A review of published evidence. *BMC nephrology*. 2015;16(1):176.

6. Johansen KL, Dalrymple LS, Delgado C, et al. Factors associated with frailty and its trajectory among patients on hemodialysis. *Clinical Journal of the American Society of Nephrology*. 2017;12(7):1100-1108.

7. Wang CJ, Johansen KL. Are dialysis patients too frail to exercise? Seminars in dialysis.2019;32(4) 1-6.

8. Young HM, March DS, Graham-Brown MP, et al. Effects of intradialytic cycling exercise on exercise capacity, quality of life, physical function and cardiovascular measures in adult haemodialysis patients: A systematic review and meta-analysis. *Nephrology Dialysis Transplantation*. 2018; 33(8),1436-1445.

For peer review only - http://bmjopen.bmj.com/site/about/guidelines.xhtml

9. Brown EA. Can quality of life be improved for the increasing numbers of older patients with end-stage kidney disease? *Expert review of pharmacoeconomics & outcomes research*. 2010;10(6):661-666.

10. Farrington K, Covic A, Nistor I, et al. Clinical practice guideline on management of older patients with chronic kidney disease stage 3b or higher (eGFR< 45 mL/min/1.73 m2): A summary document from the european renal best practice group. *Nephrology Dialysis Transplantation*. 2017;32(1):9-16.

11. Graham-Brown M, March DS, Churchward DR, et al. Design and methods of CYCLE-HD: Improving cardiovascular health in patients with end stage renal disease using a structured programme of exercise: A randomised control trial. *BMC nephrology*. 2016;17(1):69.

12. Charmaz K. Constructing grounded theory (introducing qualitative methods series). 2nd edn. London: Sage Publications 2014.

13. Nixon AC, Bampouras TM, Pendleton N, et al. Diagnostic accuracy of frailty screening methods in advanced chronic kidney disease. *Nephron*. 2019;141(3):147-155.

14. Rockwood K, Song X, MacKnight C, et al. A global clinical measure of fitness and frailty in elderly people. *CMAJ*. 2005;173(5):489-495.

15. Alfaadhel TA, Soroka SD, Kiberd BA, et al. Frailty and mortality in dialysis: Evaluation of a clinical frailty scale. *Clin J Am Soc Nephrol*. 2015;10(5):832-840.

16. Hoffmann TC, Glasziou PP, Boutron I, et al. Better reporting of interventions: Template for intervention description and replication (TIDieR) checklist and guide. *BMJ*. 2014;348:g1687.

For peer review only

For peer review only - http://bmjopen.bmj.com/site/about/guidelines.xhtml

17. Borg GA. Psychophysical bases of perceived exertion. *Med Sci Sports Exerc*. 1982;14(5):377-381.

18. Sim J, Lewis M. The size of a pilot study for a clinical trial should be calculated in relation to considerations of precision and efficiency. *J Clin Epidemiol*. 2012;65(3):301-308.

19. Young HML, Goodliffe S, Madhani M, et al. Co-producing progression criteria for feasibility studies: A partnership between patient contributors, clinicians and researchers. *International journal of environmental research and public health*. 2019;16(19):3756.

20. Avery KN, Williamson PR, Gamble C, et al. Informing efficient randomised controlled trials: Exploration of challenges in developing progression criteria for internal pilot studies. *BMJ open*. 2017;7(2):e013537.

21. Beddhu S, Bruns FJ, Saul M, et al. A simple comorbidity scale predicts clinical outcomes and costs in dialysis patients. *Am J Med*. 2000;108(8):609-613.

22. Lamb SE, Jørstad-Stein EC, Hauer K, et al. Development of a common outcome data set for fall injury prevention trials: The prevention of falls network europe consensus. *J Am Geriatr Soc*. 2005;53(9):1618-1622.

23. Young HML, Orme MW, Song Y, et al. Standardising the measurement of physical activity in people receiving haemodialysis: Considerations for research and practice. *BMC nephrology*. 2019;20(1):450.

24. Eldridge SM, Chan CL, Campbell MJ, et al. CONSORT 2010 statement: Extension to randomised pilot and feasibility trials. *Pilot and feasibility studies*. 2016;2(1):64.

For peer review only - http://bmjopen.bmj.com/site/about/guidelines.xhtml

25. Richards DA, Bazeley P, Borglin G, et al. Integrating quantitative and qualitative data and findings when undertaking randomised controlled trials. *BMJ open*. 2019;9(11).

26. Witham MD, Stott DJ. Conducting and reporting trials for older people. *Age Ageing*. 2017;46(6):889-894.

27. SONG initiative. Standardised outcomes in nephrology - haemodialysis. http://songinitiative.org/projects/song-hd/. Updated 2017. Accessed July, 2019.

28. Lamb SE, Keene DJ. Measuring physical capacity and performance in older people. *Best Practice & Research Clinical Rheumatology*. 2017;31(2):243-254.

29. de Villar LO, Martínez-Olmos FJ, Junqué-Jiménez A, et al. Test-retest reliability and minimal detectable change scores for the short physical performance battery, one-legged standing test and timed up and go test in patients undergoing hemodialysis. *PloS one*. 2018;13(8):e0201035.

30. Akpan A, Roberts C, Bandeen-Roche K, et al. Standard set of health outcome measures for older persons. *BMC geriatrics*. 2018;18(1):36.

31. Matsufuji S, Shoji T, Yano Y, et al. Effect of chair stand exercise on activity of daily living: A randomized controlled trial in hemodialysis patients. *Journal of Renal Nutrition*. 2015;25(1):17-24.

32. Yates T, Gray LJ, Henson J, et al. Impact of depression and anxiety on change to physical activity following a pragmatic diabetes prevention program within primary care: Pooled analysis from two randomized controlled trials. *Diabetes Care*. 2019;42(10):1847-1853.

For peer review only - http://bmjopen.bmj.com/site/about/guidelines.xhtml

33. Zatloukal J, Houchen-Wolloff L, Ward S, et al. Clinical significance for ESWT in COPD after a course of pulmonary rehabilitation. *Chro Respiratory Disease.*2015;46.1479973119853828

34. Carvalho LP, Kergoat M, Bolduc A, et al. A systematic approach for prescribing posthospitalization home-based physical activity for mobility in older adults: The PATH study. *Journal of the American Medical Directors Association*. 2019;20(10):1287-1293.

35. Department of Health. Physical activity guidelines for older adults. https://www.nhs.uk/live-well/exercise/physical-activity-guidelines-older-adults/. Updated 2019. Accessed April, 2020.

36. Baggetta R, D'Arrigo G, Torino C, et al. Effect of a home based, low intensity, physical exercise program in older adults dialysis patients: A secondary analysis of the EXCITE trial. *BMC geriatrics*. 2018;18(1):248.

37. Bohm C, Stewart K, Onyskie-Marcus J, Esliger D, et al. Effects of intradialytic cycling compared with pedometry on physical function in chronic outpatient hemodialysis: A prospective randomized trial. *Nephrology Dialysis Transplantation*. 2014;29(10):1947-1955.

38. Sheshadri A, Kittiskulnam P, Lazar AA, et al. A walking intervention to increase weekly steps in dialysis patients: A pilot randomized controlled trial. *American Journal of Kidney Diseases*. 2020; 75(4):488-496

39. de Labra C, Guimaraes-Pinheiro C, Maseda A, et al. Effects of physical exercise interventions in frail older adults: A systematic review of randomized controlled trials. *BMC geriatrics*. 2015;15(1):1.

For peer review only - http://bmjopen.bmj.com/site/about/guidelines.xhtml

727 40. Tawney KW, Tawney PJ, Hladik G, et al. The life readiness program: A physical
728 rehabilitation program for patients on hemodialysis. *American journal of kidney diseases*.
729 2000;36(3):581-591.

730 41. Bogataj Š, Pajek J, Ponikvar JB, et al. Kinesiologist-guided functional exercise in
731 addition to intradialytic cycling program in end-stage kidney disease patients: A randomised
732 controlled trial. *Scientific reports*. 2020;10(1):1-10.

733 42. Hofmeyer MR, Alexander NB, Nyquist LV, et al. Floor-Rise strategy training in older
734 adults. *J Am Geriatr Soc*. 2002;50(10):1702-1706.

735 43. Skelton DA, McLaughlin AW. Training functional ability in old age. *Physiotherapy*.
736 1996;82(3):159-167.

737 44. Giné-Garriga M, Roqué-Fíguls M, Coll-Planas L, et al. Physical exercise interventions for
738 improving performance-based measures of physical function in community-dwelling, frail
739 older adults: A systematic review and meta-analysis. *Arch Phys Med Rehabil*.
740 2014;95(4):753-769. e3.

741 45. Liu C, Shiroy DM, Jones LY, et al. Systematic review of functional training on muscle
742 strength, physical functioning, and activities of daily living in older adults. *European review*
743 *of aging and physical activity*. 2014;11(2):95.

744 46. Clarke AL Jhamb M Bennett P. Barriers and facilitators for engagement and
745 implementation of exercise in end-stage kidney disease: Future theory-based interventions
746 using the behavior change wheel. *Seminars in Dialysis*. 2019:1-12.

747 47. Michie S, Atkins L, West R. The behaviour change wheel. *A guide to designing*
748 *interventions.1st ed.Great Britain: Silverback Publishing*. 2014:1003-1010.

For peer review only - http://bmjopen.bmj.com/site/about/guidelines.xhtml

48. Wasmann KA, Wijsman P, van Dieren S, et al. Partially randomised patient preference trials as an alternative design to randomised controlled trials: Systematic review and meta-analyses. *BMJ open*. 2019;9(10):e031151.

49. Craig P, Dieppe P, Macintyre S, et al. Developing and evaluating complex interventions: The new medical research council guidance. *BMJ*. 2008;337:a1655.

50. Tracy SJ. Qualitative quality: Eight “big-tent” criteria for excellent qualitative research. *Qualitative inquiry*. 2010;16(10):837-851.

Legends to Figures

Figure 1.CONSORT

Figure 2. The core components of an acceptable exercise programme for people living with frailty and receiving haemodialysis.

SUPPLEMENTARY MATERIAL

Supplementary material 1. Inclusion and exclusion criteria for the CYCLE-HD trial

Supplementary material 2. Summary of intervention characteristics, in line with TiDier guidance.

Supplementary material 3. *A priori* progression criteria based on the primary feasibility objectives.

Supplementary material 4. Patient-reported secondary outcome measures.

Supplementary material 5. Interview topic guide questions.

For peer review only - http://bmjopen.bmj.com/site/about/guidelines.xhtml

Supplementary material 6. Falls summary data and incidence of falls per person years.

Supplementary material 7. Changes in exercise capacity and physical function after six months.

Supplementary material 8. Changes in physical activity (accelerometry data) after six months.

Supplementary material 9. Patient-reported outcomes measures after six months.

Supplementary material 10. Baseline demographic and clinical characteristics for the qualitative participants.

Supplementary material 11. Joint display of quantitative and qualitative results, with an overall assessment of mixed-methods inferences.

For peer review only

For peer review only - http://bmjopen.bmj.com/site/about/guidelines.xhtml

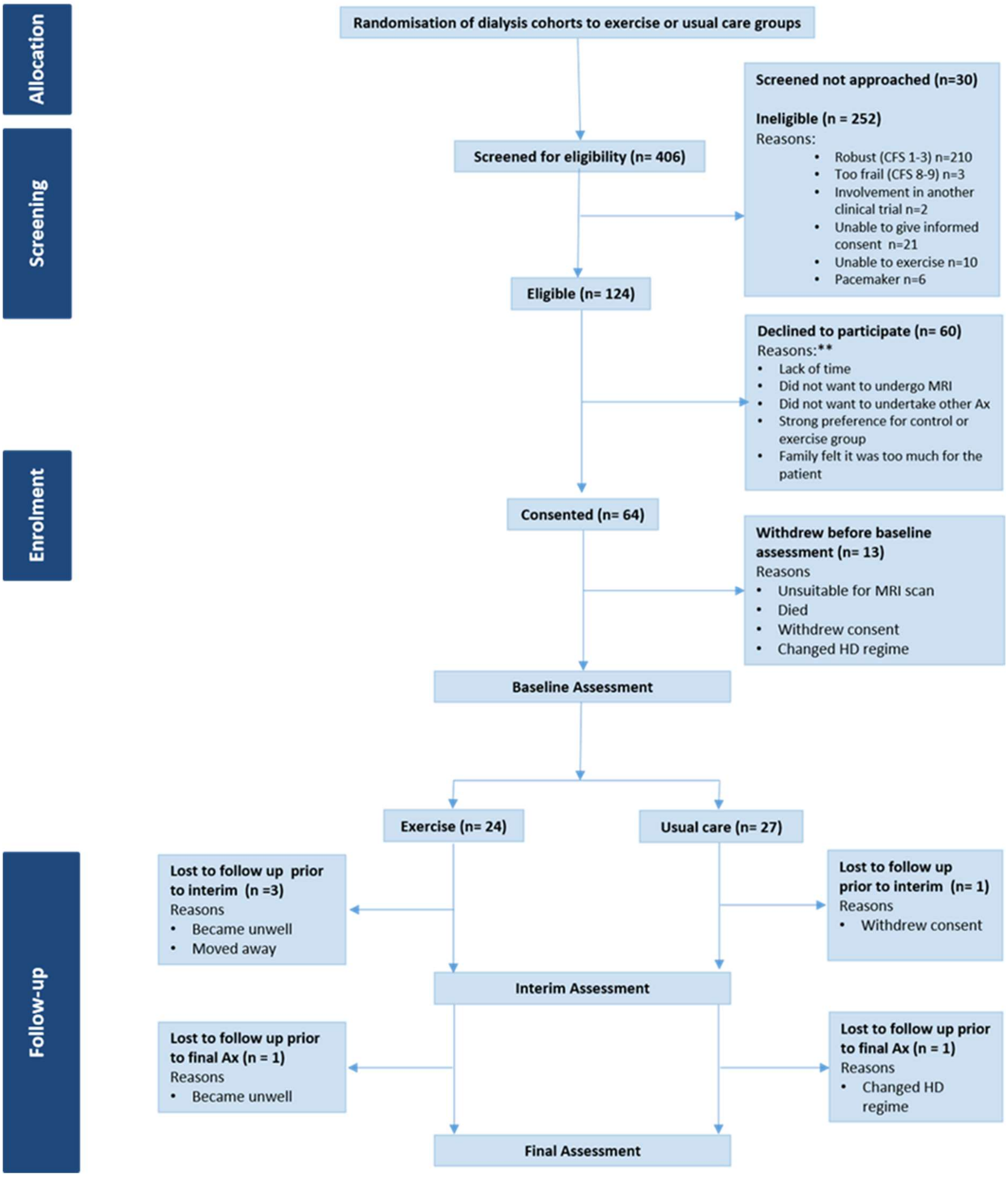

Abbreviations: Ax, assessment; CFS, Clinical Frailty Scale; HD, haemodialysis.

Supplementary material 1. Inclusion and exclusion criteria for the *CYCLE-HD* trial.

Inclusion criteria	Exclusion criteria
Prevalent HD patient (> three months)	Unable to participate in current exercise programme due to perceived physical or psychological barriers
Aged 18 years or older	Unable to undergo MRI scanning (metal implants, severe claustrophobia)
Able and willing to give informed consent	Unfit to undertake exercise according to the American College of Sports Medicine (ACSM) guidelines

For peer review only

Supplementary material 2. Summary of intervention characteristics, in line with TiDier guidance.

**Description of intervention.**		A structured, supervised cycling exercise intervention delivered during in-centre HD.
**Rationale.**		• Intradialytic cycling provides aerobic and low-level resistance training, is associated with increased adherence and is most widely used within practice.
**What.**	Materials provided to participants or used to support intervention delivery.	• Cycling was delivered using the Moto Med Letto 2 (Medimotion Ltd).<br>• Materials: individualised exercise prescription and records of individual training bouts (duration (mins), intensity (RPE), resistance (gear), power output (watts) and energy expenditure (Kcal).<br>• General information on the benefits of exercise (posters and leaflets) available across all 3 HD centres.
	Materials used to train intervention providers.	Standardised progression and training protocol used by all providers.
**Who** (intervention providers).		• Qualified exercise professionals with experience of delivering exercise to renal patients.<br>• All providers were directly involved in the study, and not delivering the sessions as part of a clinical role.<br>• Roles included exercise provision, supervision, monitoring and progression.
**How** (mode of delivery).		One to one, face to face.
**Where** (location).		Three HD units across the East Midlands, UK.
**When and how much**	The frequency of delivery.	Thrice weekly during each dialysis session.
	Target intensity of each bout of exercise.	RPE 12-14 (moderate intensity), cadence 60-70 RPM.
	Target duration of each bout of exercise.	At least 30 minutes of continuous exercise.
	The total duration of delivery.	Six months, with a one-month run-in period to achieve the target exercise prescription.
**Tailoring.**		• The starting resistance (gear) based on the individual's tolerance.<br>• RPE used throughout to monitor and progress the exercise.<br>• Interval training was permitted.

Abbreviations: HD, haemodialysis; Kcals, kilocalories; RPE, rating of perceived exertion, RPM, revolutions per minute.

For peer review only

Supplementary material 3. A priori progression criteria based on the primary feasibility objectives.

**Eligibility**	**Stop**	Less than **20%** of all patients eligible
	**Go**	More than **50%** of all patients eligible
**Recruitment**	**Stop**	Less than **25%** of eligible patients recruited
	**Go**	More than **50%** of eligible patients recruited
**Exercise acceptability**	**Stop**	Less than **30%** adherence to the exercise sessions
	**Go**	More than **70%** adherence to the exercise sessions
**Outcome acceptability**	**Stop**	Less than **70%** outcome measure completion
	**Go**	More than **80%** outcome measure completion
**Loss to follow-up**	**Stop**	More than **40%** loss to follow-up
	**Go**	Less than **20%** loss to follow-up

For peer review only

Supplementary material 4. Patient-reported secondary outcome measures.

**Patient-reported secondary outcome**	**Construct measured**
12-item Short-Form Health Survey Version 2 (SF-12)	Generic health-related quality of life. Higher scores reflect better HRQoL.Scores are presented as a mental and physical component summary score.
Palliative care Outcomes Scale – Renal version (POS-R)	Renal specific measure of symptomology and symptom burden. A global symptom score was calculated by totalling all the scored items within the questionnaire. The mean number of symptoms, symptom severity was also calculated. Higher scores reflect greater symptom burden.
Hospital Anxiety and Depression Scale (HADS)	Emotional distress. A score of ≥14 indicates the presence of emotional distress in HD patients
The Exercise Self-Efficacy Scale (ESES)	Exercise confidence. Higher scores reflecting greater self-efficacy.
Dialysis Patient-Perceived Exercise Benefits and Barriers Scale (DPPEBBS)	HD patients' perceptions of benefits and barriers to exercise. Higher scores indicate a greater perception of the benefits of exercise over barriers.
The Dukes Activity Status Index (DASI)	Self-reported physical function. Higher scores indicate higher levels of physical function. The questionnaire was also used to estimate VO_2 peak.

HD, haemodialysis; HRQoL, Health-related quality of life.

For peer review only

Supplementary material 5. Interview topic guide question.

Diary

1. Can you tell me about how you have been using the diary?

2. If we asked patients to keep diaries like yours as part of a future study, what might help them?

3. [If applicable] I've had an opportunity to have a look through your diary. Could you tell me more about…?

Exercise intervention for frailty and falls

4. For some people exercising helps to prevent falls, make people more able and feel better. How do you feel about exercising?

5. Cycling during dialysis is thought to be a good way to exercise if you are on dialysis. Have you seen these bikes?

6. Programmes that are available for other people who fall include things like group exercise and education. What do you think about this?

7. These programmes usually take place at the hospital. What do you think about this?

8. Some people prefer to do their exercise at home. What do you think about this?

9. Where do you think a programme should be run?

10. How often do you think you would be able to exercise?

11. Would you want any support to help you exercise?

12. What might put you off exercising?

13. What questions might you have before you decide to take part or not?

14. If you did take part in some kind of exercise programme, what improvements would you most like to see?

Research

15. Have you ever been involved in research before? [Could tailor to involvement in CYCLE study (declined/ took part. If took part completed/dropped out) if patient unsure]

16. What do you think about the information you receive when deciding to take part in a research study?

For peer review only

17. Often researchers ask you to complete some assessments or tests to see if the thing they are studying is effective or not. What do you think would help patients to complete these assessments/ tests?

18. Sometimes people don't complete the research study, which may happen for several reasons [give examples as needed]. What do you think would help keep dialysis from dropping out of research studies?

19. What would you like to happen once you reach the end of the study?

For peer review only

Supplementary material 6. Falls summary data and incidence of falls per person years.

	**Usual care (n=27)**	**Exercise (n=24)**
**Number of Falls**	11	5
**Number (% of group) of non-fallers**	19 (70)	20 (83)
**Number (% of group) fallers (≥ 1 fall)**	8 (30)	4 (17)
**Number (% of group) frequent fallers (≥2 falls)**	3 (11)	1 (4)
**Person years**	40.5	36
**Incidence rate**	0.27	0.14

For peer review only

Supplementary material 7. Changes in exercise capacity and physical function after six months.

	Outcome		Usual Care	Exercise	Difference (95% CI)
**ISWT** (m)		n	16	15	36 (-12 to 84)
		Baseline	184 ± 130	237 ± 173	
		Final	158 ± 154	248 ± 192	
		Change	-26 ± 68	11 ± 63	
**ESWT** (secs)		n	14	15	181 (-92 to 453)
		Baseline	347 ± 384	401 ± 375	
		Final	193 ± 304	428 ± 423	
		Change	-153 ± 286	27 ± 413	
**STS60** (n)		n	17	15	0 (-5 to 4)
		Baseline	10 ± 12	13 ± 11	
		Final	10 ±13	13 ± 12	
		Change	0 ± 7	0 ± 6	
**SPPB**	Total score	n	17	15	0.5 (-0.7 to 2)
		Baseline	7 ± 3	9 ± 3	
		Final	6 ± 2	8 ± 3	
		Change	-1 ± 2	-0.5 ± 1	
	4m walk time (secs)	n	17	15	1 (-1 to 4)
		Baseline	7 ± 6	4± 1	
		Final	6 ± 4	5 ± 2	
		Change	1 ± 5	0 ± 1	
	Gait speed (m/s)	n	17	15	0.05 (-0.12 to 0.22)
		Baseline	0.74 ± 0.29	0.96 ± 0.28	
		Final	0.74 ± 0.28	0.91 ± 0.31	
		Change	0.00 ± 0.22	-0.05 ± 0.24	
	STS5 (secs)	n	9	10	5 (-4 to 15)
		Baseline	17 ± 7	16 ± 14	
		Final	23 ± 13	16 ± 10	
		Change	6 ± 11	0 ± 8.	

Abbreviations: CI, confidence interval; ESWT, Endurance Shuttle Walk Test; ISWT, Incremental Shuttle Walk Test; m/s, metres per second; Secs, seconds; SPPB, Short Physical Performance Battery; STS5, Sit to Stand Five Repetitions; STS60, Sit to Stand in Sixty Seconds.

For peer review only

Supplementary material 8. Changes in physical activity (accelerometry data) after six months.

	Type of day		Usual Care	Exercise	Difference (95% CI)
**Waking wear time** (mins)	HD	n	5	10	244 (16 to 473)
		Baseline	891 ± 202	818 ± 183	
		Final	749 ± 105	921 ± 171	
		Change	-142 ±166	103 ± 204	
	Non-HD	n	5	10	170 (-13 to 353)
		Baseline	893 ± 90	927 ± 216	
		Final	817 ± 134	1022 ± 165	
		Change	-75 ± 201	95 ± 129	
**Steps** (steps/day)	HD	n	5	10	859 (-825 to 2543)
		Baseline	2252 ± 4210	1373 ± 1080	
		Final	2464 ± 4783	2444 ± 1904	
		Change	211 ± 593	1070 ± 1665	
	Non-HD	n	5	10	888 (-84 to 1861)
		Baseline	3076 ± 5790	2387 ± 1696	
		Final	2645 ± 5284	2845 ± 2117	
		Change	-430 ± 603	458 ± 903	
**Sedentary** (mins/ day)	HD	n	5	10	28 (-284 to 340)
		Baseline	954 ±338	954 ± 203	
		Final	965 ± 208	992 ± 182	
		Change	10 ± 200	38 ± 287	
	Non-HD	n	5	10	124 (-205 to 454)
		Baseline	1022 ± 357	1103 ± 253	
		Final	912 ± 224	1117 ± 174	
		Change	-110 ± 298	14 ± 269	
**Light PA** (mins/day)	HD	n	5	10	91 (23 to -158)
		Baseline	125 ± 51	83 ± 42	
		Final	79 ± 39	127 ± 73	
		Change	-46 ± 45	44 ± 62	
	Non-HD	n	5	10	9 (-71 to 91)
		Baseline	145 ± 59	133 ± 50	
		Final	154 ± 99	151 ± 59	
		Change	9 ± 108	18 ± 44	
**Moderate PA** (mins/day)	HD	n	5	10	13 (-32 to 57)
		Baseline	83 ± 105	29 ± 33	
		Final	85 ± 123	43 ± 55	
		Change	1 ± 52	14 ± 29	
	Non-HD	n	5	10	20 (40 to -79)
		Baseline	79 ± 96	46 ± 61	
		Final	75 ± 112	62 ± 105	
		Change	-4 ± 40	16 ± 55	
**Vigorous PA** (mins/day)	HD	n	5	10	3 (-1 to 8)
		Baseline	4 ± 9	1 ± 1	
		Final	1 ± 2	1 ± 3	
		Change	-3 ± 7	0 ± 2	
	Non-HD	n	5	10	1 (0 to 2)
		Baseline	3 ± 0	1 ± 4	
		Final	2 ± 5	1 ± 4	
		Change	-1 ± 2	0 ± 0	

Abbreviations: CI, confidence interval; HD, haemodialysis; mins, minutes; PA, physical activity.

For peer review only

Supplementary material 9. Patient-reported outcomes measures after six months.

	Outcome		Usual Care	Exercise	Difference (95% CI)
SF-12	PCS	n	19	19	0 (-4 to 5)
		Baseline	35 ± 9	35 ± 10	
		Final	36 ± 10	36 ± 10	
		Change	1 ± 7	1 ± 7	
	MCS	n	19	19	4 (-3 to 10)
		Baseline	43 ± 15	45 ± 13	
		Final	46 ± 13	45 ± 13	
		Change	4 ± 7	0 ± 12	
HADS		n	20	17	0 (-3 to 4)
		Baseline	16 ± 10	15 ± 9	
		Final	14 ± 10	13 ± 9	
		Change	-2 ± 5	-2 ± 6	
POS-R	Global severity score	n	20	18	2 (-3 to 7)
		Baseline	19 ± 14	19 ± 14	
		Final	18 ± 14	20 ± 14	
		Change	1 ± 6	-1 ± 9	
	mean severity	n	20	18	0 (0 to 0)
		Baseline	2 ± 1	2 ± 1	
		Final	2 ± 1	2 ± 1	
		Change	0 ± 0	0 ± 0	
	mean number	n	22	16	0 (-1 to 2)
		Baseline	9 ± 4	10 ± 4	
		Final	9 ± 4	10 ± 5	
		Change	0 ± 4	0 ± 2	
ESES		n	19	16	0 (-1 to 1)
		Baseline	2 ± 2	2 ± 1	
		Final	2 ± 1	2 ± 1	
		Change	0 ± 1	0 ± 1	
DPPEBBS		n	19	15	3 (-4 to 11)
		Baseline	59 ± 10	59 ± 15	
		Final	61± 10	65 ± 7	
		Change	2 ± 7	6 ± 14	
DASI		n	20	18	4.93 (-0.94 to 10.80)
		Baseline	13.06 ± 12.85	20.29 ± 14.33	
		Final	17.29 ± 14.41	19.60 ± 14.59	
		Change	4.22 ± 9.72	-0.71 ± 7.92	

Abbreviations: CI, confidence interval; DASI, Duke Activity Status Index; DPPEBBS, Dialysis Patients Benefits and Barriers Scale; ESES, Exercise Self efficacy Scale; HADS, Hospital Anxiety and Depression Scale; MCS, mental component summary score; POS-R, Palliative Outcomes Scale Renal, PCS, physical component summary score; VAS, visual analogue scale.

For peer review only

Supplementary material 10. Baseline demographic and clinical characteristics for the qualitative participants.

		**N=25**
**Age** (years)		69±10
**Gender** n (%)	Female	13 (52%)
	Male	12 (48%)
**Ethnicity** n (%)	White background	13 (52%)
	Asian or Asian British	10 (40%)
	Caribbean	1 (4%)
	Not stated	1 (4%)
**Diagnosis**	Diabetic nephropathy	11 (44%)
	Aetiology uncertain	6 (24%)
	Chronic pyelonephritis	3 (12%)
	Atypical hemolytic uremic syndrome	1 (4%)
	FSGS	1 (4%)
	Henoch-Schönlein Purpura	1 (4%)
	Minimal change nephropathy	1 (4%)
	Polycystic kidney disease	1 (4%)
**CCI**		6±2
**Time on HD** (months)		43 (IQR 16-85)
**CFS** n (%)	Vulnerable	9 (36%)
	Mildly frail	5 (20%)
	Moderately frail	8 (32%)
	Severely frail	3 (12%)
**Number of falls in the last six months**		3 (IQR 2-4)
**Previous transplant** n (%)	No	21 (84%)
	Yes	4 (16%)
**Active on transplant list** n (%)	No	22 (88%)
	Yes	3 (12%

Abbreviations: CCI, Charlson comorbidity index; CFS, clinical frailty scale; FSGS, Focal segmental glomerulosclerosis; HD, haemodialysis.

For peer review only

Supplementary material 11. Joint display of quantitative and qualitative results, with an overall assessment of mixed-methods inferences.

	Progression criteria	Feasibility trial	Qualitative results	Mixed-methods inferences
**Eligibility**	**STOP** <20%<br>**GO** >50%<br>eligible.	31% patients eligible	No discussion. Patients not involved in screening process	Silence
**Recruitment**	**STOP** <25%<br>**GO** >50%<br>recruited.	52% eligible patients recruited.	- Frailer and female participants less likely to be approached despite eligibility and have more concerns about the suitability<br>- Perception that risks outweigh the potential benefit<br>- Recruitment processes could be improved	Complementary
**Retention**	**STOP** >40%<br>**GO** <20%<br>lost to follow-up.	12 % loss to follow-up. Reasons predominantly unavoidable (death, ill-health).	Loss to follow-up attributed to:<br>- Illness;<br>- length of trial;<br>- the reality of being in the study not meeting expectations.	Complementary
**Intervention**	**STOP** <30%<br>**GO** >70%<br>adherence over six-months.	74% adherence rate across the six-month exercise duration.	- IDC good use of time.<br>- Participants felt safe and felt well supported.<br>- IDC limited in scope.<br>- Participants described a range of other important components	Complementary
**Outcome**	**STOP** <70%<br>**GO** >80%<br>outcome measure completion.	Up to 89% of secondary outcome measure data missing<br>Collection of falls data challenging.	- Number of outcomes measured to be reduced.<br>- Outcome testing during HD or at home preferred.<br>- 52% agreed to complete a falls diary, 12% lost.<br>- STS60, ESWT and ISWT unsuitable<br>- Researcher support and family involvement may increase completion<br>- Outcomes measuring ADLs, participation and symptom prioritised	Complementary<br><br>Silence for PA monitoring.

Results from the feasibility trial are colour coded to depict whether they met the 'stop' (red), 'go' (green) or 'change (orange) progression criteria. Abbreviations: ADLs, activities of daily living; ESWT, Endurance Shuttle Walk Test; IDC, intradialytic exercise; ISWT, Incremental Shuttle Walk Test; PA, physical activity; STS60, sit to stand in sixty seconds.

For peer review only - http://bmjopen.bmj.com/site/about/guidelines.xhtml

CONSORT 2010 checklist of information to include when reporting a pilot or feasibility trial*

Section/Topic	Item No	Checklist item	Reported on page No
**Title and abstract**			
	1a	Identification as a pilot or feasibility randomised trial in the title	1
	1b	Structured summary of pilot trial design, methods, results, and conclusions (for specific guidance see CONSORT abstract extension for pilot trials)	3-4
**Introduction**			
Background and objectives	2a	Scientific background and explanation of rationale for future definitive trial, and reasons for randomised pilot trial	6
	2b	Specific objectives or research questions for pilot trial	6-7
**Methods**			
Trial design	3a	Description of pilot trial design (such as parallel, factorial) including allocation ratio	7-8
	3b	Important changes to methods after pilot trial commencement (such as eligibility criteria), with reasons	n/a
Participants	4a	Eligibility criteria for participants	7 and supplementary material 1
	4b	Settings and locations where the data were collected	7-8,10-11
	4c	How participants were identified and consented	8
Interventions	5	The interventions for each group with sufficient details to allow replication, including how and when they were actually administered	8 and supplementary material 2
Outcomes	6a	Completely defined prespecified assessments or measurements to address each pilot trial objective specified in 2b, including how and when they were assessed	9-11, supplementary materials 3,4 and 5
	6b	Any changes to pilot trial assessments or measurements after the pilot trial commenced, with reasons	n/a
	6c	If applicable, prespecified criteria used to judge whether, or how, to proceed with future definitive trial	9 and supplementary material 3
Sample size	7a	Rationale for numbers in the pilot trial	8-9

For peer review only

	7b	When applicable, explanation of any interim analyses and stopping guidelines	9
Randomisation:			
Sequence generation	8a	Method used to generate the random allocation sequence	8
	8b	Type of randomisation(s); details of any restriction (such as blocking and block size)	8
Allocation concealment mechanism	9	Mechanism used to implement the random allocation sequence (such as sequentially numbered containers), describing any steps taken to conceal the sequence until interventions were assigned	8
Implementation	10	Who generated the random allocation sequence, who enrolled participants, and who assigned participants to interventions	8
Blinding	11a	If done, who was blinded after assignment to interventions (for example, participants, care providers, those assessing outcomes) and how	9
	11b	If relevant, description of the similarity of interventions	n/a
Statistical methods	12	Methods used to address each pilot trial objective whether qualitative or quantitative	11
**Results**			
Participant flow (a diagram is strongly recommended)	13a	For each group, the numbers of participants who were approached and/or assessed for eligibility, randomly assigned, received intended treatment, and were assessed for each objective	12-13 figure 1
	13b	For each group, losses and exclusions after randomisation, together with reasons	12-13 figure 1
Recruitment	14a	Dates defining the periods of recruitment and follow-up	12
	14b	Why the pilot trial ended or was stopped	n/a
Baseline data	15	A table showing baseline demographic and clinical characteristics for each group	Table 1 (trial) supplementary material 10 (qualitative)
Numbers analysed	16	For each objective, number of participants (denominator) included in each analysis. If relevant, these numbers should be by randomised group	Supplementary materials6-9 and page 15-17
Outcomes and estimation	17	For each objective, results including expressions of uncertainty (such as 95% confidence interval) for any estimates. If relevant, these results should be by randomised group	Supplementary materials 6-9 and page 15-17
Ancillary analyses	18	Results of any other analyses performed that could be used to inform the future definitive trial	17- 26
Harms	19	All important harms or unintended effects in each group (for specific guidance see CONSORT for harms)	P 17
	19a	If relevant, other important unintended consequences	n/a
**Discussion**			

For peer review only

Limitations	20	Pilot trial limitations, addressing sources of potential bias and remaining uncertainty about feasibility	30
Generalisability	21	Generalisability (applicability) of pilot trial methods and findings to future definitive trial and other studies	26-31
Interpretation	22	Interpretation consistent with pilot trial objectives and findings, balancing potential benefits and harms, and considering other relevant evidence	26-31
	22a	Implications for progression from pilot to future definitive trial, including any proposed amendments	26-31
**Other information**			
Registration	23	Registration number for pilot trial and name of trial registry	4 and 7
Protocol	24	Where the pilot trial protocol can be accessed, if available	7
Funding	25	Sources of funding and other support (such as supply of drugs), role of funders	31
	26	Ethical approval or approval by research review committee, confirmed with reference number	32

Citation: Eldridge SM, Chan CL, Campbell MJ, Bond CM, Hopewell S, Thabane L, et al. CONSORT 2010 statement: extension to randomised pilot and feasibility trials. BMJ. 2016;355.
*We strongly recommend reading this statement in conjunction with the CONSORT 2010, extension to randomised pilot and feasibility trials, Explanation and Elaboration for important clarifications on all the items. If relevant, we also recommend reading CONSORT extensions for cluster randomised trials, non-inferiority and equivalence trials, non-pharmacological treatments, herbal interventions, and pragmatic trials. Additional extensions are forthcoming: for those and for up to date references relevant to this checklist, see www.consort-statement.org.

For peer review only

BMJ Open

Exercise for people living with frailty and receiving haemodialysis: a mixed-methods randomised controlled feasibility study.

Journal:	*BMJ Open*
Manuscript ID	bmjopen-2020-041227.R2
Article Type:	Original research
Date Submitted by the Author:	06-Sep-2020
Complete List of Authors:	Young, Hannah; University of Leicester, Department of Respiratory Sciences; University Hospitals of Leicester NHS Trust, Department of Research and Innovation<br>March, Daniel; University of Leicester, Department of Cardiovascular Sciences<br>Highton, Patrick; University of Leicester, Department of Cardiovascular Sciences; Loughborough University, National Centre for Sport and Exercise Medicine<br>Graham-Brown, Matthew ; University of Leicester, Cardiovascular Sciences; Loughborough University, National Centre for Sport and Exercise Medicine<br>Churchward, Darren; University of Leicester, Department of Cardiovascular Sciences<br>Grantham, Charlotte; University of Leicester, Department of Cardiovascular Sciences<br>Goodliffe, Samantha; University of Leicester, Department of Health Sciences<br>Jones, William; University Hospitals of Leicester, Emergency Department, Leicester Royal Infirmary,<br>Cheung, Mei-Mei; University Hospitals of Leicester, Renal, Respiratory and Cardiovascular Clinical Management Group<br>Greenwood, Sharlene ; King's College Hospital, Department of Physiotherapy and Renal Medicine; King's College London, Department of Renal Medicine<br>Eborall, Helen; University of Leicester, Department of Health Sciences<br>Conroy, Simon; University of Leicester, Department of Health Sciences<br>Singh, Sally; Leicester Biomedical Research Unit, Centre for Exercise & Rehabilitation Science; University Hospitals of Leicester NHS Trust, Department of Respiratory Medicine, Glenfield Hospital<br>Smith, Alice; University of Leicester, Department of Health Sciences<br>Burton, James; University of Leicester, Department of Cardiovascular Sciences; Loughborough University, National Centre for Sport and Exercise Medicine
**Primary Subject Heading**:	Renal medicine
Secondary Subject Heading:	Rehabilitation medicine
Keywords:	Dialysis < NEPHROLOGY, End stage renal failure < NEPHROLOGY,

	REHABILITATION MEDICINE, QUALITATIVE RESEARCH, Clinical trials < THERAPEUTICS

SCHOLARONE™ Manuscripts

I, the Submitting Author has the right to grant and does grant on behalf of all authors of the Work (as defined in the below author licence), an exclusive licence and/or a non-exclusive licence for contributions from authors who are: i) UK Crown employees; ii) where BMJ has agreed a CC-BY licence shall apply, and/or iii) in accordance with the terms applicable for US Federal Government officers or employees acting as part of their official duties; on a worldwide, perpetual, irrevocable, royalty-free basis to BMJ Publishing Group Ltd ("BMJ") its licensees and where the relevant Journal is co-owned by BMJ to the co-owners of the Journal, to publish the Work in this journal and any other BMJ products and to exploit all rights, as set out in our licence.

The Submitting Author accepts and understands that any supply made under these terms is made by BMJ to the Submitting Author unless you are acting as an employee on behalf of your employer or a postgraduate student of an affiliated institution which is paying any applicable article publishing charge ("APC") for Open Access articles. Where the Submitting Author wishes to make the Work available on an Open Access basis (and intends to pay the relevant APC), the terms of reuse of such Open Access shall be governed by a Creative Commons licence – details of these licences and which Creative Commons licence will apply to this Work are set out in our licence referred to above.

Other than as permitted in any relevant BMJ Author's Self Archiving Policies, I confirm this Work has not been accepted for publication elsewhere, is not being considered for publication elsewhere and does not duplicate material already published. I confirm all authors consent to publication of this Work and authorise the granting of this licence.

For peer review only

Exercise for people living with frailty and receiving haemodialysis: a mixed-methods randomised controlled feasibility study.

Hannah ML Young (MSc)[1,2], Daniel S March (PhD)[3], Patrick J Highton (PhD)[3,4], Matthew PM Graham-Brown (PhD)[3,4], Darren C Churchward (MSc)[3], Charlotte Grantham (BSc)[3], S Goodliffe (MSc)[5], William Jones (BAHons)[6], Mei-Mei Cheung (BAHons)[7], Sharlene A Greenwood (PhD)[8], Helen Eborall (PhD)[9], Simon Conroy (PhD)[5], Sally J Singh (PhD)[10,11], Alice C Smith (PhD)[5], James O Burton (MD)[3,4].

[1]Department of Respiratory Sciences, University of Leicester, Leicester, UK.

[2]Department of Research and Innovation, University Hospitals of Leicester, UK

[3]Cardiovascular Sciences, University of Leicester, UK.

[4]National Centre for Sport and Exercise Medicine, Loughborough University, UK.

[5]Department of Health Sciences, University of Leicester, UK.

[6] Emergency Department, Leicester Royal Infirmary, University Hospitals of Leicester, UK.

[7] Renal, Respiratory and Cardiovascular Clinical Management Group, University Hospitals of Leicester, UK.

[8]Department of Physiotherapy and Renal Medicine, King's College Hospital and Department of Renal Medicine, King's College London, UK.

[9]Social Science Applied to Healthcare Improvement Research (SAPPHIRE) Group, Department of Health Sciences, University of Leicester, UK.

For peer review only

For peer review only - http://bmjopen.bmj.com/site/about/guidelines.xhtml

[10]Centre for Exercise & Rehabilitation Science, Leicester Biomedical Research Unit, Glenfield Hospital, UK.

[11]Department of Respiratory Medicine, Glenfield Hospital, University Hospitals of Leicester,UK.

Corresponding author:

Hannah Young

Leicester Kidney Lifestyle Team

Academic Unit

Leicester General Hospital

Gwendolen Road

Leicester

UK

LE4 5PW

0116 258 4346

hmly1@le.ac.uk

Running title: Exercise for frail haemodialysis patients

Abstract word count: 280

Word count: 4884

For peer review only - http://bmjopen.bmj.com/site/about/guidelines.xhtml

ABSTRACT

Objectives

Frailty is highly prevalent in haemodialysis (HD) patients, leading to poor outcomes. This study aimed to determine whether a Randomised Controlled Trial (RCT) of intradialytic exercise is feasible for frail HD patients, and explore how the intervention may be tailored to their needs.

Design

Mixed-methods feasibility.

Setting & participants

Prevalent adult HD patients of the *CYCLE-HD* trial with a Clinical Frailty Scale Score of 4-7 (vulnerable to severely frail) were eligible for the feasibility study.

Interventions

Participants in the exercise group undertook six-months of thrice-weekly, progressive, moderate intensity intradialytic cycling (IDC).

Outcomes

Primary outcomes were related to feasibility. Secondary outcomes were falls incidence measured from baseline to one year following intervention completion, and exercise capacity, physical function, physical activity and patient-reported outcomes (PROMS) measured at baseline and six months. Acceptability of trial procedures and the intervention were explored via diaries and interviews with n=25 frail HD patients who both participated in (n=13, 52%), and declined (n=12, 48%), the trial.

Results

For peer review only - http://bmjopen.bmj.com/site/about/guidelines.xhtml

124 (31%) patients were eligible, and of these 64 (52%) consented with 51 (80%) subsequently completing a baseline assessment. N=24 (71% male; 59 ± 13 years) dialysed during shifts randomly assigned to exercise and n=27 (81% male; 65 ± 11 years) shifts assigned to usual care. N=6 (12%) were lost to follow-up. The exercise group completed 74% of sessions. 27 to 89% of secondary outcome data were missing. Frail HD patients outlined several ways to enhance trial procedures. Maintaining ability to undertake activities of daily living and social participation were outcomes of primary importance. Participants desired a varied exercise programme.

Conclusions

A definitive RCT is feasible, however a comprehensive exercise programme may be more efficacious than IDC in this population.

Trial Registration

ISRCTN11299707; ISRCTN12840463

Keywords: feasibility; frailty; exercise; haemodialysis; mixed-methods.

For peer review only

STRENGTHS AND LIMITATIONS OF THIS STUDY

- To our knowledge, this is the first study to evaluate the feasibility of an exercise intervention for people living with frailty and receiving haemodialysis (HD).
- The Clinical Frailty Scale, a frailty risk-stratification measure which has been validated in an HD population, was used to identify eligible participants.
- This study is also the first to explore how trial procedures and exercise programmes should be specifically tailored to the needs of people living with frailty and receiving HD, from their own perspectives.
- Multiple qualitative methods (interviews and diaries) were used to explore participants perceptions, providing a form of triangulation which strengthens the conclusions made.
- Due to the nature of the intervention and resource limitations, we could not blind intervention providers, outcome assessors or study participants to group allocation.

For peer review only - http://bmjopen.bmj.com/site/about/guidelines.xhtml

INTRODUCTION

Frailty, “a multidimensional syndrome of decreased physiological reserve leading to increased vulnerability to minor health stressors”, is highly prevalent within the haemodialysis (HD) population.[1,2] Increasing frailty is associated with worsening outcomes, including mortality, hospitalisation, falls, reduced Health-Related Quality of Life (HRQoL), psychological well-being, physical function, ability to undertake activities of daily living (ADLs) and increased symptom burden.[3-5]

Despite this, frailty is not static and evidence suggests that some factors associated with frailty are amenable to change.[6] Whilst the possible mediating role of exercise has been discussed, to our knowledge no original studies have examined the feasibility or effectiveness of an exercise programme for people living with frailty and receiving HD.[7] To date, exercise interventions for HD patients have focused upon intradialytic exercise, most commonly delivered by means of a cycle ergometer (intradialytic cycling, IDC), yet little is known about whether this is the most appropriate training stimulus for frail HD patients.[8] In addition, HD treatment can be poorly tolerated by frail patients and therefore IDC may represent an additional stressor to which these patients are particularly vulnerable.[9] European renal best practice guidance highlights a need for studies which identify how exercise programmes should be more specifically tailored to the needs of frail CKD patients[10], yet to date, there has also been no exploration of the needs, barriers and facilitators to exercise from the perspectives of people living with frailty and receiving HD themselves.

The aim of this study was to determine the feasibility of conducting an RCT investigating the effects of IDC for HD patients living with frailty by: (i) estimating rates of eligibility,

For peer review only - http://bmjopen.bmj.com/site/about/guidelines.xhtml

recruitment, retention, exercise adherence and outcome acceptability; and exploring (ii) the potential benefits of IDC across a range of secondary outcomes; and (iii) the perceptions of frail HD patients in relation to participating in clinical research, IDC and a tailored exercise intervention.

METHODS

Design

A prospective, randomised controlled feasibility study was conducted alongside concurrent qualitative diaries and interviews (Trial Registration numbers ISRCTN11299707; ISRCTN12840463). The feasibility study was a secondary analysis of the *CYCLE-HD* trial, whose aims and methods are reported elsewhere.[11] The qualitative component was underpinned by a constructivist Grounded Theory approach.[12] All participants provided written informed consent.

Participants

Prevalent adult (over 18 years) HD patients were recruited from three centres within the UK East Midlands Renal Network. In addition to the inclusion and exclusion criteria for the *CYCLE-HD* trial (supplementary material 1), the Clinical Frailty Scale (CFS), a risk stratification tool, was used to identify vulnerable to severely frail participants (CFS score 4-7).[13] The CFS has good predictive abilities in an HD population, good construct validity when compared with the Frailty Index, is less burdensome that the Frailty Phenotype, and has been validated in an HD population. [13-15]

For peer review only - http://bmjopen.bmj.com/site/about/guidelines.xhtml

The inclusion and exclusion criteria for the qualitative component mirrored the feasibility study and both those involved in the trial, and those who were eligible but declined to participate, were eligible.

Randomisation

HD cohorts were randomised prior to screening, based on a computer-generated randomisation algorithm held by the Robertson Centre for Biostatistics at the University of Glasgow.

Recruitment

Patients were screened for eligibility by their supervising nephrologist. Suitable patients were approached during HD, and the study explained. For the qualitative component, participants who had been involved in the feasibility study were recruited following completion of, or withdrawal from, the trial to prevent contamination.

Exercise intervention

Supplementary material 2 outlines the exercise intervention in line with TIDieR guidance.[16] Briefly, following a one-month run-in, participants in the exercise group undertook thrice-weekly supervised, moderate-intensity (Rating of Perceived Exertion, RPE 12-14) IDC (MOTOmed Letto2, Reck, Germany), for six months.[17] Cycling resistance was progressively increased to maintain RPE in response to exercise adaptation. Both arms continued with usual care HD as described elsewhere.[11]

For peer review only - http://bmjopen.bmj.com/site/about/guidelines.xhtml

For peer review only

Sample size

Determinations of sample size from a power calculation around a primary outcome are not relevant to a feasibility study and sample sizes of 24-50 are considered sufficient.[18] For the qualitative component maximum variation sampling was initially used to ensure diversity in frailty status and level of trial participation.[12] As understanding was gained from preliminary analyses, theoretical sampling was used to further recruit participants.[12] A maximum of 30 interviews were planned, but data collection ceased at the point where theoretical categories were saturated and no longer generated new insight (n=25).

Primary outcome measures

The primary feasibility outcomes are presented in supplementary material 3. Judgement regarding feasibility was based upon a set of *a priori* progression criteria. For each criterion, the development of 'stop'(indicating when there are issues with the trial that cannot be resolved) and 'go' thresholds (when there are no issues that may impede the success of a trial) were co-produced by patients, clinicians and researchers.[19,20] Results falling between these thresholds indicated that adaptation to trial procedures may render a definitive RCT viable.[20]

Baseline demographic and clinical variables

Demographic and clinical characteristics were gathered from participants' medical notes. The Charlson Comorbidity Index (CCI) was used to estimate the burden of comorbid disease.[21]

For peer review only - http://bmjopen.bmj.com/site/about/guidelines.xhtml

Secondary outcome measures

Multiple secondary outcomes were used to determine the potential effects of IDC and most appropriate primary endpoint for a future RCT. Outcome assessors were not blinded to group allocation.

Information on the number of falls, defined as 'an unexpected event in which the participants come to rest on the ground, floor, or lower-level' which resulted in Emergency Department visits and hospital admissions were collected from baseline to one year following intervention completion from medical records and hospital episode statistics.[22]

Field tests of exercise capacity and physical function included the Incremental Shuttle Walk Test (ISWT), the Endurance Shuttle Walk Test (ESWT), the Short Physical Performance Battery (SPPB) and the Sit-to Stand in Sixty Seconds (STS60).[11] Physical activity (PA) was objectively measured using the SenseWear Armband (SWA) Pro 3 (BodyMedia, Inc., Pittsburgh PA, USA) for seven consecutive days, including HD. Established criteria were used to ensure representative data for average daily wear-time, steps per day, and time (minutes per day) spent in sedentary (defined as 0-1.5 METS), light (1.6-2.9 METS) moderate (3-6 METS) and vigorous (>6 METS) PA.[23] PROMs collected are outlined in supplementary material 4.[11] All outcomes were collected at baseline and six months.

Serious adverse events (SAEs) were recorded and assessed from baseline to six-months as outlined previously.[11]

For peer review only - http://bmjopen.bmj.com/site/about/guidelines.xhtml

Diaries and interviews

Participants first completed a prospective falls diary, recognised as the current 'gold standard' for falls data collection, for up to three months to examine the feasibility of this outcome measure within a future definitive RCT.[22] Semi-structured interviews then explored participants' experiences of: (i) keeping a falls diary; (ii) participating in a trial; and (iii) their perceptions of IDC and a tailored exercise intervention.

Information to support diary collection and a topic guide for the interviews (supplementary material 5) was developed by HMLY, HE and a patient and public involvement group. Topics were tailored according to the level of involvement in the trial, and the content of diaries. Interviews were conducted during HD, in the participant's home, or in the hospital by HMLY and lasted 20 to 120 minutes (mean 63 minutes). All were digitally audio-recorded and transcribed verbatim.

Data analysis

Sample characteristics are presented as mean ± standard deviation, median (IQR) or n (%), as appropriate. Descriptive statistics and confidence intervals were used to estimate feasibility outcomes.[24] The percentage of exercise sessions completed was used to establish the acceptability of IDC. Outcome acceptability was determined by quantifying the amount of missing data across secondary outcomes. No imputation was performed to account for missing data. No statistical testing relating to the efficacy of the exercise intervention was undertaken, although the potential benefits of exercise were estimated.[24] For falls, summary data, incident rate ratio (the ratio of the incidence rate in the exercise group divided by the incidence rate in the usual care group) and 95% confidence intervals were presented.

For peer review only - http://bmjopen.bmj.com/site/about/guidelines.xhtml

Statistical analyses were performed using SPSS 24 (IBM UK Ltd, UK) and Stata 16 (StataCorp LCC,USA).

Qualitative analysis was undertaken by HMLY and SG and informed by a constant comparative approach.[12] Transcripts were reviewed, then coded line by line, followed by focused, and then theoretical, coding.[12] NVivo11 software (QSR International Ltd, version 11, 2016) was used to facilitate data management. Finally, qualitative and quantitative results were merged in a 'joint display' to facilitate an overall assessment of feasibility.[25]

Patient and public involvement

The patient and public involvement (PPI) group for this study comprised patients of all ages, genders and ethnicities who were living with frailty and receiving HD, and their relatives. They agreed this study was an important priority for further investigation and particularly stressed the need to add the qualitative component. The PPI group were involved early in the ethical approval stages and were actively engaged in writing lay summaries and providing patient perspectives on data collection procedures, ethical issues, and the study dissemination plans. They assisted in the preparation of study documentation, interview topic guides and diary keeping materials. During the study, members of the PPI group attended regular steering meetings and were involved in co-producing the progression criteria.

RESULTS

Feasibility study

For peer review only - http://bmjopen.bmj.com/site/about/guidelines.xhtml

Eligibility and recruitment

Screening and recruitment took place from March 2015 to 2018, with data collection completed by November 2018. Figure 1 outlines the trial CONSORT. Of the 406 patients screened in the *CYCLE-HD* trial, n=124 (30%, 95% CI 26.1% to 35.3%) were identified as vulnerable to severely frail and therefore eligible for the feasibility study. Sixty-four participants (52%, 95% CI 42.5% to 60.7%) consented. Reasons for declining were lack of time or family support and reluctance to undergo outcome testing, or to be randomised. Those who declined to participate had a median age of 73 (IQR 67-81) years. N=35 (58%) were female and n=27 (42%) male. Twenty-five (42%) were classified as vulnerable according to the CFS, n=17 (28%) were mildly frail, n=9 (15%) moderately frail and n=9 (15%) severely frail. Thirteen (20%, 95% CI 11.3% to 32.2%) participants withdrew prior to baseline assessment. N=51 (80%, 95% CI 67.8% to 88.7%) completed this assessment. Twenty-four (47%) participants received dialysis during shifts randomised to exercise and twenty-seven (53%) during shifts randomised to usual care.

[FIGURE ONE TRIAL CONSORT TO BE INSERTED HERE]

Participant characteristics

Table 1 displays the characteristics of the trial participants at baseline. Groups were well matched across most variables. A lower proportion of participants were female (23.5%) and severely frail (6%) overall.

For peer review only - http://bmjopen.bmj.com/site/about/guidelines.xhtml

Table 1. Baseline demographic and clinical characteristics of the trial participants.

		Usual care (n=27)	Exercise (n=24)	All (n=51)
Age (years)		65 ± 11	59 ± 13	63 ± 12
Sex (n, %)	Female	5 (18.5%)	7 (29%)	12 (23.5%)
Ethnicity (n, %)	White	12 (44%)	11 (46%)	23 (45%)
	Asian or Asian British	11 (41%)	11 (46%)	22 (43%)
	Caribbean	1 (4%)	0 (0%)	1 (2%)
	Other ethnic	1 (4%)	1 (4%)	2 (4%)
	Not stated	2 (7%)	1 (4%)	3 (6%)
Diagnosis (n, %)	Aetiology Uncertain	8 (29%)	7 (29%)	15 (29%)
	Diabetic Nephropathy	5 (19%)	7 (29%)	12 (23%)
	Glomerulonephritis	5 (19%)	3 (14%)	8 (16%)
	Renal Vascular Disease	3 (11%)	2 (8%)	5 (10%)
	Other diagnoses	4 (15%)	1 (4%)	5 (10%)
	Chronic Pyelonephritis	2 (7%)	1 (4%)	3 (6%)
	Polycystic Kidney Disease	0 (0%)	2 (8%)	2 (4%)
	Not recorded	0 (0%)	1 (4%)	1 (2%)
CCI		5 ± 2	5 ± 2	5 ± 2
Previous transplant (n, %)	No	21 (75%)	18 (75%)	39 (76.5%)
	Yes	6 (21%)	6 (25%)	12 (23.5%)
Time on HD (months)		17 (7-53)	13 (10-61)	16 (8-53)
BMI (kg/m^2)		27.38 ± 6.72	25.87 ± 5.28	26.67 ± 6.07
Total no. medications		12 ± 4	12 ± 4	12 ± 4
Clinical Information	Albumin (g/L)	35.4 ± 4.4	37.4 ± 4.3	36.4 ± 4.4
	Haemoglobin (g/L)	107 ± 12	112 ± 17	107 ± 15
Haemodialysis	URR (%)*	74 (70-80)	75 (58-79)	74 (71-79)
	SBP (mmHg)	143 ± 21	144 ± 21	144 ± 21
	DBP (mmHg)*	65 (62-78)	78 (69-86)	76 (62-81)
CFS (n, %)	4, Vulnerable	13 (48%)	10 (42%)	23 (45%)
	5, Mildly frail	5 (18.5%)	7 (29%)	12 (23.5%)
	6, Moderately frail	8 (30%)	5 (21%)	13 (25.5%)
	7, Severely frail	1 (3.5%)	2 (8%)	3 (6%)

*Values reported are mean and SD (±), except for *median and IQR. Abbreviations: BMI, body mass index; CCI, Charlson Comorbidity Index; CFS, Clinical Frailty Scale DBP, diastolic blood pressure; SBP, systolic blood pressure; URR, urea reduction ratio.*

For peer review only - http://bmjopen.bmj.com/site/about/guidelines.xhtml

Retention

Six (12%, 95% CI 4.4% to 23.9%) participants were lost to follow-up: three participants withdrew due to ill-health, one moved away, one changed HD regime and one withdrew consent.

Exercise adherence

A mean of 61±17 exercise sessions were completed over the six-month intervention, representing an adherence rate of 74±20%. The most frequent reasons for missing an exercise session were declining (n= 175 out of 535 sessions omitted in total, 33%), feeling unwell (n= 116, 22%) and pain (n= 105, 20%). Table 2 summarises the mean amount of exercise achieved. On average, participants reached the prescribed level of exercise by six months, although n=18 (75%) were unable to achieve this by the end of the one-month run-in period.

Table 2 Mean (SD) exercise achieved per session over the six-month duration of the intervention.

Duration (mins)	35 ± 8
Speed (RPM)	63 ± 10
Intensity (RPE)	13 ± 1
Gear	9 ± 4
Distance (Miles)	7 ± 3
Power (Watts)	13 ± 6
Energy expenditure (Kcals)	64 ± 31

All data presented as mean and SD (±). Abbreviations: kcals, kilocalories, mins, minutes; RPE, rating of perceived exertion; RPM, revolutions per minute.

Outcome acceptability

For tests of exercise capacity (ISWT and ESWT); n=14 (27%) did not complete at least one test at baseline, n=30 (64%) at interim and n=26 (58%) at final. For tests of physical function; n=20 (39%) did not complete at least one test at baseline, n=33 (70%) at interim and n=30 (67%) at final. For PROMs; n=27 (53%) did not complete at least one questionnaire at

For peer review only - http://bmjopen.bmj.com/site/about/guidelines.xhtml

baseline, n=27 (57%) at interim and n=40 (89%) at final. For PA data; n=21 (41%) were missing at baseline, and n=26 (58%) were missing at the final assessment. Declining was the primary reason for non-completion for all outcomes across all time points.

Secondary outcomes

Summary falls data are presented in supplementary material 6. The crude falls incident rate ratio (IRR) was 1.95 (95% CI 0.63 to 7.18), suggestive of an almost two-fold increased incidence of falls within the usual care group.

Exercise capacity was maintained in the exercise group, but deteriorated in the usual care group, resulting in an overall difference of 36m (95% CI -12 to 84) in ISWT results and 181 seconds (95% CI -92 to 453) in EWST time. The time taken to complete the STS5 also increased in the usual care group (suggesting a deterioration in function), but was maintained in the exercise group, resulting in an overall difference of 5 seconds (95% CI -4 to 15) (supplementary material 7).

Step count increased in the exercise group resulting in an overall difference of 859 steps/day (95%CI -825 to 2543) on HD days and 888 steps/day (95%CI -84 to 1861) on non-HD days. Whilst sedentary time was increased in the exercise group on all days compared with the usual care group, this appeared to be offset by increases in light PA and moderate PA, and maintenance (albeit of low levels) of vigorous PA versus maintenance or deterioration across the same metrics in the usual care group (supplementary material 8). For PROMs, outcomes were largely unchanged, except for the DASI score, which appeared to deteriorate in the exercise group and increase in the usual care group, resulting in an overall difference in score

For peer review only - http://bmjopen.bmj.com/site/about/guidelines.xhtml

of 4.93 (95% CI -0.94 to 10.80) and the mental component summary score of the SF12 which improved in the usual care group, resulting in an overall difference in score of 4 (95% CI -3 to 10). Exercisers appeared to have a greater perception of the benefits of exercise compared with those in the control group (3, 95% CI -4 to 11) (supplementary material 9).

Serious adverse events

In total, n=13 (25%) experienced an SAE during the feasibility study, n=8 (33%) in the exercise group and n=5 (19%) in the usual care group. The most common reasons for SAEs were vascular access complications (n=3, 17%), stroke (n=3, 17%), acute coronary syndrome (n=2, 11%) and non-specific chest pain (n=2, 11%). All events were classed as serious as they resulted in hospitalisation. All resolved, and none were directly related to the intervention or trial.

Qualitative findings

Thirty-seven patients were approached for the qualitative study. Twenty-six were recruited and one died prior to data collection. Thirteen had participated in the feasibility trial. Nine received dialysis during shifts randomised to exercise, and four randomised to usual care. Twelve participants had declined to take part in the feasibility trial. Full characteristics for the qualitative sample are provided in supplementary material 10.

In addition to categories relating to the feasibility outcomes, categories relating to both the delivery and the characteristics of a tailored exercise intervention were identified. These are presented alongside illustrative quotes within Tables 3, 4, 5 and 6 and Figure 2.

For peer review only - http://bmjopen.bmj.com/site/about/guidelines.xhtml

Feasibility and acceptability of a definitive trial

Eligibility and recruitment

Declining to participate was underpinned by a perception that the trial could worsen overall health, particularly amongst those who had not previously participated in research or had recently commenced HD. Female participants believed that exercise was predominantly for men and that they were already doing enough daily activity, whilst participants living with moderate to severe frailty viewed ageing as an inevitable decline unlikely to be influenced by exercise. Motivators included a sense of altruism, and the perception that participation could provide opportunities to improve individual outcomes; learn about their own health; and access better healthcare. Participants felt that recruitment could be enhanced by the effective use of non-verbal communication, rapport building, adaptation to study documentation and actively involving family members in the recruitment process, as family support was often a prerequisite to participation (Table 3).

Trial retention

The primary reasons for withdrawal were becoming unwell, the duration of the trial and the research not meeting participants expectations. Participants suggested that having a rapport and maintaining regular dialogue with the research team might help retain participants within a future trial (Table 3).

For peer review only - http://bmjopen.bmj.com/site/about/guidelines.xhtml

Table 3. Categories relating to trial eligibility, recruitment and retention with illustrative quotes.

**Eligibility and recruitment**	
Challenges to recruitment	• [Interviewer]:" Have you ever taken part in any research before?" [Participant]: "No. I have not been asked really" (Female, moderately frail).<br>• "If anything happens I am in trouble, I would rather avoid it [research]" (Male, severely frail).<br>• "I don't think I had been dialysing all that long and I didn't know how [the trial] would affect me" (Male, mildly frail).<br>• "I do enough, I am always out, up-down, do this, do that, I have just put clothes in the machine you know for a wash, I go for a shower you know" (Female, mildly frail).<br>• "You have got to take age into consideration. Now I am getting old and there is a limit to what I can do. And it doesn't get any easier it gets worse" (Female, moderately frail)
Motivators to participation	• "If it helps someone else who has the same problem as me, they might be able to do something for him that they couldn't do for me" (Male, moderately frail).<br>• "I found the [outcome measures] very beneficial actually…it kind of educated me at the time…educationally it was informative" (Male, vulnerable).<br>• "What I like about research is that you are better looked after. I think if patients were a bit more aware that you are going to get preferential treatment, I think it would make it more attractive" (Female, vulnerable).
Suggested methods of enhancing recruitment	• "The research team should be there and explain that they don't want much, explain the benefits. Explain it's not for us [the research team] it's for the patients benefit, let them try and if then it doesn't go well [the participant] can stop it… it's not the information you give but talking as a person that's more important" (Male, vulnerable).<br>• "If I have got confidence in [the researcher] and that [they] know what they are doing and why, then it's fine" (Male, mildly frail).<br>• "I don't like it [the text] is too tiny, I can't even read [the information sheet] with reading glasses on…a picture or two might also help" (Female, mildly frail).<br>• "There's a lot of sheets in [the information sheet], I think people will get fed up reading all that" (Female, mildly frail)
**Trial retention**	
• "I have thought of dropping out because I am unable to do much. I am not interested because…I am not well. I have got a lot of things [wrong] with my body" (Female, mildly frail).<br>• "Somebody recently asked me about research and I tried it for about three weeks and I said no, not for me…I thought no, this is not what I want, it's not particularly helpful" (Female, moderately frail).	

For peer review only - http://bmjopen.bmj.com/site/about/guidelines.xhtml

The acceptability of IDC

IDC was generally perceived to be a safe and positive use of HD treatment time. However, it was also described as limited in scope, and participants were uncertain of its impact, particularly upon mobility, symptoms and falls (Table 4).

Table 4. Categories relating to the acceptability of IDC and illustrative quotes.

A safe and positive use of HD treatment time	• "Yes, I found it useful. It made me do some exercise instead of just laying here drinking tea and watching TV, doing jigsaw puzzles." (Male, mildly frail).<br>• "They bring the bike but first they test you…whether you're safe to do it and all that." (Female, mildly frail).
Limited scope and uncertain impact of IDC	• "We did cycling, and that was no choice because that's the only exercise we can do with our legs. You can't do sit-ups or stand-ups while you are lying down because you've got this thing [HD] going on" (Male, moderately frail)<br>• "I thought maybe it helps, I get rid of some problems or maybe you know I am not walking too much…so I say maybe if I do start cycling…you know I can walk…but nothing happened, no nothing." (Male, severely frail).<br>• "My legs have become stronger, they were wobbly…it's more sturdy now than before. Yet I still have the falls, that I cannot help. But my legs are stronger than they were. I am a bit more agile than I used to be." (Male, moderately frail).<br>• "It was fine, it was ok, I got on with it. I used to have a laugh but then eventually my knees were just so painful then my [blood] pressure played up a bit." (Female, vulnerable).<br>• "Blood pressure was coming down. Now I used to take medication for the blood pressure now I don't take it." (Male, vulnerable).

Outcome acceptability

As indicated by participant quotations in Table 5, the number of outcomes and follow-ups needed to be reduced and participants had a strong preference for outcomes that could be collected during HD treatment. Many found the ISWT and STS60 assessments too challenging. Participants were occasionally uncertain of the purpose of the questionnaires and

For peer review only - http://bmjopen.bmj.com/site/about/guidelines.xhtml

many reported difficulty quantifying symptom severity or a desire to provide 'anticipated' responses.

Maintaining mobility, and the ability to undertake a range of ADLS and social roles were viewed as key outcomes for a future trial. Only thirteen (52%) participants in the qualitative study agreed to complete a falls diary and many reported they preferred falls information to be collected during HD treatment. The majority who had fallen rarely reported them to healthcare professionals, believing that they were an expected consequence of HD or having had experience of their concerns about falls being overlooked. Consequently, falls prevention was not viewed as a key outcome.

For peer review only

Table 5. Categories relating to outcome acceptability and illustrative quotes.

Perceptions of outcome assessments		• “It was a bit of a task, too many [outcomes] personally” (Male, mildly frail).<br>• “It’s really helpful if it’s [outcome assessment] done here whilst I am on dialysis. We have got all this free time. Sometimes its five medical appointments a week, Tuesdays and Thursdays [non-dialysis days] become quite precious to me” (Male, vulnerable).<br>• “Do you mean someone would come to my house and do it [complete the functional tests]? I think that would be more doable.” (Female, moderately frail).<br>• “The walking ones [tests] I could make the distance, but the time was ridiculous, they asked me to do it fast. I can't, I have only got one speed” (Male, vulnerable).<br>• “I am not very good at scores, or you know, what they say about pain, what number it is? I am no good at that. I don't know what it means. I know it really hurts but I just can't describe the extent of it. It’s difficult to put it in a number like that” (Female, vulnerable).<br>• “Like all form filling, you can be undecided as to what or how to answer them. Sometimes you don’t, you kind of guess what you should be saying” (Male, mildly frail).
Important outcomes	Maintaining mobility	• “If you are walking better you are not getting out of breath and that's what does me. I mean I can't walk down this corridor to the ambulance because I am having to stop and get my breath back” (Female, moderately frail).
	Maintaining activities of daily living and social roles	• “I don't want to walk miles I just want to do enough to get around…from my chair to my commode or from my commode onto the bed. The only way I can do that is with the rotunda at the minute. I would like to do it with my walking frame” (Female, moderately frail).<br>• “I just want to carry on living and enjoying my life with my [partner] and children, my sisters, and of course all my friends, the church involvement, because I want to enjoy that for absolutely as long as I can” (Male, mildly frail).
	Falls and falls diaries	• “I don’t fall on a weekly basis… falling over is not something that happens on any sort of regular basis” (Male, moderately frail).<br>• “When I was at the hospital, I told them I had a fall. They don't want to know. They said, ‘you are perfect, your levels [bloods] are perfect and everything’.” (Male, vulnerable).<br>• “You know I sometimes I forget [to write in the diary]. So, the first days I had written and then I forgot it. And when you forget it then you can’t get the information right.” (Male, severely frail).<br>• “I can’t hold a pen properly, so I am not able to write. [Because of] arthritis they said, because I have got neuropathy and because I am on dialysis phosphate is causing my fingers to sometimes…close up.” (Male, moderately frail).<br>• “If [the researcher is] opposite you and gives you the information, [they’re] going to explain it even better, you know [they] can even ask [the participant] what happened and then they explain to [the researcher] different. But you forget you know the diary it’s very difficult and some of [the participants] won’t ever to know how to use it” (Male, mildly frail).

For peer review only - http://bmjopen.bmj.com/site/about/guidelines.xhtml

Perceptions of a tailored exercise programme

Delivery

There was no universally acceptable setting for exercise delivery (Table 6). Vulnerable and mildly frail participants (CFS 4-5) were particularly open to group-based exercise in the community or gym, which they felt would provide motivation through camaraderie with others. However, access barriers due to HD treatment, complex health needs, and lack of transport were common. Participants also described feeling self-conscious exercising amongst ‘normal’ people. Home-based exercise was preferred by those with moderate to severe frailty (CFS 6-7) due to easier access, greater flexibility and relevance to their daily activities. Despite this, concerns about lack of space and safety were highlighted by those who lived alone, whilst those with family were concerned about overburdening or injuring them by asking for support.

For peer review only - http://bmjopen.bmj.com/site/about/guidelines.xhtml

Table 6. Participants perceptions of the facilitators and barriers to group and home-based exercise.

Exercise setting	Facilitators	Barriers
Group community or gym-based exercise	"There is something about the group dynamics, when you try and do it on your own and you can't really focus. It's just so much easier to do as a group than an individual, especially if you have got motivational problems and you're having to do this [dialysis]" (Male, vulnerable)<br><br>"Better to be in a group, because when you see other people doing it, you just automatically join in and you feel like she can do it why not me?"(Female, mildly frail).<br><br>"We are all in the same boat. You can say how are you going on this week, you know you are on dialysis, are you finding this OK and you can get notes from them" (Female, severely frail).	"I was lucky enough that my wife was off so she took me and brought me, otherwise transport was a problem, sometimes I used to take a taxi because hospital transport you can't trust it" (Male, moderately frail).<br><br>"I have only got Tuesday and Thursday and most of the days that cropped up [to attend a falls prevention programme] they are either on a Wednesday or a Friday when I couldn't go because I have dialysis" (Male, mildly frail).<br><br>"Apparently because of my complex problems and disabilities he [participants GP] doesn't think anyone at the gym is sufficiently qualified to tell me which exercises are best" (Female, moderately frail).<br><br>"I would love to go to the gym and start sorting myself, but it's just a normal gym where normal keep-fit people go, so I have never ended up there" (Female, vulnerable).
Home-based	"When you are at home exercise is normal it really is. If you are going upstairs to get something you don't think…I am not going up there to get that. You go upstairs and get it because that's part of your everyday life" (Male, moderately frail).	"It's just the room that you have got where you can do exercise…if you haven't got that it's very difficult" (Female, moderately frail).<br><br>"I can't do anything in the home. There is no-one there, I'm alone, what if anything happens?" (Male, mildly frail).<br><br>"I am nervous about practising at home because if I couldn't get up, I don't want my husband hurting his back. I shall have to wait until a friend comes around and they could both help me" (Female, moderately frail).

For peer review only - http://bmjopen.bmj.com/site/about/guidelines.xhtml

Characteristics of a tailored exercise programme

Irrespective of the setting for delivery, participants identified several key features of a tailored exercise intervention which are summarised in Figure 2.

[FIGURE TWO. THE CORE COMPONENTS OF AN ACCEPTABLE EXERCISE PROGRAMME FOR PEOPLE LIVING WITH FRAILTY AND RECEIVING HAEMODIALYSIS TO BE INSERTED HERE]

Preparation

Participants lived with a range of debilitating symptoms, most frequently fatigue, pain and dyspnoea. Often daily activity alone was felt to be enough of a challenge. Common impacts of exercise (for example breathlessness whilst exercising) were interpreted as worsening symptoms or damage, and many participants were uncertain if exercise would be suitable or beneficial. They indicated that the reason for exercising needed to be sufficiently compelling. They wanted to know what to expect prior to exercising, and individualised goal setting was advocated to build motivation and appreciate improvements.

Content

Key components described were whole body resistance, aerobic and balance training. Many participants described being unable to get up once they had fallen and felt that practising this was also important. Routine physical activity was viewed as more purposeful than structured exercise ‘for the sake of it’ and participants spoke of their enjoyment of being outside and engaging in meaningful and physically active hobbies.

For peer review only - http://bmjopen.bmj.com/site/about/guidelines.xhtml

Structure

Supervision was viewed as essential to select, teach and progress exercises. Individual tailoring which considered the impact of disability, comorbidities and fluctuating symptoms was important, and a choice of exercises, for example swimming, dancing and yoga, was associated with increased enjoyment and engagement. Moderate to severely frail participants wanted the programme to be progressed in a supportive and collaborative manner. Those who were vulnerable or mildly frail wanted to be 'pushed' and progressed in a more assertive manner.

Having a companion (typically peers, family or friends) was viewed as helping to overcome access barriers and provide socialisation and mutual motivation. The sharing of experience was also seen as a powerful means of challenging preconceptions about exercise ability, although participants with moderate to severe frailty raised concerns about feeling embarrassed or 'judged' if they were less able.

Integrated mixed-methods analyses

The integrated qualitative and quantitative findings suggest that an RCT of IDC is feasible for frail HD patients following adaptation. However, IDC should not be the only intervention offered and the development of a multicomponent programme is warranted (Supplementary material 11).

DISCUSSION

For peer review only - http://bmjopen.bmj.com/site/about/guidelines.xhtml

These results suggest that an RCT of IDC is feasible for frail HD patients with adaptation to increase outcome acceptability and eligibility rates. Adherence to IDC was high and it was viewed as a safe and efficient use of HD treatment time. Secondary outcomes also suggest that, for HD patients with a CFS of 4-7, IDC may mitigate deterioration in exercise capacity, endurance and functional muscle strength and increase PA behaviour (steps/day), and reduce falls incidence. Despite this, participants described a preference for a multi-component programme that prepared them for exercise, offered variety, companionship and individualised supervision. No single preferred environment for the delivery of this intervention was identified, but appeared to be influenced by frailty grade and individual factors.

27% to 89% of secondary outcome measure data were missing, and, overall, this progression criterion was not achieved. Given that secondary measures are often insufficiently powered, reducing the number collected within a future trial may improve completion[26]. Falls were not of primary importance to participants, and this aligns with SONG-HD data which did not identify falls as a key outcome.[27] Our findings suggest that accurately capturing prospective falls data may be challenging due to under-reporting, and yet, retrospective falls data collection does not fully reflect the incidence and impact of falls, particularly those which do not require an ED visit or hospital admission. Given the high incidence of falls in this population, capturing falls data may be important in a future trial, and regular prospective recording of information relating to falls as a part of routine practice at the dialysis unit is recommended, in line with participant feedback.[5] This would provide both clinicians and researchers with higher quality data for use in both prospective and retrospective studies, and to inform clinical care.

For peer review only - http://bmjopen.bmj.com/site/about/guidelines.xhtml

Further exploration and validation of meaningful measures for HD patients living with frailty is also warranted. Some of the functional measures (the STS60 and ISWT) included were too challenging. In the absence of a core set of functional outcome measures for older people, or people receiving haemodialysis, we suggest that the SPPB may be the most appropriate and feasible method of capturing information about mobility and function. Although challenges with ceiling effects have been identified, this measure had the lowest levels of non-completion within this study, and has demonstrated good test-retest reliability in HD patients and excellent validity and responsiveness to change following an intervention in older adults. [28,29] To date, measures of basic and instrumental ADL ability and participation have rarely been used in exercise studies in an HD population. These outcomes were, however, highlighted as important within this study, and have also been included in guidelines and core outcomes sets for HD and older people, warranting their inclusion in future exercise studies relating to frail HD populations.[27,30,31]

The results of this study indicate that changes to eligibility criteria and screening processes are required. As only patient participants were interviewed, it was not possible to gain any insight on this aspect of feasibility from the qualitative component. Importantly, the challenges of identifying eligible participants do not appear to be unique to this studyand a multicentre trial may be required.[32]

Higher proportions of older, female and more severely frail HD patients declined to participate and the qualitative data indicated this was due to negative perceptions relating to participation in both exercise and research. Such findings clearly have implications for the external validity

For peer review only - http://bmjopen.bmj.com/site/about/guidelines.xhtml

of a future trial and the reach of the intervention at the point of implementation.[24] To address this, this study suggests recruitment strategies which utilise effective non-verbal communication skills to build rapport and explore participants' perceptions of the intervention and the research process, and subsequently provide balanced information about the study, may lead to more representative recruitment. A sense of equipoise may be preserved by emphasising altruism, access to potentially enhanced care, and an opportunity to learn about their health (which were all identified as motivators to participation), rather than the potential individual benefits of the intervention itself. Involving families and/or peer supporters who have experience of the study and intervention in the recruitment process and introducing opportunities for participants to observe the exercise intervention may also be beneficial. Ultimately the selection of these strategies will depend upon the resources available and the need to strike a balance between conducting a trial with high internal and external validity and going beyond what is pragmatically possible to engage patients in the intervention at the implementation phase.

This study suggests that IDC may reduce the incidence of falls resulting in ED visits and hospital admissions in frail HD patients potentially by attenuating a decline in exercise capacity, physical activity behaviour and function at levels shown to be clinically meaningful in other long-term conditions.[33,34] This indicates that preventing deterioration may be as valuable, and more attainable, as improving outcomes in a frail population. Despite this, frail participants experienced difficulties achieving the proposed level of exercise and maintaining motivation in the face of varying symptomology. Exercise programmes have a dose-response, and these factors may have reduced participants physical capability to exercise and achieve optimal benefit, despite the overall good level of adherence. Clinical decision support tools have been used in other populations to rationalise exercise prescription, progression and

For peer review only - http://bmjopen.bmj.com/site/about/guidelines.xhtml

amendment in the presence of varying symptomology, and a similar approach may be beneficial for frail HD patients.[35]

This study indicates that participants desire a multicomponent exercise programme, and require an intervention that addresses their particularly low levels of PA. Whilst step count and time spent in light and moderate PA increased following IDC, these were below PA recommendations for older people.[36] To date, PA interventions for HD patients have predominantly centred around walking, which may not be appropriate for those living with frailty.[37-40] This study suggests that functional training (task-orientated exercise which engages multiple muscle groups) and physical activity that focuses on 'doing more' of these usual tasks may be more acceptable and efficacious. To date, two studies have employed similar approaches with non-frail HD patients. One study demonstrated significant improvements in lower extremity performance and the other a non-significant improvement in physical function and maintenance of other SF-36 domains compared with the control group[41,42]. In older people without CKD who are living with frailty, functional training included as part of a multicomponent exercise programme is beneficial across a range of outcomes, including greater ability to rise from the floor following a fall.[40,43-46] A similar approach to exercise prescription may be warranted in a frail HD population.

Numerous barriers and facilitators to exercise were identified within this study, which have implications for the design of a programme. The use of theory is crucial in the development of effective interventions and the behaviour change wheel (BCW) is most frequently cited in the development of interventions in CKD. [47] Mapping the identified barriers and facilitators to the BCW indicates that ameliorating symptom burden prior to exercise, individualised

For peer review only - http://bmjopen.bmj.com/site/about/guidelines.xhtml

exercise counselling, and a collaborative, problem-solving approach to exercise education are most likely to encourage and sustain participation.[47,48] Devising ways in which peer and family involvement can be incorporated into the programme may also increase motivation and opportunity to exercise but should be carefully managed given the potential for negative comparison amongst the frailest patients.

A lack of preferred environment for intervention delivery may have implications for a definitive RCT. Exercise interventions require motivation, and limited engagement may negatively influence a trials external and internal validity. Ignoring patient preference is also out of step with clinical practice, where rehabilitation involves shared decision-making. Taken together, these factors have implications for determining treatment effects and future intervention implementation.[49] There is increasing recognition that novel trial designs may be indicated when evaluating complex interventions and a Partially Randomised Patient Preference Trial, where participants without preference are randomised whilst those with a preference receive their choice, would provide information on both the efficacy of the intervention and the influence of preference.[49,50]

Strengths and limitations

To our knowledge, this study is the first to examine the feasibility of an RCT of IDC for frail HD patients and to explore how trial procedures and exercise programmes should be specifically tailored to the needs of this group, from their own perspectives. Key strengths were the use of a validated frailty risk-stratification measure and multiple qualitative methods which provided a form of triangulation.[51] There were, however, challenges to recruiting severely frail participants, and those from a more diverse range of black and minority ethnic

For peer review only - http://bmjopen.bmj.com/site/about/guidelines.xhtml

groups, to both the trial and the qualitative study. Additionally, the views of clinicians and researchers were not explored. A future RCT should also blind outcome assessors to group allocation to reduce the potential for detection bias. Finally, this study is exploratory and therefore all secondary measures of exercise capacity, function and PROMS should be interpreted with caution, not least due to the high number of participants who did not complete the follow up tests.

Conclusion

In summary, this study suggests that a future definitive trial of IDC is feasible within a HD population with a CFS of 4-7 and paying particular attention in the design to those factors mentioned above may facilitate improved rates of eligibility and outcome completion. Outcomes focusing on independence and participation should be the primary outcomes of interest in a future trial. Whilst an exploratory analysis suggests some potential benefits to IDC, a tailored intervention comprising a comprehensive multi-component exercise programme, symptom management, education and behaviour change is better suited to frail HD patients' needs.

ACKNOWLEDGEMENTS

The authors would like to thank the staff and patients at all units, Fresenius Medical Care and The Leicester Kidney Care Appeal. The authors also wish to thank Freya Tyrer for sharing her statistical expertise.

COMPETING INTERESTS

For peer review only

The authors have no conflicts of interest to declare.

FUNDING

The work was funded by the National Institute for Health Research (NIHR) Leicester Biomedical Research Centre, Collaboration for Leadership in Applied Health Research and Care East Midlands (CLAHRC EM) and partly funded by the Stoneygate Trust. H.M.L.Y. and J.O.B. are supported by grants from the NIHR (DRF-2016-09-015 and CS-2013-13-014). S.J.S. is supported by the Collaboration for Leadership in Applied Health Research and Care East Midlands. The views expressed in this publication are those of the authors and not necessarily those of the NHS, the NIHR or the Department of Health and Social Care. The funders had no role in the study design; collection, analysis, and interpretation of the data;

AUTHORS CONTRIBUTIONS

Literature search: HMLY; Research idea and study design: HMLY, JOB; participant recruitment: HMLY, DS, PH, DC, MPMGB, JOB, CG; data acquisition: HMLY, DS,PH,DC, MPMGB,CG WJ, MC; clinical governance: JOB; data analysis: HMLY, SG; statistical analysis: HMLY; supervision and mentorship: SC, HE, SG, SJS, ACS, JOB; manuscript preparation: HMLY; reviewed final manuscript: all. Each author contributed important intellectual content during manuscript drafting or revision and accepts accountability for the overall work by ensuring that questions pertaining to the accuracy or integrity of any portion of the work are appropriately investigated and resolved. All authors have read and approved the final version

For peer review only

For peer review only - http://bmjopen.bmj.com/site/about/guidelines.xhtml

615 **ETHICAL APPROVAL**

616 This study was approved by the East Midlands (Northampton; REC ref: 14/EM/1190) and
617 South West (Bristol; REC ref: 17/SW/0048) NHS Research Ethics Committees for the trial
618 and the qualitative component respectively

619

620 **DATA SHARING STATEMENT**

621 The datasets used and analysed during the current study are available from the corresponding
622 author on reasonable request.

For peer review only

For peer review only - http://bmjopen.bmj.com/site/about/guidelines.xhtml

REFERENCES

1. Clegg A, Young J, Iliffe S, et al. Frailty in elderly people. *The Lancet*. 2013;381(9868):752-762.

2. Zhao Y, Liu Q, Ji J. The prevalence of frailty in patients on hemodialysis: A systematic review and meta-analysis. *Int Urol Nephrol*. 2019:1-6.

3. Iyasere OU, Brown EA, Johansson L, et al. Quality of life and physical function in older patients on dialysis: A comparison of assisted peritoneal dialysis with hemodialysis. *Clin J Am Soc Nephrol*. 2016;11(3):423-430.

4. Johansen KL, Chertow GM, Jin C, et al. Significance of frailty among dialysis patients. *Journal of the American Society of Nephrology*. 2007;18(11):2960-2967.

5. López-Soto PJ, De Giorgi A, Senno E, et al. Renal disease and accidental falls: A review of published evidence. *BMC nephrology*. 2015;16(1):176.

6. Johansen KL, Dalrymple LS, Delgado C, et al. Factors associated with frailty and its trajectory among patients on hemodialysis. *Clinical Journal of the American Society of Nephrology*. 2017;12(7):1100-1108.

7. Wang CJ, Johansen KL. Are dialysis patients too frail to exercise? Seminars in dialysis.2019;32(4) 1-6.

8. Young HM, March DS, Graham-Brown MP, et al. Effects of intradialytic cycling exercise on exercise capacity, quality of life, physical function and cardiovascular measures in adult haemodialysis patients: A systematic review and meta-analysis. *Nephrology Dialysis Transplantation*. 2018; 33(8),1436-1445.

For peer review only - http://bmjopen.bmj.com/site/about/guidelines.xhtml

9. Brown EA. Can quality of life be improved for the increasing numbers of older patients with end-stage kidney disease? *Expert review of pharmacoeconomics & outcomes research*. 2010;10(6):661-666.

10. Farrington K, Covic A, Nistor I, et al. Clinical practice guideline on management of older patients with chronic kidney disease stage 3b or higher (eGFR< 45 mL/min/1.73 m2): A summary document from the european renal best practice group. *Nephrology Dialysis Transplantation*. 2017;32(1):9-16.

11. Graham-Brown M, March DS, Churchward DR, et al. Design and methods of CYCLE-HD: Improving cardiovascular health in patients with end stage renal disease using a structured programme of exercise: A randomised control trial. *BMC nephrology*. 2016;17(1):69.

12. Charmaz K. Constructing grounded theory (introducing qualitative methods series). 2nd edn. London: Sage Publications 2014.

13. Nixon AC, Bampouras TM, Pendleton N, et al. Diagnostic accuracy of frailty screening methods in advanced chronic kidney disease. *Nephron*. 2019;141(3):147-155.

14. Rockwood K, Song X, MacKnight C, et al. A global clinical measure of fitness and frailty in elderly people. *CMAJ*. 2005;173(5):489-495.

15. Alfaadhel TA, Soroka SD, Kiberd BA, et al. Frailty and mortality in dialysis: Evaluation of a clinical frailty scale. *Clin J Am Soc Nephrol*. 2015;10(5):832-840.

16. Hoffmann TC, Glasziou PP, Boutron I, et al. Better reporting of interventions: Template for intervention description and replication (TIDieR) checklist and guide. *BMJ*. 2014;348:g1687.

For peer review only - http://bmjopen.bmj.com/site/about/guidelines.xhtml

666 17. Borg GA. Psychophysical bases of perceived exertion. *Med Sci Sports Exerc*. 1982;14(5):377-381.

668 18. Sim J, Lewis M. The size of a pilot study for a clinical trial should be calculated in relation to considerations of precision and efficiency. *J Clin Epidemiol*. 2012;65(3):301-308.

670 19. Young HML, Goodliffe S, Madhani M, et al. Co-producing progression criteria for feasibility studies: A partnership between patient contributors, clinicians and researchers. *International journal of environmental research and public health*. 2019;16(19):3756.

673 20. Avery KN, Williamson PR, Gamble C, et al. Informing efficient randomised controlled trials: Exploration of challenges in developing progression criteria for internal pilot studies. *BMJ open*. 2017;7(2):e013537.

676 21. Beddhu S, Bruns FJ, Saul M, et al. A simple comorbidity scale predicts clinical outcomes and costs in dialysis patients. *Am J Med*. 2000;108(8):609-613.

678 22. Lamb SE, Jørstad-Stein EC, Hauer K, et al. Development of a common outcome data set for fall injury prevention trials: The prevention of falls network europe consensus. *J Am Geriatr Soc*. 2005;53(9):1618-1622.

681 23. Young HML, Orme MW, Song Y, et al. Standardising the measurement of physical activity in people receiving haemodialysis: Considerations for research and practice. *BMC nephrology*. 2019;20(1):450.

684 24. Eldridge SM, Chan CL, Campbell MJ, et al. CONSORT 2010 statement: Extension to randomised pilot and feasibility trials. *Pilot and feasibility studies*. 2016;2(1):64.

For peer review only - http://bmjopen.bmj.com/site/about/guidelines.xhtml

25. Richards DA, Bazeley P, Borglin G, et al. Integrating quantitative and qualitative data and findings when undertaking randomised controlled trials. *BMJ open*. 2019;9(11).

26.Witham MD, Stott DJ. Conducting and reporting trials for older people. *Age Ageing*. 2017;46(6):889-894.

27. SONG initiative. Standardised outcomes in nephrology - haemodialysis. http://songinitiative.org/projects/song-hd/. Updated 2017. Accessed July, 2019.

28. Lamb SE, Keene DJ. Measuring physical capacity and performance in older people. *Best Practice & Research Clinical Rheumatology*. 2017;31(2):243-254.

29. de Villar LO, Martínez-Olmos FJ, Junqué-Jiménez A, et al. Test-retest reliability and minimal detectable change scores for the short physical performance battery, one-legged standing test and timed up and go test in patients undergoing hemodialysis. *PloS one*. 2018;13(8):e0201035.

30. Akpan A, Roberts C, Bandeen-Roche K, et al. Standard set of health outcome measures for older persons. *BMC geriatrics*. 2018;18(1):36.

31. Matsufuji S, Shoji T, Yano Y, et al. Effect of chair stand exercise on activity of daily living: A randomized controlled trial in hemodialysis patients. *Journal of Renal Nutrition*. 2015;25(1):17-24.

32. Ferrucci L, Guralnik JM, Studenski S, Fried LP, Cutler Jr GB, Walston JD, Interventions on Frailty Working Group. Designing randomized, controlled trials aimed at preventing or delaying functional decline and disability in frail, older persons: a consensus report. Journal of the American Geriatrics Society. 2004 Apr;52(4):625-34.

For peer review only - http://bmjopen.bmj.com/site/about/guidelines.xhtml

33. Yates T, Gray LJ, Henson J, et al. Impact of depression and anxiety on change to physical activity following a pragmatic diabetes prevention program within primary care: Pooled analysis from two randomized controlled trials. *Diabetes Care*. 2019;42(10):1847-1853.

34. Zatloukal J, Houchen-Wolloff L, Ward S, et al. Clinical significance for ESWT in COPD after a course of pulmonary rehabilitation. *Chro Respiratory Disease.*2015;46.1479973119853828

35. Carvalho LP, Kergoat M, Bolduc A, et al. A systematic approach for prescribing posthospitalization home-based physical activity for mobility in older adults: The PATH study. *Journal of the American Medical Directors Association*. 2019;20(10):1287-1293.

36. Department of Health. Physical activity guidelines for older adults. https://www.nhs.uk/live-well/exercise/physical-activity-guidelines-older-adults/. Updated 2019. Accessed April, 2020.

37. Baggetta R, D'Arrigo G, Torino C, et al. Effect of a home based, low intensity, physical exercise program in older adults dialysis patients: A secondary analysis of the EXCITE trial. *BMC geriatrics*. 2018;18(1):248.

38. Bohm C, Stewart K, Onyskie-Marcus J, Esliger D, et al. Effects of intradialytic cycling compared with pedometry on physical function in chronic outpatient hemodialysis: A prospective randomized trial. *Nephrology Dialysis Transplantation*. 2014;29(10):1947-1955.

39. Sheshadri A, Kittiskulnam P, Lazar AA, et al. A walking intervention to increase weekly steps in dialysis patients: A pilot randomized controlled trial. *American Journal of Kidney Diseases*. 2020; 75(4):488-496

For peer review only

For peer review only - http://bmjopen.bmj.com/site/about/guidelines.xhtml

40. de Labra C, Guimaraes-Pinheiro C, Maseda A, et al. Effects of physical exercise interventions in frail older adults: A systematic review of randomized controlled trials. *BMC geriatrics*. 2015;15(1):1.

41. Tawney KW, Tawney PJ, Hladik G, et al. The life readiness program: A physical rehabilitation program for patients on hemodialysis. *American journal of kidney diseases*. 2000;36(3):581-591.

42. Bogataj Š, Pajek J, Ponikvar JB, et al. Kinesiologist-guided functional exercise in addition to intradialytic cycling program in end-stage kidney disease patients: A randomised controlled trial. *Scientific reports*. 2020;10(1):1-10.

43. Hofmeyer MR, Alexander NB, Nyquist LV, et al. Floor-Rise strategy training in older adults. *J Am Geriatr Soc*. 2002;50(10):1702-1706.

44. Skelton DA, McLaughlin AW. Training functional ability in old age. *Physiotherapy*. 1996;82(3):159-167.

45. Giné-Garriga M, Roqué-Fíguls M, Coll-Planas L, et al. Physical exercise interventions for improving performance-based measures of physical function in community-dwelling, frail older adults: A systematic review and meta-analysis. *Arch Phys Med Rehabil*. 2014;95(4):753-769. e3.

46. Liu C, Shiroy DM, Jones LY, et al. Systematic review of functional training on muscle strength, physical functioning, and activities of daily living in older adults. *European review of aging and physical activity*. 2014;11(2):95.

For peer review only

47. Clarke AL Jhamb M Bennett P. Barriers and facilitators for engagement and implementation of exercise in end-stage kidney disease: Future theory-based interventions using the behavior change wheel. *Seminars in Dialysis*. 2019:1-12.

48. Michie S, Atkins L, West R. The behaviour change wheel. *A guide to designing interventions.1st ed.Great Britain: Silverback Publishing*. 2014:1003-1010.

49. Wasmann KA, Wijsman P, van Dieren S, et al. Partially randomised patient preference trials as an alternative design to randomised controlled trials: Systematic review and meta-analyses. *BMJ open*. 2019;9(10):e031151.

50. Craig P, Dieppe P, Macintyre S, et al. Developing and evaluating complex interventions: The new medical research council guidance. *BMJ*. 2008;337:a1655.

51. Tracy SJ. Qualitative quality: Eight “big-tent” criteria for excellent qualitative research. *Qualitative inquiry*. 2010;16(10):837-851.

Legends to Figures

Figure 1.CONSORT

Figure 2. The core components of an acceptable exercise programme for people living with frailty and receiving haemodialysis.

SUPPLEMENTARY MATERIAL

Supplementary material 1. Inclusion and exclusion criteria for the CYCLE-HD trial

Supplementary material 2. Summary of intervention characteristics, in line with TiDier guidance.

For peer review only

For peer review only - http://bmjopen.bmj.com/site/about/guidelines.xhtml

Supplementary material 3. *A priori* progression criteria based on the primary feasibility objectives.

Supplementary material 4. Patient-reported secondary outcome measures.

Supplementary material 5. Interview topic guide questions.

Supplementary material 6. Falls summary data and incidence of falls per person years.

Supplementary material 7. Changes in exercise capacity and physical function after six months.

Supplementary material 8. Changes in physical activity (accelerometry data) after six months.

Supplementary material 9. Patient-reported outcomes measures after six months.

Supplementary material 10. Baseline demographic and clinical characteristics for the qualitative participants.

Supplementary material 11. Joint display of quantitative and qualitative results, with an overall assessment of mixed-methods inferences.

For peer review only

For peer review only - http://bmjopen.bmj.com/site/about/guidelines.xhtml

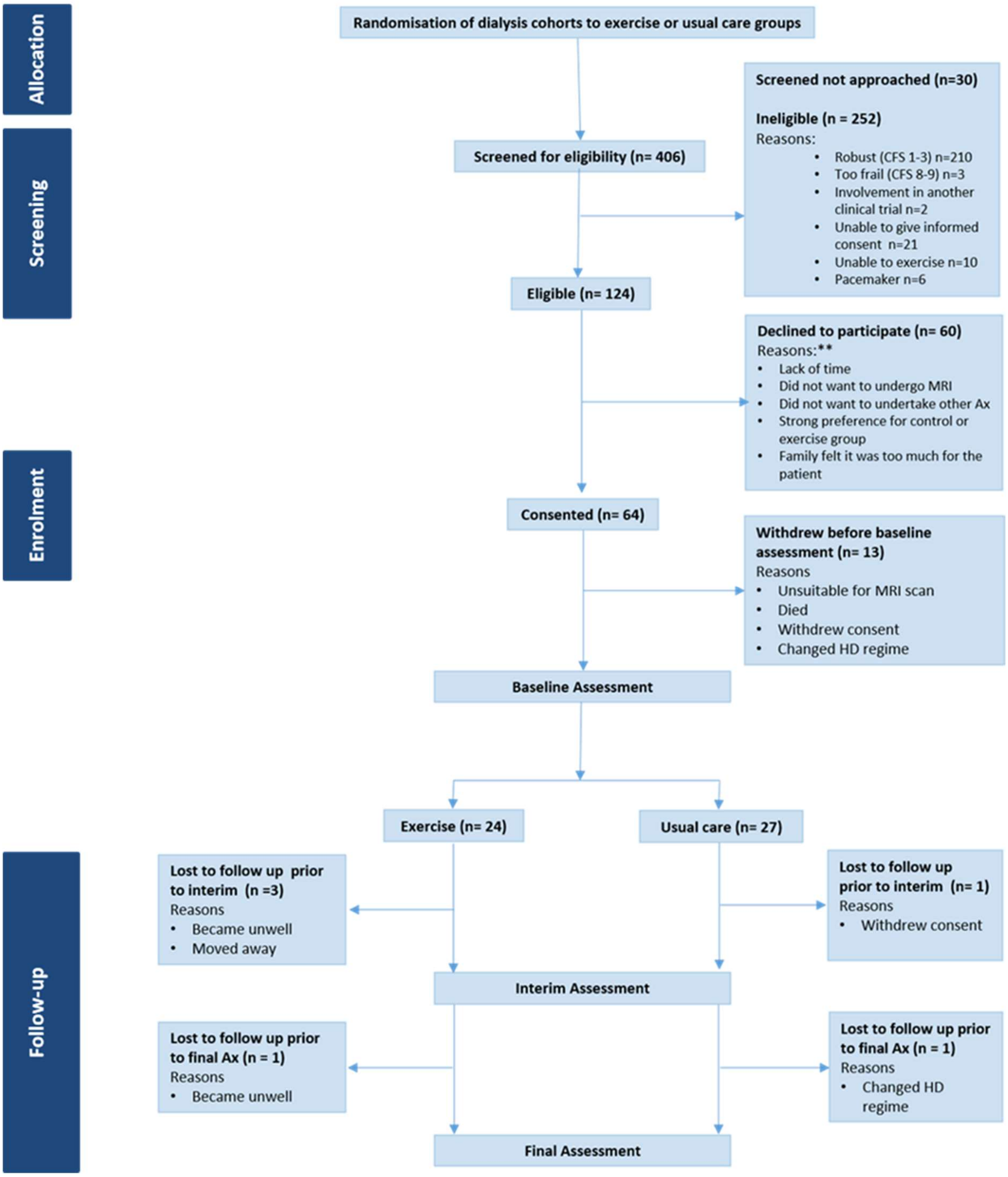

Abbreviations: Ax, assessment; CFS, Clinical Frailty Scale; HD, haemodialysis.

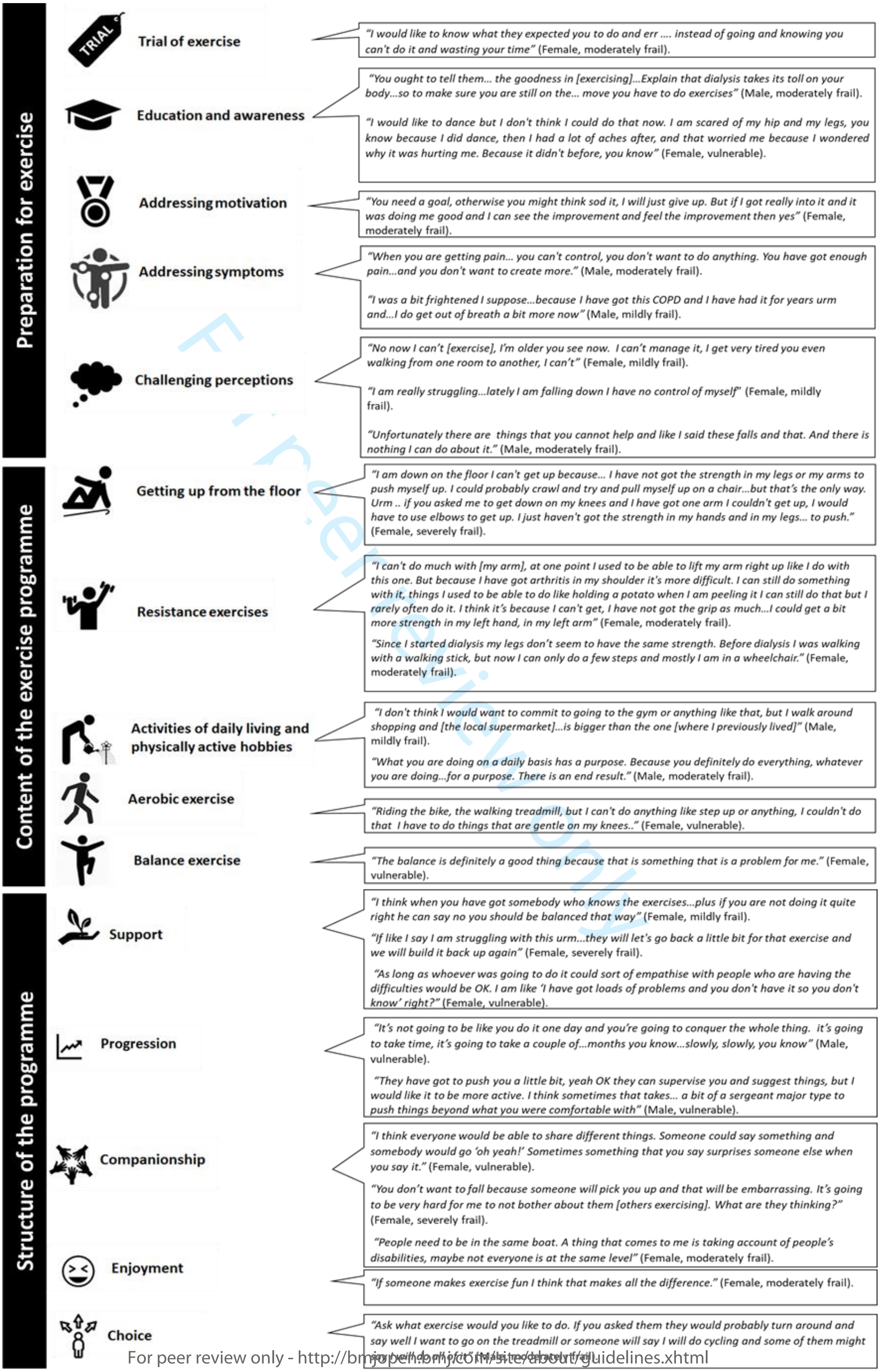

For peer review only

Supplementary material 1. Inclusion and exclusion criteria for the *CYCLE-HD* trial.

Inclusion criteria	Exclusion criteria
Prevalent HD patient (> three months)	Unable to participate in current exercise programme due to perceived physical or psychological barriers
Aged 18 years or older	Unable to undergo MRI scanning (metal implants, severe claustrophobia)
Able and willing to give informed consent	Unfit to undertake exercise according to the American College of Sports Medicine (ACSM) guidelines

For peer review only

Supplementary material 2. Summary of intervention characteristics, in line with TiDier guidance.

**Description of intervention.**		A structured, supervised cycling exercise intervention delivered during in-centre HD.
**Rationale.**		• Intradialytic cycling provides aerobic and low-level resistance training, is associated with increased adherence and is most widely used within practice.
**What.**	Materials provided to participants or used to support intervention delivery.	• Cycling was delivered using the Moto Med Letto 2 (Medimotion Ltd).<br>• Materials: individualised exercise prescription and records of individual training bouts (duration (mins), intensity (RPE), resistance (gear), power output (watts) and energy expenditure (Kcal).<br>• General information on the benefits of exercise (posters and leaflets) available across all 3 HD centres.
	Materials used to train intervention providers.	Standardised progression and training protocol used by all providers.
**Who** (intervention providers).		• Qualified exercise professionals with experience of delivering exercise to renal patients.<br>• All providers were directly involved in the study, and not delivering the sessions as part of a clinical role.<br>• Roles included exercise provision, supervision, monitoring and progression.
**How** (mode of delivery).		One to one, face to face.
**Where** (location).		Three HD units across the East Midlands, UK.
**When and how much**	The frequency of delivery.	Thrice weekly during each dialysis session.
	Target intensity of each bout of exercise.	RPE 12-14 (moderate intensity), cadence 60-70 RPM.
	Target duration of each bout of exercise.	At least 30 minutes of continuous exercise.
	The total duration of delivery.	Six months, with a one-month run-in period to achieve the target exercise prescription.
**Tailoring.**		• The starting resistance (gear) based on the individual's tolerance.<br>• RPE used throughout to monitor and progress the exercise.<br>• Interval training was permitted.

Abbreviations: HD, haemodialysis; Kcals, kilocalories; RPE, rating of perceived exertion, RPM, revolutions per minute.

For peer review only

Supplementary material 3. A priori progression criteria based on the primary feasibility objectives.

**Eligibility**	**Stop**	Less than **20%** of all patients eligible
	**Go**	More than **50%** of all patients eligible
**Recruitment**	**Stop**	Less than **25%** of eligible patients recruited
	**Go**	More than **50%** of eligible patients recruited
**Exercise acceptability**	**Stop**	Less than **30%** adherence to the exercise sessions
	**Go**	More than **70%** adherence to the exercise sessions
**Outcome acceptability**	**Stop**	Less than **70%** outcome measure completion
	**Go**	More than **80%** outcome measure completion
**Loss to follow-up**	**Stop**	More than **40%** loss to follow-up
	**Go**	Less than **20%** loss to follow-up

For peer review only

Supplementary material 4. Patient-reported secondary outcome measures.

**Patient-reported secondary outcome**	**Construct measured**
12-item Short-Form Health Survey Version 2 (SF-12)	Generic health-related quality of life. Higher scores reflect better HRQoL.Scores are presented as a mental and physical component summary score.
Palliative care Outcomes Scale – Renal version (POS-R)	Renal specific measure of symptomology and symptom burden. A global symptom score was calculated by totalling all the scored items within the questionnaire. The mean number of symptoms, symptom severity was also calculated. Higher scores reflect greater symptom burden.
Hospital Anxiety and Depression Scale (HADS)	Emotional distress. A score of ≥14 indicates the presence of emotional distress in HD patients
The Exercise Self-Efficacy Scale (ESES)	Exercise confidence. Higher scores reflecting greater self-efficacy.
Dialysis Patient-Perceived Exercise Benefits and Barriers Scale (DPPEBBS)	HD patients' perceptions of benefits and barriers to exercise. Higher scores indicate a greater perception of the benefits of exercise over barriers.
The Dukes Activity Status Index (DASI)	Self-reported physical function. Higher scores indicate higher levels of physical function. The questionnaire was also used to estimate VO_2 peak.

HD, haemodialysis; HRQoL, Health-related quality of life.

For peer review only

Supplementary material 5. Interview topic guide question.

Diary

1. Can you tell me about how you have been using the diary?

2. If we asked patients to keep diaries like yours as part of a future study, what might help them?

3. [If applicable] I’ve had an opportunity to have a look through your diary. Could you tell me more about…?

Exercise intervention for frailty and falls

4. For some people exercising helps to prevent falls, make people more able and feel better. How do you feel about exercising?

5. Cycling during dialysis is thought to be a good way to exercise if you are on dialysis. Have you seen these bikes?

6. Programmes that are available for other people who fall include things like group exercise and education. What do you think about this?

7. These programmes usually take place at the hospital. What do you think about this?

8. Some people prefer to do their exercise at home. What do you think about this?

9. Where do you think a programme should be run?

10. How often do you think you would be able to exercise?

11. Would you want any support to help you exercise?

12. What might put you off exercising?

13. What questions might you have before you decide to take part or not?

14. If you did take part in some kind of exercise programme, what improvements would you most like to see?

Research

15. Have you ever been involved in research before? [Could tailor to involvement in CYCLE study (declined/ took part. If took part completed/dropped out) if patient unsure]

16. What do you think about the information you receive when deciding to take part in a research study?

For peer review only

17. Often researchers ask you to complete some assessments or tests to see if the thing they are studying is effective or not. What do you think would help patients to complete these assessments/ tests?

18. Sometimes people don't complete the research study, which may happen for several reasons [give examples as needed]. What do you think would help keep dialysis from dropping out of research studies?

19. What would you like to happen once you reach the end of the study?

For peer review only

Supplementary material 6. Falls summary data and incidence of falls per person years.

	Usual care (n=27)	Exercise (n=24)
**Number of Falls**	11	5
**Number (% of group) of non-fallers**	19 (70)	20 (83)
**Number (% of group) fallers (≥ 1 fall)**	8 (30)	4 (17)
**Number (% of group) frequent fallers (≥2 falls)**	3 (11)	1 (4)
**Person years**	40.5	36
**Incidence rate**	0.27	0.14

For peer review only

Supplementary material 7. Changes in exercise capacity and physical function after six months.

	Outcome		Usual Care	Exercise	Difference (95% CI)
**ISWT** (m)		n	16	15	36 (-12 to 84)
		Baseline	184 ± 130	237 ± 173	
		Final	158 ± 154	248 ± 192	
		Change	-26 ± 68	11 ± 63	
**ESWT** (secs)		n	14	15	181 (-92 to 453)
		Baseline	347 ± 384	401 ± 375	
		Final	193 ± 304	428 ± 423	
		Change	-153 ± 286	27 ± 413	
**STS60** (n)		n	17	15	0 (-5 to 4)
		Baseline	10 ± 12	13 ± 11	
		Final	10 ±13	13 ± 12	
		Change	0 ± 7	0 ± 6	
**SPPB**	Total score	n	17	15	0.5 (-0.7 to 2)
		Baseline	7 ± 3	9 ± 3	
		Final	6 ± 2	8 ± 3	
		Change	-1 ± 2	-0.5 ± 1	
	4m walk time (secs)	n	17	15	1 (-1 to 4)
		Baseline	7 ± 6	4± 1	
		Final	6 ± 4	5 ± 2	
		Change	1 ± 5	0 ± 1	
	Gait speed (m/s)	n	17	15	0.05 (-0.12 to 0.22)
		Baseline	0.74 ± 0.29	0.96 ± 0.28	
		Final	0.74 ± 0.28	0.91 ± 0.31	
		Change	0.00 ± 0.22	-0.05 ± 0.24	
	STS5 (secs)	n	9	10	5 (-4 to 15)
		Baseline	17 ± 7	16 ± 14	
		Final	23 ± 13	16 ± 10	
		Change	6 ± 11	0 ± 8.	

Abbreviations: CI, confidence interval; ESWT, Endurance Shuttle Walk Test; ISWT, Incremental Shuttle Walk Test; m/s, metres per second; Secs, seconds; SPPB, Short Physical Performance Battery; STS5, Sit to Stand Five Repetitions; STS60, Sit to Stand in Sixty Seconds.

For peer review only

Supplementary material 8. Changes in physical activity (accelerometry data) after six months.

	Type of day		Usual Care	Exercise	Difference (95% CI)
**Waking wear time** (mins)	HD	n	5	10	244 (16 to 473)
		Baseline	891 ± 202	818 ± 183	
		Final	749 ± 105	921 ± 171	
		Change	-142 ±166	103 ± 204	
	Non-HD	n	5	10	170 (-13 to 353)
		Baseline	893 ± 90	927 ± 216	
		Final	817 ± 134	1022 ± 165	
		Change	-75 ± 201	95 ± 129	
**Steps** (steps/day)	HD	n	5	10	859 (-825 to 2543)
		Baseline	2252 ± 4210	1373 ± 1080	
		Final	2464 ± 4783	2444 ± 1904	
		Change	211 ± 593	1070 ± 1665	
	Non-HD	n	5	10	888 (-84 to 1861)
		Baseline	3076 ± 5790	2387 ± 1696	
		Final	2645 ± 5284	2845 ± 2117	
		Change	-430 ± 603	458 ± 903	
**Sedentary** (mins/ day)	HD	n	5	10	28 (-284 to 340)
		Baseline	954 ±338	954 ± 203	
		Final	965 ± 208	992 ± 182	
		Change	10 ± 200	38 ± 287	
	Non-HD	n	5	10	124 (-205 to 454)
		Baseline	1022 ± 357	1103 ± 253	
		Final	912 ± 224	1117 ± 174	
		Change	-110 ± 298	14 ± 269	
**Light PA** (mins/day)	HD	n	5	10	91 (23 to -158)
		Baseline	125 ± 51	83 ± 42	
		Final	79 ± 39	127 ± 73	
		Change	-46 ± 45	44 ± 62	
	Non-HD	n	5	10	9 (-71 to 91)
		Baseline	145 ± 59	133 ± 50	
		Final	154 ± 99	151 ± 59	
		Change	9 ± 108	18 ± 44	
**Moderate PA** (mins/day)	HD	n	5	10	13 (-32 to 57)
		Baseline	83 ± 105	29 ± 33	
		Final	85 ± 123	43 ± 55	
		Change	1 ± 52	14 ± 29	
	Non-HD	n	5	10	20 (40 to -79)
		Baseline	79 ± 96	46 ± 61	
		Final	75 ± 112	62 ± 105	
		Change	-4 ± 40	16 ± 55	
**Vigorous PA** (mins/day)	HD	n	5	10	3 (-1 to 8)
		Baseline	4 ± 9	1 ± 1	
		Final	1 ± 2	1 ± 3	
		Change	-3 ± 7	0 ± 2	
	Non-HD	n	5	10	1 (0 to 2)
		Baseline	3 ± 0	1 ± 4	
		Final	2 ± 5	1 ± 4	
		Change	-1 ± 2	0 ± 0	

Abbreviations: CI, confidence interval; HD, haemodialysis; mins, minutes; PA, physical activity.

For peer review only

Supplementary material 9. Patient-reported outcomes measures after six months.

	Outcome		Usual Care	Exercise	Difference (95% CI)
**SF-12**	PCS	n	19	19	0 (-4 to 5)
		Baseline	35 ± 9	35 ± 10	
		Final	36 ± 10	36 ± 10	
		Change	1 ± 7	1 ± 7	
	MCS	n	19	19	4 (-3 to 10)
		Baseline	43 ± 15	45 ± 13	
		Final	46 ± 13	45 ± 13	
		Change	4 ± 7	0 ± 12	
**HADS**		n	20	17	0 (-3 to 4)
		Baseline	16 ± 10	15 ± 9	
		Final	14 ± 10	13 ± 9	
		Change	-2 ± 5	-2 ± 6	
**POS-R**	Global severity score	n	20	18	2 (-3 to 7)
		Baseline	19 ± 14	19 ± 14	
		Final	18 ± 14	20 ± 14	
		Change	1 ± 6	-1 ± 9	
	mean severity	n	20	18	0 (0 to 0)
		Baseline	2 ± 1	2 ± 1	
		Final	2 ± 1	2 ± 1	
		Change	0 ± 0	0 ± 0	
	mean number	n	22	16	0 (-1 to 2)
		Baseline	9 ± 4	10 ± 4	
		Final	9 ± 4	10 ± 5	
		Change	0 ± 4	0 ± 2	
**ESES**		n	19	16	0 (-1 to 1)
		Baseline	2 ± 2	2 ± 1	
		Final	2 ± 1	2 ± 1	
		Change	0 ± 1	0 ± 1	
**DPPEBBS**		n	19	15	3 (-4 to 11)
		Baseline	59 ± 10	59 ± 15	
		Final	61± 10	65 ± 7	
		Change	2 ± 7	6 ± 14	
**DASI**		n	20	18	4.93 (-0.94 to 10.80)
		Baseline	13.06 ± 12.85	20.29 ± 14.33	
		Final	17.29 ± 14.41	19.60 ± 14.59	
		Change	4.22 ± 9.72	-0.71 ± 7.92	

Abbreviations: CI, confidence interval; DASI, Duke Activity Status Index; DPPEBBS, Dialysis Patients Benefits and Barriers Scale; ESES, Exercise Self efficacy Scale; HADS, Hospital Anxiety and Depression Scale; MCS, mental component summary score; POS-R, Palliative Outcomes Scale Renal, PCS, physical component summary score; VAS, visual analogue scale.

For peer review only

Supplementary material 10. Baseline demographic and clinical characteristics for the qualitative participants.

		N=25
**Age** (years)		69±10
**Gender** n (%)	Female	13 (52%)
	Male	12 (48%)
**Ethnicity** n (%)	White background	13 (52%)
	Asian or Asian British	10 (40%)
	Caribbean	1 (4%)
	Not stated	1 (4%)
**Diagnosis**	Diabetic nephropathy	11 (44%)
	Aetiology uncertain	6 (24%)
	Chronic pyelonephritis	3 (12%)
	Atypical hemolytic uremic syndrome	1 (4%)
	FSGS	1 (4%)
	Henoch-Schönlein Purpura	1 (4%)
	Minimal change nephropathy	1 (4%)
	Polycystic kidney disease	1 (4%)
**CCI**		6±2
**Time on HD** (months)		43 (IQR 16-85)
**CFS** n (%)	Vulnerable	9 (36%)
	Mildly frail	5 (20%)
	Moderately frail	8 (32%)
	Severely frail	3 (12%)
**Number of falls in the last six months**		3 (IQR 2-4)
**Previous transplant** n (%)	No	21 (84%)
	Yes	4 (16%)
**Active on transplant list** n (%)	No	22 (88%)
	Yes	3 (12%)

Abbreviations: CCI, Charlson comorbidity index; CFS, clinical frailty scale; FSGS, Focal segmental glomerulosclerosis; HD, haemodialysis.

For peer review only

Supplementary material 11. Joint display of quantitative and qualitative results, with an overall assessment of mixed-methods inferences.

	Progression criteria	Feasibility trial	Qualitative results	Mixed-methods inferences
**Eligibility**	**STOP** <20%<br>**GO** >50%<br>eligible.	31% patients eligible	No discussion. Patients not involved in screening process	Silence
**Recruitment**	**STOP** <25%<br>**GO** >50%<br>recruited.	52% eligible patients recruited.	- Frailer and female participants less likely to be approached despite eligibility and have more concerns about their suitability<br>- Perception that risks outweigh the potential benefits<br>- Recruitment processes could be improved	Complementary
**Retention**	**STOP** >40%<br>**GO** <20%<br>lost to follow-up.	12% loss to follow-up. Reasons predominantly unavoidable (death, ill-health).	Loss to follow-up attributed to:<br>- Illness;<br>- Length of trial;<br>- Study not meeting expectations.	Complementary
**Intervention**	**STOP** <30%<br>**GO** >70%<br>adherence over six-months.	74% adherence rate across the six-month exercise duration.	- IDC good use of time but limited in scope.<br>- Participants felt safe and felt well supported.<br>- Participants described a range of other important exercise components (see Figure 2)	Complementary
**Outcome**	**STOP** <70%<br>**GO** >80%<br>outcome measure completion.	Up to 89% of secondary outcome measure data missing<br>Collection of falls data challenging.	- Number of outcomes measured viewed as excessive.<br>- Outcome testing during HD or at home preferred.<br>- 52% agreed to complete a falls diary, falls not prioritised by participants.<br>- STS60, ESWT and ISWT unsuitable<br>- Researcher support and family involvement may increase outcome measure completion<br>- Outcomes measuring ADLs and participation in social roles prioritised	Complementary<br><br>Silence for PA monitoring.

Results from the feasibility trial are colour coded to depict whether they met the 'stop' (red), 'go' (green) or 'change (orange) progression criteria. Abbreviations: ADLs, activities of daily living; ESWT, Endurance Shuttle Walk Test; IDC, intradialytic exercise; ISWT, Incremental Shuttle Walk Test; PA, physical activity; STS60, sit to stand in sixty seconds.

For peer review only - http://bmjopen.bmj.com/site/about/guidelines.xhtml

CONSORT 2010 checklist of information to include when reporting a pilot or feasibility trial*

Section/Topic	Item No	Checklist item	Reported on page No
**Title and abstract**			
	1a	Identification as a pilot or feasibility randomised trial in the title	1
	1b	Structured summary of pilot trial design, methods, results, and conclusions (for specific guidance see CONSORT abstract extension for pilot trials)	3-4
**Introduction**			
Background and objectives	2a	Scientific background and explanation of rationale for future definitive trial, and reasons for randomised pilot trial	6
	2b	Specific objectives or research questions for pilot trial	6-7
**Methods**			
Trial design	3a	Description of pilot trial design (such as parallel, factorial) including allocation ratio	7-8
	3b	Important changes to methods after pilot trial commencement (such as eligibility criteria), with reasons	n/a
Participants	4a	Eligibility criteria for participants	7 and supplementary material 1
	4b	Settings and locations where the data were collected	7-8,10-11
	4c	How participants were identified and consented	8
Interventions	5	The interventions for each group with sufficient details to allow replication, including how and when they were actually administered	8 and supplementary material 2
Outcomes	6a	Completely defined prespecified assessments or measurements to address each pilot trial objective specified in 2b, including how and when they were assessed	9-11, supplementary materials 3,4 and 5
	6b	Any changes to pilot trial assessments or measurements after the pilot trial commenced, with reasons	n/a
	6c	If applicable, prespecified criteria used to judge whether, or how, to proceed with future definitive trial	9 and supplementary material 3
Sample size	7a	Rationale for numbers in the pilot trial	8-9

For peer review only

	7b	When applicable, explanation of any interim analyses and stopping guidelines	9
Randomisation:			
Sequence generation	8a	Method used to generate the random allocation sequence	8
	8b	Type of randomisation(s); details of any restriction (such as blocking and block size)	8
Allocation concealment mechanism	9	Mechanism used to implement the random allocation sequence (such as sequentially numbered containers), describing any steps taken to conceal the sequence until interventions were assigned	8
Implementation	10	Who generated the random allocation sequence, who enrolled participants, and who assigned participants to interventions	8
Blinding	11a	If done, who was blinded after assignment to interventions (for example, participants, care providers, those assessing outcomes) and how	9
	11b	If relevant, description of the similarity of interventions	n/a
Statistical methods	12	Methods used to address each pilot trial objective whether qualitative or quantitative	11
**Results**			
Participant flow (a diagram is strongly recommended)	13a	For each group, the numbers of participants who were approached and/or assessed for eligibility, randomly assigned, received intended treatment, and were assessed for each objective	12-13 figure 1
	13b	For each group, losses and exclusions after randomisation, together with reasons	12-13 figure 1
Recruitment	14a	Dates defining the periods of recruitment and follow-up	12
	14b	Why the pilot trial ended or was stopped	n/a
Baseline data	15	A table showing baseline demographic and clinical characteristics for each group	Table 1 (trial) supplementary material 10 (qualitative)
Numbers analysed	16	For each objective, number of participants (denominator) included in each analysis. If relevant, these numbers should be by randomised group	Supplementary materials6-9 and page 15-17
Outcomes and estimation	17	For each objective, results including expressions of uncertainty (such as 95% confidence interval) for any estimates. If relevant, these results should be by randomised group	Supplementary materials 6-9 and page 15-17
Ancillary analyses	18	Results of any other analyses performed that could be used to inform the future definitive trial	17- 26
Harms	19	All important harms or unintended effects in each group (for specific guidance see CONSORT for harms)	P 17
	19a	If relevant, other important unintended consequences	n/a
**Discussion**			

For peer review only

Limitations	20	Pilot trial limitations, addressing sources of potential bias and remaining uncertainty about feasibility	30
Generalisability	21	Generalisability (applicability) of pilot trial methods and findings to future definitive trial and other studies	26-31
Interpretation	22	Interpretation consistent with pilot trial objectives and findings, balancing potential benefits and harms, and considering other relevant evidence	26-31
	22a	Implications for progression from pilot to future definitive trial, including any proposed amendments	26-31
**Other information**			
Registration	23	Registration number for pilot trial and name of trial registry	4 and 7
Protocol	24	Where the pilot trial protocol can be accessed, if available	7
Funding	25	Sources of funding and other support (such as supply of drugs), role of funders	31
	26	Ethical approval or approval by research review committee, confirmed with reference number	32

Citation: Eldridge SM, Chan CL, Campbell MJ, Bond CM, Hopewell S, Thabane L, et al. CONSORT 2010 statement: extension to randomised pilot and feasibility trials. BMJ. 2016;355.
*We strongly recommend reading this statement in conjunction with the CONSORT 2010, extension to randomised pilot and feasibility trials, Explanation and Elaboration for important clarifications on all the items. If relevant, we also recommend reading CONSORT extensions for cluster randomised trials, non-inferiority and equivalence trials, non-pharmacological treatments, herbal interventions, and pragmatic trials. Additional extensions are forthcoming: for those and for up to date references relevant to this checklist, see www.consort-statement.org.

For peer review only
